# An Annular Conductive Membrane-Based Hollow Capacitive Wind Pressure Sensor: Analytical Solution and Numerical Design and Calibration

**DOI:** 10.3390/ma18050965

**Published:** 2025-02-21

**Authors:** Jun-Yi Sun, Zhi-Qiang Yan, He-Hao Feng, Xiao-Ting He

**Affiliations:** 1 School of Civil Engineering, Chongqing University, Chongqing 400045, China; 202216131314t@stu.cqu.edu.cn (Z.-Q.Y.); 202216131289t@stu.cqu.edu.cn (H.-H.F.); hexiaoting@cqu.edu.cn (X.-T.H.); 2 Key Laboratory of New Technology for Construction of Cities in Mountain Area, Ministry of Education (Chongqing University), Chongqing 400045, China

**Keywords:** annular conductive membrane, wind pressure, large deflection, analytical solution, hollow capacitive sensor

## Abstract

A novel hollow capacitive wind pressure sensor is for the first time proposed. The sensing element of the proposed sensor uses a non-parallel plate variable capacitor, whose movable electrode plate uses a transversely uniformly loaded annular conductive membrane with a fixed outer edge and a rigid inner edge (acting as the wind pressure sensitive element of the sensor). Due to the unique hollow configuration of the proposed sensor, it can be used alone to detect the pressure exerted by fast-moving air in the atmosphere or by fast-moving air or gas, etc., in pipes, but it also can be used in pairs to measure the flow rate of fast-moving air or gas, etc., in pipes. The analytical solution of the large deflection elastic behavior of the transversely uniformly loaded annular conductive membrane is derived by using a new set of membrane governing equations. The effectiveness of the new analytical solution is analyzed. The new membrane governing equations are compared with the previous ones to show the differences between them. The superiority of the new analytical solution over the existing ones is analyzed. An example is given to demonstrate the numerical design and calibration of the proposed sensor and the effect of changing design parameters on the important capacitance–pressure (*C*–*q*) analytical relationship of the proposed sensor is investigated comprehensively. Finally, an experimental verification of the analytical solution derived is carried out.

## 1. Introduction

Wind is a natural phenomenon due to the movement of air in the atmosphere, which is usually related to the radiant heat of the sun and the water source. The strength of the wind is usually expressed in terms of how quickly the air is moving, namely the wind speed [1,2], but sometimes by the pressure exerted on a stationary surface per unit area by the moving air, which is called the wind pressure [3,4]. The relationship between the wind pressure (denoted by *P*) and the wind speed (denoted by *V*) is often approximated by *P* = 0.125 *V*^2^. However, this is obviously a very rough estimation because, at the same speed, the humidity, density, or physical or chemical composition of the air has a great influence on the wind pressure. After all, at the same speed, the greater the mass of the air per unit volume, the greater its inertia. On the other hand, however, in the vast majority of applications, the real concern or interest is actually not the wind speed but often the wind pressure, the pressure exerted on a stationary surface per unit area by the fast-moving air [5,6,7,8,9,10].

Wind pressure measurement can be achieved by many types of pressure sensors, such as piezoresistive pressure sensors, piezoelectric pressure sensors, resonant pressure sensors, optical fiber pressure sensors, and capacitive pressure sensors. These pressure sensors also play an important role in various applications in industry, agriculture, civil engineering, mining, aerospace, ecological environment protection, and scientific research. Piezoresistive pressure sensors are developed based on the piezoresistive effect of monocrystalline silicon [11,12,13,14,15]. Piezoelectric pressure sensors are pressure sensors based on the piezoelectric effect [16,17,18,19,20]. Resonant pressure sensors use resonant elements to convert the pressure to be measured into frequency signals [21,22,23,24,25]. Optical fiber pressure sensors are developed based on the relationships between the propagation characteristics of light in optical fiber materials and the pressure acting on photoelastic elements [26,27,28,29,30]. In comparison with other types of sensors, capacitive sensors have attracted much attention due to their advantages, such as good versatility, low sensitivity to temperature, and low power consumption [31,32,33,34,35,36].

A capacitive sensor uses a variable capacitor to detect the non-electrical signal and convert it into capacitance as an electrical output signal. As is well known, a capacitor consists of a pair of electrode plates facing each other and a dielectric between the two mutually facing electrode plates. The capacitance of the capacitor depends on the size of the gap between the mutually facing electrode plates and the size of the dielectric constant of the dielectric, and thus, it can be changed by changing the size of the gap or by replacing the dielectric. Therefore, a variable capacitor can be a capacitor whose dielectric remains constant and the gap is variable, or a capacitor whose gap remains constant and the dielectric is variable, or a capacitor whose dielectric and gap are both variable. However, the first of these three types of variable capacitors is more commonly used, where the two mutually facing electrode plates are often two mutually parallel rigid flat plates, and such a variable capacitor is thus called a parallel plate variable capacitor [37,38,39,40,41]. One of the two electrode plates of a parallel plate variable capacitor is fixed, called a fixed electrode plate, and the other is movable, called a movable electrode plate. Of course, the two mutually facing electrode plates of the first type of variable capacitor mentioned above can also be two soft plates, as used in soft sensors [42,43,44,45,46], or can be a combination of a fixed rigid electrode plate and a movable soft electrode plate with a fixed outer edge, as used in some non-touch or touch mode capacitive pressure sensors [47,48,49,50,51].

In this study, the non-parallel plate variable capacitor that has a fixed rigid electrode plate and a movable soft electrode plate with a fixed outer edge is modified into a hollow non-parallel plate variable capacitor that has a fixed annular rigid electrode plate and a movable annular soft electrode plate (an annular conductive membrane) with a fixed outer edge and a rigid inner edge. Such a hollow non-parallel plate variable capacitor is then used to detect the wind pressure and convert it into capacitance as an electrical output signal, achieving the so-called hollow capacitive wind pressure sensor. The hollow configuration of the capacitive wind pressure sensor proposed here has the advantage of allowing the fast-moving fluids to pass directly through, which creates the conditions for the pressure detection of the fast-moving gas or air, etc., in pipes. Therefore, this hollow capacitive wind pressure sensor can be used alone to directly detect the pressure exerted by the fast-moving air in the atmosphere or by the fast-moving gas or air, etc., in pipes, but also can be used in pairs to form a throttling differential pressure flowmeter to measure the flow rate of the fast-moving gas or air, etc., in pipelines (however, the detail of this flow rate measurement is beyond the scope of this article).

The remainder of the paper is organized as follows. In the following section, the structure and working principle of the proposed hollow capacitive wind pressure sensor are introduced, and the important analytical relationship between the capacitance *C* and pressure *q* of the proposed sensor is derived, where the capacitance *C* is expressed as the function of the deflection *w* of the movable annular soft electrode plate (the annular conductive membrane) with a fixed outer edge and a rigid inner edge, and the pressure *q* is included in the expression of the deflection *w*. In Section 3, in order to give a more accurate expression of the deflection *w*, the large deflection elastic behavior of the annular conductive membrane under the pressure *q* is analytically re-solved by using a new set of membrane governing equations, resulting in a new analytical solution of the problem. In Section 4, some important issues are discussed, such as the effectiveness of the new analytical solution, the difference between the new and previous membrane governing equations, the difference between the new and previous analytical solutions, and in addition to this, an example is given to demonstrate how to use the new analytical solution to carry out the numerical design and calibration of the proposed hollow capacitive wind pressure sensor, the effect of changing some important design parameters on its input/output analytical relationship of the proposed sensor is investigated comprehensively, and finally, experimental verification of the analytical solution derived is carried out. Concluding remarks are shown in Section 5.

At present, the pressure measurement of the fast-moving gas or air in a pipeline is achieved mainly by piezoresistive or piezoelectric pressure sensors. The existing capacitive pressure sensors, due to their unsuitable configurations, cannot be used for the pipeline pressure measurement. Given that capacitive sensors have advantages such as good versatility, low sensitivity to temperature, and low power consumption, we here use the hollow configuration for developing the hollow capacitive pressure sensor, thus solving the difficulty that the existing capacitive pressure sensors cannot be directly used for pipeline pressure measurement. Therefore, from this point of view, it is meaningful to carry out research on the hollow capacitive wind pressure sensor proposed here, which can fill this application gap.

## 2. Materials and Methods

The term “wind pressure” refers to the action of the fast-moving air on the surface of an object. As an action force, the wind pressure is always in the same direction as the fast-moving air. This is clearly different from the static gas pressure, whose direction is always perpendicular to the surface being acted upon, no matter how deformed the surface is. Therefore, when applied onto the surface of an initially flat and peripherally fixed membrane, the wind pressure should be regarded as the uniformly distributed transverse loads of the membrane, while the static gas pressure should be regarded as the uniformly distributed normal loads of the membrane, no matter how deflected the membrane is. In other words, the elastic response (stress, strain, and displacement) of the same membrane under transverse loading is different from that under normal loading, i.e., their analytical relationships between deformation and loading are different. This suggests that the mechanical mechanism or principle of using the same membrane structure to measure the wind pressure is different from that of measuring the static gas pressure.

**Figure 1:**
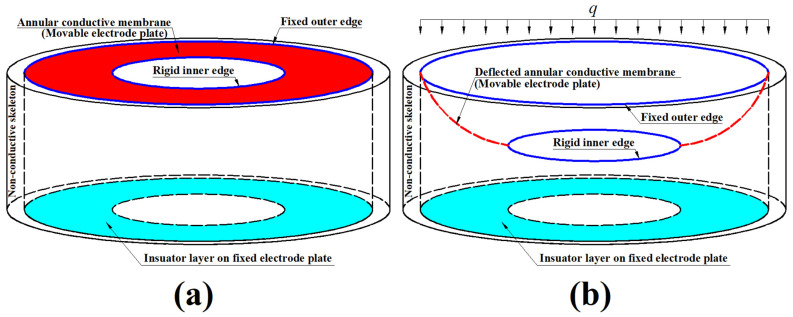
Schematic of the proposed hollow capacitive wind pressure sensor, where (**a**) the case before the uniformly distributed transverse loads *q* is applied; (**b**) the case after the uniformly distributed transverse loads *q* is applied.

The structure and working principle of the hollow capacitive wind pressure sensor proposed are shown in Figure 1. An initially flat annular conductive membrane, whose outer edge is fixed at a hollow non-conductive cylinder skeleton and whose inner edge is attached to a weightless rigid ring, is used as the movable electrode plate of a parallel plate capacitor (whose fixed electrode plate is coated with an insulator layer), as shown in Figure 1a. Then, it is subjected to the action of the uniformly distributed transverse loads *q*, i.e., the wind pressure exerted by the fast-moving air on the annular membrane, resulting in the membrane’s deflection toward the direction of the loads *q* (against the wind direction), as shown in Figure 1b. Such an axisymmetric, non-uniform deflection toward the insulator layer on the fixed electrode plate makes the capacitor between the movable and fixed electrode plates change from the original parallel plate capacitor (see Figure 1a) to the current non-parallel plate capacitor (see Figure 1b), thus achieving a non-parallel plate variable capacitor.

**Figure 2:**
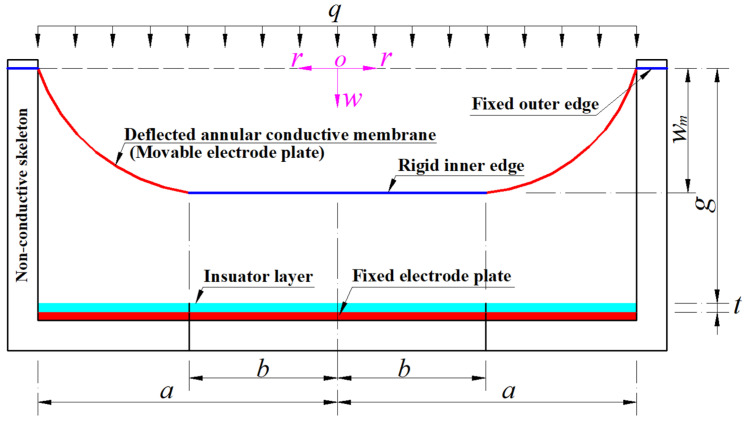
A profile along a diameter of the proposed hollow capacitive wind pressure sensor under the wind pressure *q*.

Since the gap between the movable and fixed electrode plates is reduced, the capacitance of the non-parallel plate variable capacitor between the movable and fixed electrode plates is increased. Obviously, the capacitance value of the non-parallel plate variable capacitor between the movable and fixed electrode plates corresponds, one-to-one, to the value of the uniformly distributed transverse loads *q*, i.e., the wind pressure exerted by the fast-moving air on the movable electrode plate. Therefore, as long as the one-to-one correspondence analytical relationship between pressure and capacitance can be obtained in advance, the value of the wind pressure exerted on the movable electrode plate can be determined by measuring the capacitance value of the non-parallel plate variable capacitor between the movable and fixed electrode plates. This is the working principle of the hollow capacitive wind pressure sensor proposed. To this end, the hollow capacitive wind pressure sensor is parameterized, as shown in Figure 2, where the dashed line represents the geometric middle plane of the initially flat annular conductive membrane (the movable electrode plate that has not yet been subjected to the wind pressure *q*) whose outer radius is *a* and inner radius is *b*, the thickness of the insulator layer coating on the fixed electrode plate is denoted by *t*, the initially parallel gap between the initially flat annular conductive membrane and the insulator layer is denoted by *g*, the polar plane (*r*, *φ*) of the introduced cylindrical coordinate system (*r*, *φ*, *w*) is located in the plane in which the geometric middle plane is located, the coordinate origin *o* is at the centroid of the geometric middle plane, the radial, circumferential and transverse coordinates are, respectively, *r*, *φ* (*φ* cannot be shown due to the profile) and *w*, also *w* denotes the deflection (transverse displacement) of the annular conductive membrane under the wind pressure *q*, and *w_m_* denotes the maximum deflection of the annular conductive membrane under the wind pressure *q*.

The capacitance of the non-parallel plate variable capacitor between the movable and fixed electrode plates, assuming to be denoted by *C*, consists of two parts of capacitance: one part is the capacitance of the parallel plate capacitor between the fixed electrode plate and the insulator layer on the fixed electrode plate, denoted by *C*_1_, and the other part is the capacitance of the non-parallel plate capacitor between the movable electrode plate and the insulator layer on the fixed electrode plate, denoted by *C*_2_. Let us denote the vacuum permittivity as *ε*_0_, the relative permittivity of the insulator layer as *ε_r_*_1_, and the relative permittivity of the air as *ε_r_*_2_, then the capacitance *C*_1_ may be written as

(1) $$C_{1}=\frac{\varepsilon_{0}\varepsilon_{r1}\pi (a^{2}-b^{2})}{t},$$

and the capacitance *C*_2_ may be written as

(2) $$C_{2}=\int_{b}^{a}\int_{0}^{2\pi }\varepsilon_{0}\varepsilon_{r2}\frac{r}{g-w(r)}d\varphi dr=2\pi \varepsilon_{0}\varepsilon_{r2}\int_{b}^{a}\frac{r}{g-w(r)}dr.$$

Since the capacitance *C*_1_ and capacitance *C*_2_ are connected in series, the capacitance *C* of the non-parallel plate variable capacitor between the movable and fixed electrode plates is given by

(3) $$C=\frac{C_{1}C_{2}}{C_{1}+C_{2}}=\frac{\frac{\varepsilon_{0}\varepsilon_{r1}\pi {\left(a-b\right)}^{2}}{t}2\pi \varepsilon_{0}\varepsilon_{r2}\int_{b}^{a}\frac{r}{g-w(r)}dr}{\frac{\varepsilon_{0}\varepsilon_{r1}\pi {\left(a-b\right)}^{2}}{t}+2\pi \varepsilon_{0}\varepsilon_{r2}\int_{b}^{a}\frac{r}{g-w(r)}dr}.$$

Obviously, the deflection function *w*(*r*) depends strictly on the wind pressure *q* exerted on the movable electrode plate; that is, they are the one-to-one correspondence analytical relationship. Therefore, it can be concluded from Equation (3) that the capacitance *C* and the wind pressure *q* are also the one-to-one correspondence analytical relationship (denoted as the pressure–capacitance (*p–C*) analytical relationship, i.e., the pressure *q* is used as an input variable (an independent variable), and the capacitance *C* is used as an output variable (a dependent variable)). The analytical expression of the deflection function *w*(*r*) can be determined by analytically solving the large deflection elastic behavior of the annular conductive membrane (the movable electrode plate) under the wind pressure *q*. As long as the large deflection elastic behavior can be solved analytically, the analytical relationship between the capacitance *C* and the wind pressure *q* can be determined, then the implementation of the proposed hollow capacitive wind pressure sensor is possible. The analytical solution to the large deflection elastic behavior is organized into a separate section, as shown in the next section.

## 3. Analytical Solution to the Elastic Behavior of the Movable Electrode Plate

**Figure 3:**
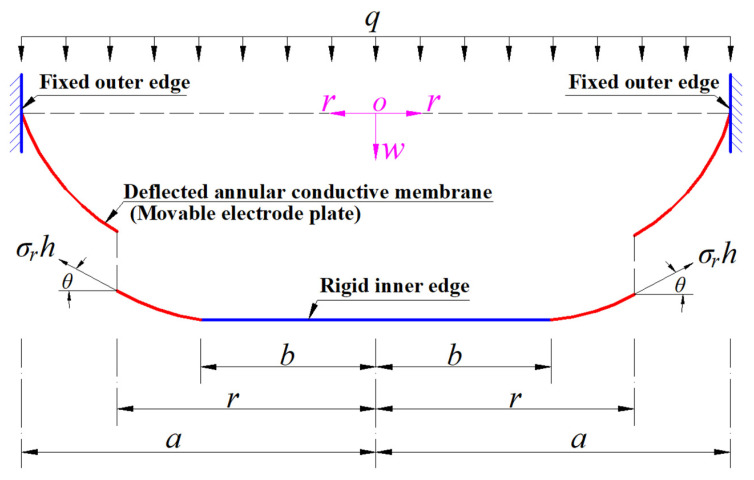
A free body with radius *r* (*b* ≤ *r* ≤ *a*) taken from the central region of the deflected annular conductive membrane in Figure 2.

Suppose that the outer radius, inner radius, thickness, Poisson’s ratio, and Young’s modulus of elasticity of the annular conductive membrane (the movable electrode plate) in Figure 1 or Figure 2 are denoted by *a*, *b*, *h*, *v*, and *E*, respectively. A free body is taken from the central region of the deflected annular conductive membrane subjected to the wind pressure *q* (see Figure 2) to study its static problem of equilibrium, as shown in Figure 3, where *σ_r_* is the radial stress at *r*, *σ_r_h* is the membrane force acting on the boundary *r*, and *θ* denotes the rotation angle of the annular membrane at *r*.

In the direction perpendicular to the polar plane (*r*, *φ*), there are two vertical forces acting on the free body; they are the downward external force *π*(*r*^2^ − *b*^2^)*q* generated by the wind pressure *q* and the upward component 2*πrσ_r_hsinθ* of the membrane force 2*πrσ_r_h* acting at the boundary *r*. Then, the condition of the resultant force being equal to zero in the vertical direction gives

(4) $$\pi (r^{2}-b^{2})q-2\pi r\sigma_{r}h\sin\theta =0,$$

where since 0 ≤ *θ* ≤ *π*/2 (see Figure 3), then

(5) $$\sin\theta =\sin\theta /\sqrt{\mathrm{co}s^{2}\theta +\sin^{2}\theta }=\tan\theta /\sqrt{1+\tan^{2}\theta }=(-dw/dr)/\sqrt{1+{\left(-dw/dr\right)}^{2}}.$$

After substituting Equation (5) into Equation (4), the out-of-plane equilibrium equation can be written as

(6) $$q(r^{2}-b^{2})\sqrt{1+{\left(-dw/dr\right)}^{2}}-2r\sigma_{r}\;h(-dw/dr)=0.$$

The in-plane equilibrium equation is given by [52].

(7) $$\frac{d}{dr}(\frac{r\sigma_{r}}{\sqrt{1+{\left(-dw/dr\right)}^{2}}})-\sigma_{t}\sqrt{1+{\left(-dw/dr\right)}^{2}}=0.$$

The geometric equations, that is, the relationships between the radial strain *e_r_* and circumferential strain *e_t_* and radial displacement *u*, are given by [52]

(8) $$e_{r}={\left[{\left(1+\frac{du}{dr}\right)}^{2}+{\left(-\frac{dw}{dr}\right)}^{2}\right]}^{1/2}-1$$

and

(9) $$e_{t}=\frac{u}{r}.$$

The relationships between stress and strain (i.e., the physical equations) for Hookean-type materials follow Hooke’s law [52]

(10) $$e_{r}=\frac{1}{E}(\sigma_{r}-\nu \sigma_{t})$$

and

(11) $$e_{t}=\frac{1}{E}(\sigma_{t}-\nu \sigma_{r}).$$

The six physical quantities *σ_r_*, *σ_t_*, *e_r_*, *e_t_*, *u*, and *w* can be determined by simultaneously solving Equations (6)–(11). Eliminating *e_r_* and *e_t_* from Equations (8)–(11) yields

(12) $${\left[{\left(1+\frac{du}{dr}\right)}^{2}+{\left(-\frac{dw}{dr}\right)}^{2}\right]}^{1/2}-1=\frac{1}{E}(\sigma_{r}-\nu \sigma_{t})$$

and

(13) $$\frac{u}{r}=\frac{1}{E}(\sigma_{t}-\nu \sigma_{r}).$$

By means of Equations (12) and (13), the radial stress *σ_r_* and circumferential stress *σ_t_* can be expressed as

(14) $$\sigma_{r}=\frac{E}{1-\nu^{2}}\{\nu \frac{u}{r}+{\left[{\left(1+\frac{du}{dr}\right)}^{2}+{\left(-\frac{dw}{dr}\right)}^{2}\right]}^{1/2}-1\}$$

and

(15) $$\sigma_{t}=\frac{E}{1-\nu^{2}}\{\frac{u}{r}+\nu {\left[{\left(1+\frac{du}{dr}\right)}^{2}+{\left(-\frac{dw}{dr}\right)}^{2}\right]}^{1/2}-\nu \}.$$

Substituting the *u* in Equation (13) into Equation (14) yields

(16) $${\left(\frac{1}{E}\sigma_{r}-\frac{v}{E}\sigma_{t}+1\right)}^{2}-{\left[\frac{1}{E}\frac{d(r\sigma_{t})}{dr}-\frac{v}{E}\frac{d(r\sigma_{r})}{dr}+1\right]}^{2}-{\left(-\frac{dw}{dr}\right)}^{2}=0.$$

Equations (6), (7), and (16) are three equations for solving *σ_r_*, *σ_t_*, and *w*, and the boundary conditions for determining the special solutions of *σ_r_*, *σ_t_*, and *w* are

(17) $$e_{t}=\frac{u}{r}=\frac{1}{E}[\sigma_{t}-\nu \sigma_{r}]=0\;\mathrm{at}\;r=b,$$

(18) $$e_{t}=\frac{u}{r}=\frac{1}{E}[\sigma_{t}-\nu \sigma_{r}]=0\;\mathrm{at}\;r=a$$

and

(19) w=0 at r=a.

After introducing the following dimensionless variables

(20) $$Q=\frac{aq}{hE},W=\frac{w}{a},S_{r}=\frac{\sigma_{r}}{E},S_{t}=\frac{\sigma_{t}}{E},\alpha =\frac{b}{a},x=\frac{r}{a},$$

Equations (6), (7), (13), and (16)–(19) can be transformed into

(21) $$Q(x^{2}-\alpha^{2})\sqrt{1+{\left(-dW/dx\right)}^{2}}-2xS_{r}(-dW/dx)=0,$$

(22) $$\frac{d}{dx}(\frac{xS_{r}}{\sqrt{1+{\left(-dW/dx\right)}^{2}}})-S_{t}\sqrt{1+{\left(-dW/dx\right)}^{2}}=0,$$

(23) $$\frac{u}{r}=S_{t}-\nu S_{r},$$

(24) $${\left(S_{r}-vS_{t}+1\right)}^{2}-{\left[\frac{d(xS_{t})}{dx}-v\frac{d(xS_{r})}{dx}+1\right]}^{2}-{\left(-\frac{dW}{dx}\right)}^{2}=0,$$

(25) $$S_{t}-\nu S_{r}=0\;\mathrm{at}\;x=\alpha ,$$

(26) $$S_{t}-\nu S_{r}=0\;\mathrm{at}\;x=1$$

and

(27) W=0 at x=1.

Since the stress and displacement are finite within *α* ≤ *x* ≤ 1, then *S_r_*, *S_t_*, and *W* can be expanded as the power series of the *x* − (1 + *α*)/2. After introducing *β* = (1 + *α*)/2 and *X* = *x* − *β*, it is found that

(28) $$S_{r}=\sum_{i=0}^{\infty }b_{i}{\left(x-\beta \right)}^{i}=\sum_{i=0}^{\infty }b_{i}X^{i},$$

(29) $$S_{t}=\sum_{i=0}^{\infty }c_{i}{\left(x-\beta \right)}^{i}=\sum_{i=0}^{\infty }c_{i}X^{i}$$

and

(30) $$W=\sum_{i=0}^{\infty }d_{i}{\left(x-\beta \right)}^{i}=\sum_{i=0}^{\infty }d_{i}X^{i}.$$

For the convenience of solution, we introduce the following intermediate variables

(31) $$F=\sqrt{1+{\left(-dW/dx\right)}^{2}}=\sum_{i=0}^{\infty }f_{i}{\left(x-\beta \right)}^{i}=\sum_{i=0}^{\infty }f_{i}X^{i}$$

and

(32) $$H=\frac{xS_{r}}{\sqrt{1+{\left(-dW/dx\right)}^{2}}}=\sum_{i=0}^{\infty }h_{i}{\left(x-\beta \right)}^{i}=\sum_{i=0}^{\infty }h_{i}X^{i}.$$

Further, use *X* = *x* − *β* to transform Equations (21), (22), (24), (31), and (32) into

(33) $$Q[{\left(X+\beta \right)}^{2}-\alpha^{2}]F-2(X+\beta )S_{r}(-\frac{dW}{dX})=0,$$

(34) $$\frac{dH}{dX}-S_{t}F=0,$$

(35) $${\left(S_{r}-vS_{t}+1\right)}^{2}-{\left[(X+\beta )\frac{dS_{t}}{dX}+S_{t}-v(X+\beta )\frac{dS_{r}}{dX}-vS_{r}+1\right]}^{2}-{\left(-\frac{dW}{dX}\right)}^{2}=0,$$

(36) $$F^{2}-{\left(-\frac{dW}{dX}\right)}^{2}-1=0$$

and

(37) $$HF-(X+\beta )S_{r}=0.$$

After substituting the power series expansions of the functions *S_r_*, *S_t_*, *W*, *F,* and *H* with respect to the *X* in Equations (28)–(32) into Equations (33)–(37), the recursive formulas of the power series coefficients *b_i_*, *c_i_*, *d_i_*, *f_i_*, and *h_i_* can be obtained, which are shown in Appendix B.

As can be seen from Appendix B, the power series coefficients *b_i_*, *c_i_*, and *d_i_*, where *i* = 1, 2, 3, …, and *f_i_* and *h_i_*, where *i* = 0, 1, 2, 3, …, can be expressed as polynomials with respect to the coefficients *b*_0_ and *c*_0_. The coefficients *b*_0_ and *c*_0_ are usually called undetermined constants, and the remaining coefficient *d*_0_ is a dependent undetermined constant that depends on the undetermined constants *b*_0_ and *c*_0_. All the undetermined constants, including *b*_0_, *c*_0_, and *d*_0_, can be determined by using the boundary conditions Equations (25)–(27). From Equations (28) and (29), Equations (25) and (26) give

(38) $$\sum_{i=0}^{\infty }c_{i}{\left(\alpha -\beta \right)}^{i}-\nu \sum_{i=1}^{\infty }b_{i}{\left(\alpha -\beta \right)}^{i}=0$$

and

(39) $$\sum_{i=0}^{\infty }c_{i}{\left(1-\beta \right)}^{i}-\nu \sum_{i=1}^{\infty }b_{i}{\left(1-\beta \right)}^{i}=0.$$

Since the power series coefficients *b_i_* and *c_i_*, where *i* = 1, 2, 3, …, can be expressed as polynomials with respect to the coefficients *b*_0_ and *c*_0_, the undetermined constants *b*_0_ and *c*_0_ can be determined by simultaneously solving Equations (38) and (39). In addition, from Equation (30), Equation (27) gives

(40) $$d_{0}=-\sum_{i=1}^{\infty }d_{i}{\left(1-\beta \right)}^{i}.$$

Similarly, since the power series coefficients *d_i_*, where *i* = 1, 2, 3, …, can be expressed as polynomials with respect to the coefficients *b*_0_ and *c*_0_, then, with the known *b_0_* and *c_0_*, the dependent undetermined constants *d*_0_ can be determined by Equation (40).

After the undetermined constants *b*_0_, *c*_0_, and *d*_0_ are known, all the power series coefficients *b_i_*, *c_i_*, and *d_i_* (*i* = 1, 2, 3, …,) can be determined, and all the expressions of *S_r_*, *S_t_*, and *W* can thus be determined. Finally, from Equations (20), (28)–(30), the dimensional analytical expressions of *σ_r_*, *σ_t_*, and *w* can be written as

(41) $$\sigma_{r}=E\sum_{i=0}^{\infty }b_{i}{\left(x-\beta \right)}^{i}=E\sum_{i=0}^{\infty }b_{i}{\left(\frac{r}{a}-\frac{a+b}{2a}\right)}^{i},$$

(42) $$\sigma_{t}=E\sum_{i=0}^{\infty }c_{i}{\left(x-\beta \right)}^{i}=E\sum_{i=0}^{\infty }c_{i}{\left(\frac{r}{a}-\frac{a+b}{2a}\right)}^{i}$$

and

(43) $$w=a\sum_{i=0}^{\infty }d_{i}{\left(x-\beta \right)}^{i}=a\sum_{i=0}^{\infty }d_{i}{\left(\frac{r}{a}-\frac{a+b}{2a}\right)}^{i}.$$

## 4. Results and Discussion

In this section, some important issues are addressed, including whether there are derivation errors in the analytical solution derived in Section 3 (see Section 4.1 for details), how the new membrane governing equation differs from the previous ones (see Section 4.2 for details), how the new analytical solution differs from the previous ones (see Section 4.3 for details), how to use the new analytical solution to numerically design and calibrate the hollow capacitive wind pressure sensor proposed (see Section 4.4 for details), how does changing the design parameters affect the capacitance–pressure (*C*–*q*) analytical relationships (see Section 4.5 for details). In addition, an experimental verification of the analytical solution derived in Section 3 is carried out (see Section 4.6 for details).

### 4.1. The Effectiveness of the New Analytical Solution Derived in Section 3

The correctness or effectiveness of the new analytical solution of hollow annular membranes derived in Section 3 can be verified with the aid of the well-established analytical solution of circular membranes in the literature, which is detailed as follows. Suppose that the outer radius *a*, thickness *h*, Poisson’s ratio *v*, and Young’s modulus of elasticity *E* of the hollow annular membrane with a fixed outer edge and a rigid inner edge (which is considered in Section 3) are the same as those of a circular membrane with a fixed outer edge, and that the two membranes are subjected to the same uniformly distributed transverse loads *q*. If the deflection curve of the hollow annular membrane (which is calculated by the new analytical solution of hollow annular membranes derived in Section 3) can gradually approach that of the circular membrane (which is calculated by the well-established analytical solution of circular membranes in the literature) as the inner radius *b* of the hollow annular membrane gradually decreases from the outer radius *a* to zero, then such an asymptotic behavior suggests that the new analytical solution of hollow annular membranes derived in Section 3 is basically correct or effective, because the hollow annular membrane will become the circular membrane when the inner radius *b* of the hollow annular membrane is equal to zero.

**Figure 4:**
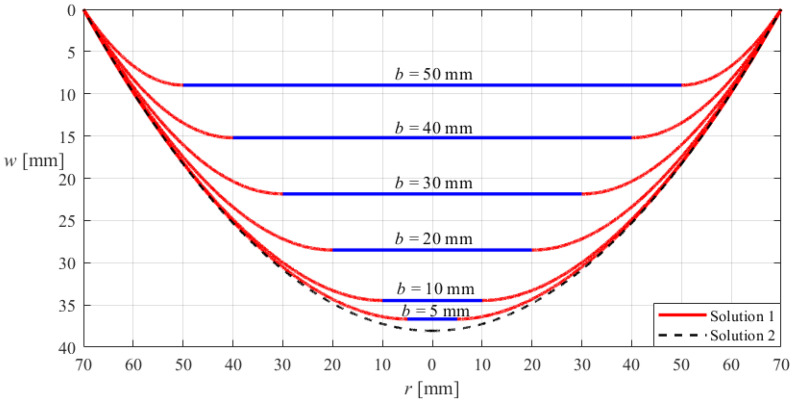
Sketch of the asymptotic behavior from the hollow annular membrane with the outer radius *a* = 70 mm to the circular membrane with the outer radius *a* = 70 mm when the inner radius *b* of the annular membrane takes 50 mm, 40 mm, 30 mm, 20 mm, 10 mm, and 5 mm, respectively, where the annular and circular membranes are both subjected to the same uniformly distributed transverse loads *q* = 0.012 MPa and both have the same thickness *h* = 0.2 mm, Poisson’s ratio *v* = 0.47, and Young’s modulus of elasticity *E* = 7.84 MPa, and “Solution 1” refers to the deflection curves of the annular membrane calculated by the new analytical solution of hollow annular membranes derived in Section 3, while “Solution 2” refers to the deflection curve of the circular membrane calculated by the analytical solution of circular membranes presented in [52].

Figure 4 illustrates such an asymptotic behavior, where the annular and circular membranes are both subjected to the action of the uniformly distributed transverse loads *q* = 0.012 MPa, and both have the outer radius *a* = 70 mm, thickness *h* = 0.2 mm, Poisson’s ratio *v* = 0.47, and Young’s modulus of elasticity *E* = 7.84 MPa, while the inner radius *b* of the annular membrane takes 50 mm, 40 mm, 30 mm, 20 mm, 10 mm, and 5 mm, respectively. In Figure 4, “Solution 1” refers to the deflection curves of the annular membrane calculated by the new analytical solution of hollow annular membranes derived in Section 3, while “Solution 2” refers to the deflection curve of the circular membrane calculated by the analytical solution of circular membranes presented in [52].

It can be seen from Figure 4 that the asymptotic behavior from the annular membrane to the circular membrane is very perfect, suggesting that the new analytical solution of hollow annular membranes derived in Section 3 is effective and, to a certain extent, reliable.

### 4.2. The Comparison Between the New and Previous Membrane Governing Equations

The large deflection problem of the hollow annular membrane subjected to the uniformly distributed transverse loads *q*, which is dealt with in Section 3, has been analytically solved for three times, which can be found in [53,54,55]. The membrane governing equations used in this paper and in [53,54,55] are summarized in Table 1 for comparison, where the physical equations used in [53,54,55] are the same as the ones used in this paper (i.e., Equations (10) and (11)), which are not listed in Table 1.

It can be seen from Table 1 that if the term (−d*w*/d*r*) is so small that (−d*w*/d*r*) → 0, then the term (1 + (−d*w*/d*r*)^2^)^1/2^ can replaced by 1, which makes the out-of-plane and in-plane equilibrium equations after improvement regress to the ones before improvement; that is, the improved out-of-plane equilibrium equation used in this paper and used in [54,55] can be regressed to the previous one used in [53], and the improved in-plane equilibrium equation used in this paper can be regressed to the previous one used in [53,54,55]. On the other hand, from Figure 3, it can be seen that (−d*w*/d*r*) = tan*θ*, where *θ* denotes the rotation angle of the annular membrane at *r*. Therefore, tan*θ* → 0 when (−d*w*/d*r*) → 0, that is, the rotation angle of the membrane, *θ*, is small enough when (−d*w*/d*r*) → 0. This means that the improvement to the out-of-plane equilibrium equation used in [53] and to the in-plane equilibrium equation used in [53,54,55] is achieved by giving up the assumption of the small rotation angle of membrane used in the previous derivation of the out-of-plane and in-plane equilibrium equations. In fact, the improvement to the geometric equations used in [53,54] is achieved also by giving up the small rotation angle assumption of the membrane, see [56] for details. Therefore, the adoption of the small rotation angle assumption of the membrane should be the main reason for the loss of computational accuracy of the analytical solutions presented in [53,54,55].

In addition, it can also be seen from Table 1 that since the physical equations used in [53,54,55] are the same as the ones used in this paper (i.e., Equations (10) and (11)), then, the difference between the analytical solutions presented in [53,54] should be caused mainly by the difference between the two out-of-plane equilibrium equations used in [35,37], the difference between the analytical solutions presented in [54,55] should be caused mainly by the difference between the two geometric equations used in [54,55], and the difference between the new analytical solution presented in this paper and the one presented in [55] should be caused mainly by the difference between the in-plane equilibrium equation used in this paper and the one used in [55].

### 4.3. The Comparison Between the New and Previous Analytical Solutions

**Figure 5:**
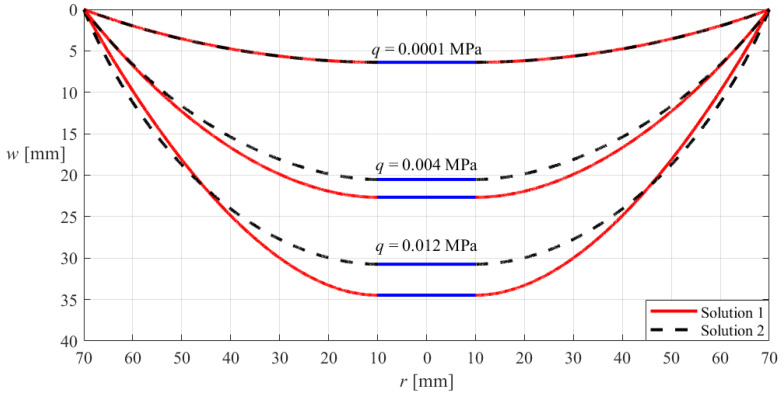
The variation of the membrane deflection *w* with the radial coordinate *r*, where “Solution 1” refers to the deflection calculation results by the analytical solution newly derived in Section 2 in this paper, and “Solution 2” refers to the deflection calculation results by the analytical solution previously derived in [55].

It can be seen from Table 1 that since the membrane governing equations used in [55] are more accurate than those used in [54], while the membrane governing equations used in [54] are more accurate than those used in [53], then, the analytical solution presented in [55] should be more accurate than the one presented in [54], while the analytical solution presented in [54] should be more accurate than the one presented in [53], as shown in Figure 21 in [55]. Therefore, here, we only need to compare the analytic solution presented in this paper with the one presented in [55]. To this end, the two analytical solutions presented in this paper and in [55] are used to calculate the deflections of a hollow annular membrane with Young’s modulus of elasticity *E* = 7.84 MPa, Poisson’s ratio *v* = 0.47, thickness *h* = 0.2 mm, inner radius *b* = 10 mm, and outer radius *a* = 70 mm, where the hollow annular membrane is subjected to the action of *q* = 0.0001 MPa, 0.004 MPa, 0.012 MPa, respectively. The calculation results are shown in Figure 5, where “Solution 1” refers to the deflection calculation results by the analytical solution newly derived in Section 2 in this paper, and “Solution 2” refers to the deflection calculation results by the analytical solution previously derived in [55].

It can be seen from Figure 5 that the two solutions agree quite closely when the uniformly distributed transverse loads *q* takes 0.0001 MPa but diverge when the uniformly distributed transverse loads *q* takes 0.004 MPa, especially 0.012 MPa. This suggests that the small rotation angle assumption of the membrane adopted in the in-plane equilibrium equation in [55] still has a large influence on the computational accuracy of the analytical solution.

### 4.4. A Demonstration of Numerical Design and Calibration

In this section, an example is given to demonstrate how to carry out the numerical design and calibration of the proposed hollow capacitive wind pressure sensor based on the analytical solution derived in Section 3.

The wind pressure (uniformly distributed transverse loads) that needs to be measured is assumed to be about *q* = 0~0.0543296 MPa. The numerical design and calibration of the proposed hollow capacitive wind pressure sensor starts with a preliminary determination of material and dimension design parameters.

The inner radius *a* of the hollow capacitive wind pressure sensor to be designed (i.e., the outer radius of its annular conductive membrane (its movable electrode plate, see Figure 2)), the inner radius *b*, Poisson’s ratio *v*, and Young’s modulus of elasticity *E* of the annular conductive membrane, and the relative permittivity *ε_r_*_1_ and thickness *t* of the insulation layer are the main design parameters that need to be preliminarily determined (note that, after the preliminary determination of the annular conductive membrane, its yield strength *σ_y_* is also determined). Assumed that these design parameters are preliminarily determined as *a* = 70 mm, *b* = 50 mm, *v* = 0.47, *E* = 7.84 MPa, *σ_y_* = 4.6 MPa, *ε_r_*_1_ = 2.5 (the insulator layer is assumed to use polyethylene) and *t* = 0.1 mm. In addition, the air relative permittivity is *ε_r_*_2_ = 1.00053, and the vacuum permittivity is *ε*_0_ = 8.854 × 10^−3^ pF/mm.

After the above preliminary determination, the thickness *h* of the annular conductive membrane and the initially parallel gap *g* between the insulator layer and the initially flat annular conductive membrane (the movable electrode plate before loading) can be determined according to the maximum wind pressure *q* = 0.0543296 MPa and the yield strength *σ_y_* = 4.6 MPa.

The thickness *h* can first take an arbitrary value, for example, taking *h* = 0.2 mm. Then, using the recursion formulas for the power series coefficients *b_i_*, *c_i_*, *d_i_*, *f_i_*, and *h_i_* in Appendix B and *q* = 0.0543296 MPa, *a* = 70 mm, *b* = 50 mm, *h* = 0.2 mm, *v* = 0.47, and *E* = 7.84 MPa, the undetermined constants *b*_0_, *c*_0_, and *d*_0_ can be determined by Equations (21), (31), and (32), resulting *b*_0_ = 0.49280338, *c*_0_ = 0.26921538, and *d*_0_ = 0.17129919. Further, with the known *b*_0_, *c*_0_, and *d*_0_, the radial stress *σ_r_* and circumferential stress *σ_t_* can be calculated from Equation (33) and Equation (34), respectively, in order to determine the maximum membrane stress *σ_m_* (the largest of the maximum value of the radial stress *σ*_r_ and the maximum value of the circumferential stress *σ_t_*). The calculation result is that the maximum value of the radial stress *σ_r_* is the largest, so *σ_m_* = 3.15 MPa. If the maximum membrane stress *σ_m_* in this calculation is between 0.5*σ_y_* and 0.7*σ_y_*, then the arbitrarily taken *h* value meets the requirement of material strength. If the maximum membrane stress *σ_m_* in this calculation is much less than 0.5*σ_y_* or much greater than 0.7*σ_y_*, then the arbitrarily taken *h* value does not meet the requirement of material strength. In this case, a slightly reduced or increased *h* value is needed to recalculate the maximum membrane stress *σ_m_* until the material strength requirement is met. Now, since *σ_m_* = 3.15 MPa and *σ_y_* = 4.6 MPa, then 0.5*σ_y_* ≤ *σ_m_* ≤ 0.7*σ_y_*. Therefore, the arbitrarily taken *h* value in this calculation, *h* = 0.2 mm, can meet the material strength requirement.

After the determination of *h* = 0.2 mm, the initially parallel gap *g* between the insulator layer and the initially flat annular conductive membrane (the movable electrode plate before loading) can be determined using the maximum deflection *w_m_* that is produced by the annular membrane with *a* = 70 mm, *b* = 50 mm, *h* = 0.2 mm, *v* = 0.47, and *E* = 7.84 MPa under *q* = 0.0543296 MPa. The maximum deflection *w_m_* can be calculated by Equation (43), using *b*_0_ = 0.49280338, *c*_0_ = 0.26921538, and *d*_0_ = 0.17129919 and the recursion formulas for the power series coefficients *b_i_*, *c_i_*, *d_i_*, *f_i_*, and *h_i_* in Appendix B, resulting *w_m_* = 16.265 mm. Therefore, the initially parallel gap *g* may be taken as *g* = 17 mm, which means that the initially parallel gap *g* should be greater than and close to the maximum deflection *w_m_* = 16.265 mm.

After the above hardware numerical design of the sensor, the numerical calibration of the sensor can be carried out, as detailed below.

Since the mechanism of the proposed hollow capacitive wind pressure sensor is to detect the wind pressure *q*, which is applied to the sensor, by measuring the capacitance *C* of the sensor under the wind pressure *q*, then, such a wind pressure measurement system should require a capacitance–pressure (*C*–*p*) analytical relationship (i.e., the capacitance *C* is used as an input variable (an independent variable) and the wind pressure *q* is used as an output variable (a dependent variable)), rather than the pressure–capacitance (*p–C*) analytical relationship shown in Equation (3). However, such a capacitance–pressure (*C*–*p*) analytical relationship cannot be derived directly from Equation (3) because Equation (3) is a transcendental equation with respect to the wind pressure *q* (the wind pressure *q* is included in the deflection function *w*(*r*), see Equations (20) and (43) and the recursion formulas for the power series coefficients *b_i_*, *c_i_*, *d_i_*, *f_i_*, and *h_i_* in Appendix B).

**Figure 6:**
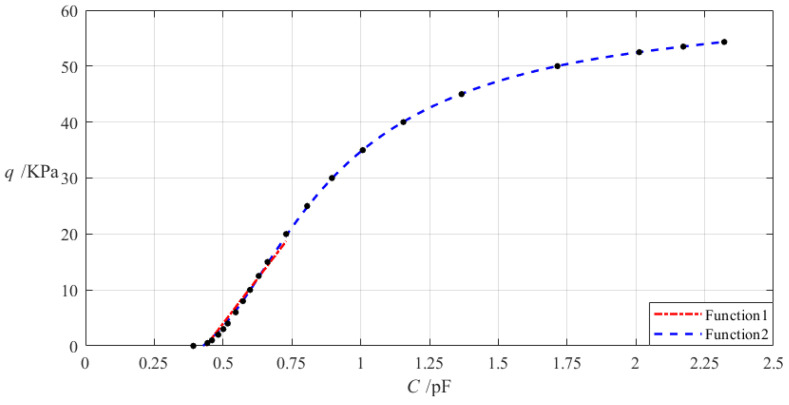
The capacitance–pressure (*C–p*) relationship of the sensor with *a* = 70 mm, *b* = 50 mm, *h* = 0.2 mm, *E* = 7.84 MPa, *ν* = 0.47, *t* = 0.1 mm, and *g* = 17 mm and its least-squares data fitting, where “Function 1” and “Function 2” are fitted, respectively, by a straight line and a curve, whose analytical expressions are listed in Table 2.

Therefore, the required capacitance–pressure (*C*–*p*) analytical relationship needs to be obtained through the least-squares data fitting based on the large number of numerical calculation values for the capacitance *C*. To this end, the numerical calculations need to start with a smaller wind pressure value and end at *q* = 0.0543296 MPa, including the numerical values of the undetermined constants *b_0_*, *c_0_*, and *d_0_*, maximum deflection *w_m_*, maximum stress *σ_m_*, and capacitance *C*, as shown in Table A1 in Appendix A. After the numerical calculations, the capacitance–pressure (*C*–*p*) analytical relationship can be obtained by using the least-square method to fit the numerical calculation values of the pressure *q* and capacitance *C* in Table A1, as “Function 1” and “Function 2” in Figure 6, which is fitted with a straight line and a curve. The obtained analytical expressions of “Function 1” and “Function 2” in Figure 6 are shown in Table 2.

The linear analytical expression of the capacitance–pressure (*C–q*) analytical relationship, as “Function 1” in Figure 6 and Table 2, is simple and therefore suitable for analog techniques, while the nonlinear analytical expression of the capacitance–pressure (*C–q*) analytical relationship, as “Function 2” in Figure 6 and Table 2, is complex and therefore suitable only for digital techniques.

However, if the capacitance–pressure (*C–q*) analytical relationship obtained above, “Function 1” or “Function 2” in Figure 6 and Table 2, does not meet the use requirements or technical needs (including the variation range of the wind pressure *q* or capacitance *C* or the ratio of the wind pressure *q* to capacitance *C*), some (or just one) of the design parameters (including the outer radius *a*, inner radius *b*, thickness *h*, Poisson’s ratio *v*, and Young’s modulus of elasticity *E* of the annular conductive membrane, etc.) need to be adjusted, and then, the above numerical design and calibration need to restart, until the use requirements or technical needs are met. So, in this case, it is necessary to know in advance how changes in the design parameters affect the *C–q* analytical relationships, which is addressed in the following section.

### 4.5. The Effect of Changing Design Parameters on the C–q Analytical Relationships

In this section, we will change the design parameters of the proposed hollow capacitive wind pressure sensor one by one and examine their effects on the capacitance–pressure (*C–q*) analytical relationship. The design parameters to be changed include the outer radius *a*, inner radius *b*, thickness *h*, Young’s modulus of elasticity *E*, and Poisson’s ratio *v* of the annular conductive membrane, the thickness *t* of the insulator layer, and the initially parallel gap *g* between the initially flat annular conductive membrane and the insulator layer. The values of these design parameters, which are used in Section 4.4, are still used here, i.e., *a* = 70 mm, *b* = 50 mm, *h* = 0.2 mm, *E* = 7.84 MPa, *ν* = 0.47, *t* = 0.1 mm, and *g* = 17 mm, also including *ε_r_*_1_ = 2.5, *ε_r_*_2_ = 1.00053, and *ε*_0_ = 8.854 × 10^−3^ pF/mm.

#### 4.5.1. Influence of Changing Outer Radius *a* on *C*–*q* Relationships

**Figure 7:**
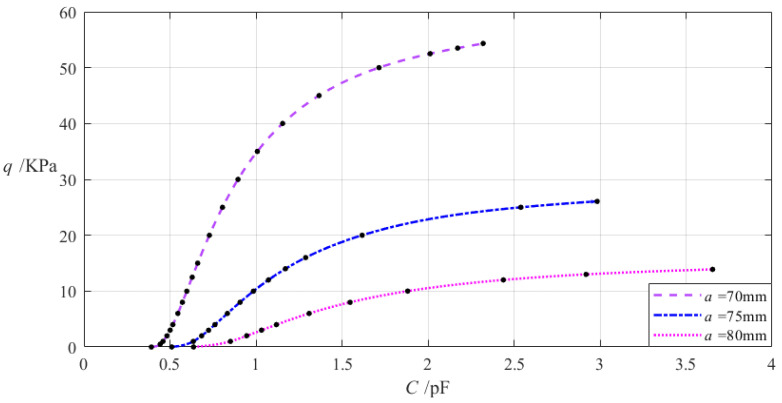
The effect of changing the annular membrane outer radius *a* on the *C–q* relationships when *b* = 50 mm, g = 17 mm, *h* = 0.2 mm, *t* = 0.1 mm, *E* = 7.84 MPa, *ν* = 0.47, and *a* takes 70 mm, 75 mm, and 80 mm, respectively.

The design parameters in this section are *b* = 50 mm, *h* = 0.2 mm, *E* = 7.84 MPa, *v* = 0.47, *t* = 0.1 mm, *g* = 17 mm, *ε*_0_ = 8.854 × 10^−3^ pF/mm, *ε_r_*_2_ = 1.00053, *ε_r_*_1_ = 2.5, and the outer radius *a* of the annular conductive membrane takes 70 mm, 75 mm, and 80 mm, respectively. When the wind pressure *q* takes different values, the results of numerical calculation of the undetermined constants *b*_0_, *c*_0_, and *d*_0_, maximum membrane deflection *w_m_*, maximum membrane stress *σ_m_*, and capacitance *C* are listed in Table A1 for *a* = 70 mm, in Table A2 for *a* = 75 mm, and in Table A3 for *a* = 80 mm. Figure 7 shows the effect of changing the outer radius *a* of the annular conductive membrane on the *C–q* analytical relationships.

It can be seen from Figure 7 that as the outer radius *a* increases, the variation range of the input capacitance *C* increases while the variation range of the output wind pressure *q* decreases. The degree of influence of increasing the outer radius *a* on the variation ranges of the input capacitance *C* and output wind pressure *q* can be simply measured by the percentage of the two relative increments of the input capacitance *C* and outer radius *a* and the percentage of the two relative increments of the output wind pressure *q* and outer radius *a*, respectively. The relative increment of the outer radius *a* is equal to (80 mm − 70 mm)/70 mm = 14.29%, and from Table A1 and Table A3, the relative increment of the input capacitance *C* is (3.655 pF − 2.321 pF)/2.321 pF = 57.47%, and the relative decrement of the output wind pressure *q* is (13.897 KPa − 54.329 KPa)/54.329 KPa = −74.42%. Therefore, the degree of influence of increasing the outer radius *a* on the variation range of the input capacitance *C* is equal to 57.47%/14.29% = 402.17%, and the degree of influence of increasing the outer radius *a* on the variation range of the output wind pressure *q* is equal to −74.42%/14.29% = −520.78%; that is, a 1% increase in the outer radius *a* will result in a 4.0217% increase in the variation range of the input capacitance *C* and a 5.2078% decrease in the variation range of the output wind pressure *q*.

#### 4.5.2. Influence of Changing Inner Radius *b* on *C*–*q* Relationships

**Figure 8:**
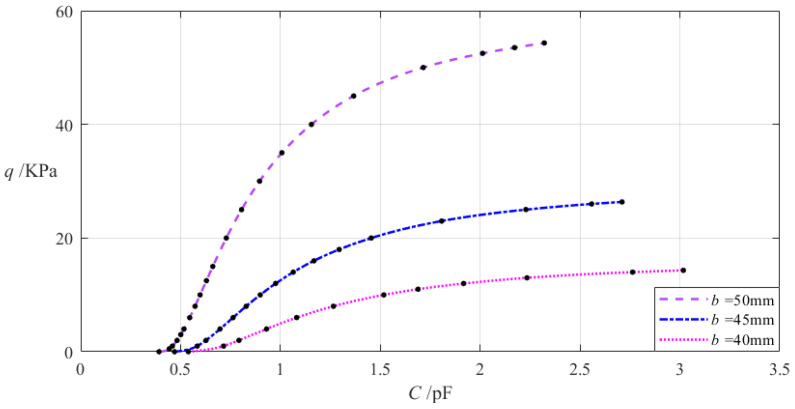
The effect of decreasing the annular membrane inner radius *b* on the *C–q* relationships when *a* = 70 mm, *g* = 17 mm, *h* = 0.2 mm, *t* = 0.1 mm, *E* = 7.84 MPa, ν = 0.47, and *b* takes 50 mm, 45 mm, and 40 mm, respectively.

The relevant design parameters in this section are *a* = 70 mm, *h* = 0.2 mm, *E* = 7.84 MPa, *v* = 0.47, *t* = 0.1 mm, *g* = 17 mm, *ε*_0_ = 8.854 × 10^−3^ pF/mm, *ε_r_*_2_ = 1.00053, *ε_r_*_1_ = 2.5, and the inner radius *b* of the annular conductive membrane takes 50 mm, 45 mm, and 40 mm, respectively. When the wind pressure *q* takes different values, the results of numerical calculation of the undetermined constants *b*_0_, *c*_0_, and *d*_0_, maximum membrane deflection *w_m_*, maximum membrane stress *σ_m_*, and capacitance *C* are listed in Table A1 for *b* = 50 mm, in Table A4 for *b* = 45 mm, and in Table A5 for *b* = 40 mm. Figure 8 shows the effect of changing the inner radius *b* of the annular conductive membrane on the *C–q* analytical relationships.

It can be seen from Figure 8 that as the inner radius *b* decreases, the variation range of the input capacitance *C* increases while the variation range of the output wind pressure *q* decreases. The relative increment of the inner radius *b* is (40 mm − 50 mm)/50 mm = −20%, and from Table A1 and Table A5, the relative increment of the input capacitance *C* is (3.018 pF − 2.321 pF)/2.321 pF = 30.03%, and the relative decrement of the output wind pressure *q* is (14.323 KPa − 54.329 KPa)/54.329 KPa = −73.64%. Therefore, the degree of influence of decreasing the inner radius *b* on the variation range of the input capacitance *C* is equal to 30.03%/(−20%) = −150.15%, and the degree of influence of decreasing the inner radius *b* on the variation range of the output wind pressure *q* is equal to −73.64%/(−20%) = 368.20%; that is, a 1% decrease in the inner radius *b* will result in a 1.5015% increase in the variation range of the input capacitance *C* and a 3.682% decrease in the variation range of the output wind pressure *q*.

#### 4.5.3. Influence of Changing Membrane Thickness *h* on *C*–*q* Relationships

**Figure 9:**
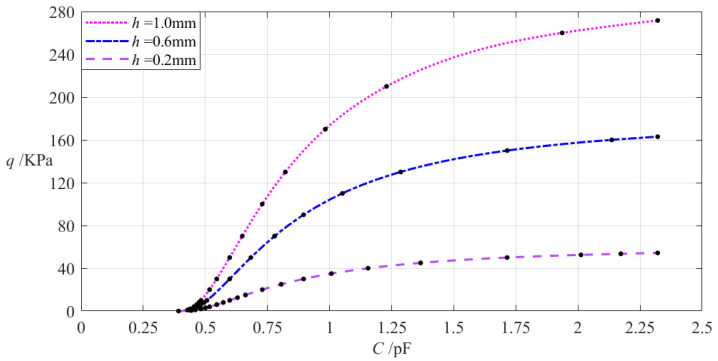
The effect of changing the membrane thickness *h* on the *C–q* relationships when *a* = 70 mm, *b* = 50 mm, *g* = 17 mm, *t* = 0.1 mm, ν = 0.47, and *h* takes 0.2 mm, 0.6 mm, and 1 mm, respectively.

The relevant design parameters in this section are *a* = 70 mm, *b* = 50 mm, *E* = 7.84 MPa, *v* = 0.47, *t* = 0.1 mm, *g* = 17 mm, *ε*_0_ = 8.854 × 10^−3^ pF/mm, *ε_r_*_2_ = 1.00053, *ε_r_*_1_ = 2.5, and the thickness *h* of the annular conductive membrane takes 0.2 mm, 0.6 mm, and 1 mm, respectively. When the wind pressure *q* takes different values, the results of numerical calculation of the undetermined constants *b*_0_, *c*_0_, and *d*_0_, maximum membrane deflection *w_m_*, maximum membrane stress *σ_m_*, and capacitance *C* are listed in Table A1 for *h* = 0.2 mm, in Table A6 for *h* = 0.6 mm, and in Table A7 for *h* = 1 mm. Figure 9 shows the effect of changing the thickness *h* of the annular conductive membrane on the *C–q* analytical relationships.

It can be seen from Figure 9 that as the membrane thickness *h* increases, the variation range of the input capacitance *C* is almost unchanged while the variation range of the output wind pressure *q* increases. The relative increment of the membrane thickness *h* is (1 mm − 0.2 mm)/0.2 mm = 400%, and from Table A1 and Table A7, the relative decrement of the output wind pressure *q* is (271.648 Kpa − 54.329 KPa)/54.329 KPa = 400%. Therefore, the degree of influence of increasing the membrane thickness *h* on the variation range of the output wind pressure *q* is equal to 400%/400% = 100%; that is, a 1% increase in the membrane thickness *h* will result in a 1% increase in the variation range of the output wind pressure *q* and will almost not change the variation range of the input capacitance *C*.

#### 4.5.4. Influence of Changing Young’s Modulus of Elasticity *E* on *C*–*p* Relationships

**Figure 10:**
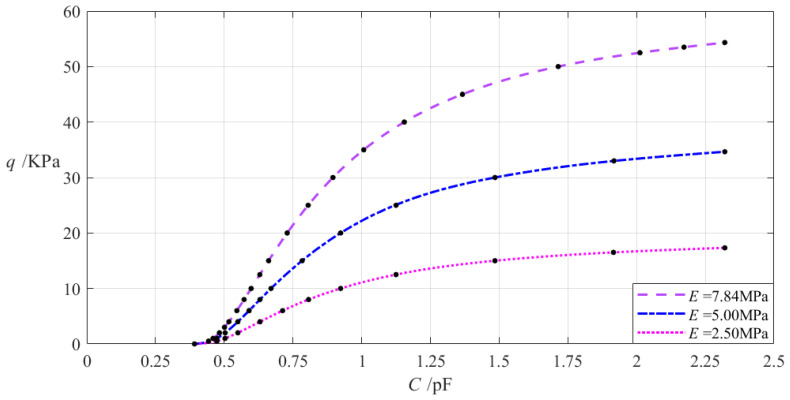
The effect of changing the Young’s modulus of elasticity *E* on the *C–q* relationships when *a* = 70 mm, *b* = 50 mm, *g* = 17 mm, *h* = 0.2 mm, *t* = 0.1 mm, ν = 0.47, and *E* takes 7.84 MPa, 5 MPa, and 2.5 MPa, respectively.

The relevant design parameters in this section are *a* = 70 mm, *b* = 50 mm, *h* = 0.2 mm, *v* = 0.47, *t* = 0.1 mm, *g* = 17 mm, *ε*_0_ = 8.854 × 10^−3^ pF/mm, *ε_r_*_2_ = 1.00053, *ε_r_*_1_ = 2.5, and the Young’s modulus of elasticity *E* of the annular conductive membrane takes 7.84 MPa, 5 MPa, and 2.5 MPa, respectively. When the wind pressure *q* takes different values, the results of numerical calculation of the undetermined constants *b_0_*, *c_0_*, and *d_0_*, maximum membrane deflection *w_m_*, maximum membrane stress *σ_m_*, and capacitance *C* are listed in Table A1 for *E* = 7.84 MPa, in Table A8 for *E* = 5 MPa, and in Table A9 for *E* = 2.5 MPa. Figure 10 shows the effect of changing the Young’s modulus of elasticity *E* of the annular conductive membrane on the *C–q* analytical relationships.

It can be seen from Figure 10 that as the Young’s modulus of elasticity *E* decreases, the variation range of the input capacitance *C* is almost unchanged while the variation range of the output wind pressure *q* decreases. The relative increment of the Young’s modulus of elasticity *E* is (2.5 MPa − 7.84 MPa)/7.84 MPa = −68.11%, and from Table A1 and Table A9, the relative decrement of the output wind pressure *q* is (17.324 Kpa − 54.329 KPa)/54.329 KPa = −68.11%. Therefore, the degree of influence of decreasing the Young’s modulus of elasticity *E* on the variation range of the output wind pressure *q* is equal to −68.11%/(−68.11%) = 1%; that is, a 1% decrease in the Young’s modulus of elasticity *E* will result in a 1% decrease in the variation range of the output wind pressure *q* and will almost not change the variation range of the input capacitance *C*.

#### 4.5.5. Influence of Changing Poisson’s Ratio *v* on *C*–*q* Relationships

**Figure 11:**
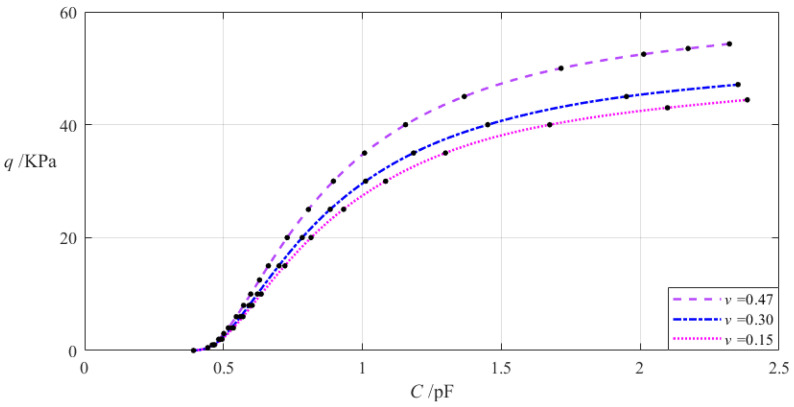
The effect of changing the Poisson’s ratio *v* on the *C–q* relationships when *a* = 70 mm, *b* = 50 mm, *g* = 17 mm, *h* = 0.2 mm, *t* = 0.1 mm, *E* = 7.84 MPa, and *v* takes 0.47, 0.3, and 0.15, respectively.

The relevant design parameters in this section are *a* = 70 mm, *b* = 50 mm, *h* = 0.2 mm, *E* = 7.84 MPa, *t* = 0.1 mm, *g* = 17 mm, *ε*_0_ = 8.854 × 10^−3^ pF/mm, *ε_r_*_2_ = 1.00053, *ε_r_*_1_ = 2.5, and the Poisson’s ratio *v* of the annular conductive membrane takes 0.47, 0.3, and 0.15, respectively. When the wind pressure *q* takes different values, the results of numerical calculation of the undetermined constants *b*_0_, *c*_0_, and *d*_0_, maximum membrane deflection *w_m_*, maximum membrane stress *σ_m_*, and capacitance *C* are listed in Table A1 for *v* = 0.47, in Table A10 for *v* = 0.3, and in Table A11 for *v* = 0.15. Figure 11 shows the effect of changing the Poisson’s ratio *v* of the annular conductive membrane on the *C–q* analytical relationships.

It can be seen from Figure 11 that as the Poisson’s ratio *v* decreases, the variation range of the input capacitance *C* slightly increases while the variation range of the output wind pressure *q* slightly decreases. The relative increment of the Poisson’s ratio *v* is (0.15 − 0.47)/0.47 = −68.09%, and from Table A1 and Table A11, the relative increment of the input capacitance *C* is (2.385 pF − 2.321 pF)/2.321 pF = 2.76%, and the relative decrement of the output wind pressure *q* is (44.403 KPa − 54.329 KPa)/54.329 KPa = −18.27%. Therefore, the degree of influence of decreasing the Poisson’s ratio *v* on the variation range of the input capacitance *C* is equal to 2.76%/(−68.09%) = −4.05%, and the degree of influence of decreasing the Poisson’s ratio *v* on the variation range of the output wind pressure *q* is equal to −18.27%/(−68.09%) = 26.83%; that is, a 1% decrease in the Poisson’s ratio *v* will result in a 0.0405% increase in the variation range of the input capacitance *C* and a 0.2683% decrease in the variation range of the output wind pressure *q*.

#### 4.5.6. Influence of Changing Insulator Layer Thickness *t* on *C*–*q* Relationships

**Figure 12:**
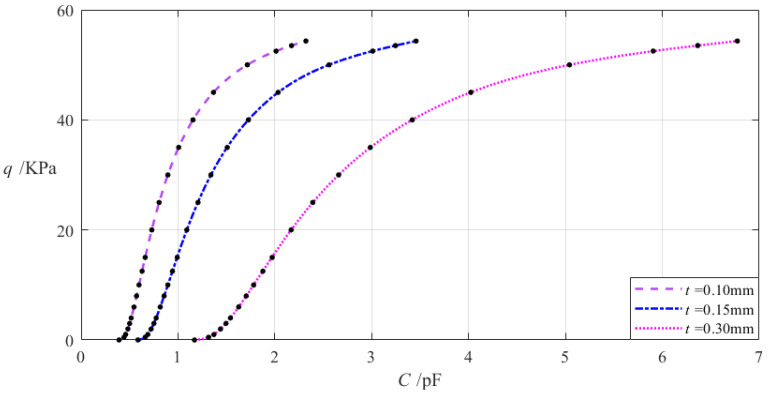
The effect of changing the insulator layer thickness *t* on the *C–q* relationships when *a* = 70 mm, *b* = 50 mm, *g* = 17 mm, *h =* 0.2 mm, *E* = 7.84 MPa, ν = 0.47, and *t* takes 0.1 mm, 0.15 mm, and 0.2 mm, respectively.

The relevant design parameters in this section are *a* = 70 mm, *b* = 50 mm, *h* = 0.2 mm, *E* = 7.84 MPa, *v* = 0.47, *g* = 17 mm, *ε*_0_ = 8.854 × 10^−3^ pF/mm, *ε_r_*_2_ = 1.00053, *ε_r_*_1_ = 2.5, and the thickness *t* of the insulator layer takes 0.1 mm, 0.15 mm, and 0.3 mm, respectively. When the wind pressure *q* takes different values, the results of numerical calculation of the undetermined constants *b*_0_, *c*_0_, and *d*_0_, maximum membrane deflection *w_m_*, maximum membrane stress *σ_m_*, and capacitance *C* are listed in Table A1 for *t* = 0.1 mm, in Table A12 for *t* = 0.15 mm, and in Table A13 for *t* = 0.3 mm. Figure 12 shows the effect of changing the insulator layer thickness *t* on the *C–q* analytical relationships.

It can be seen from Figure 12 that as the insulator layer thickness *t* increases, the variation range of the input capacitance *C* increases while the variation range of the output wind pressure *q* is almost unchanged. The relative increment of the insulator layer thickness *t* is (0.3 mm − 0.1 mm)/0.1 mm = 200%, and the relative decrement of the input capacitance *C* is equal to [(6.774 pF − 1.170 pF) − (2.321 pF − 0.392 pF)]/(2.321 pF − 0.392 pF) = 190.51%, which is calculated from Table A1 and Table A13. Therefore, the degree of influence of increasing the insulator layer thickness *t* on the variation range of the input capacitance *C* is equal to 191.86%/200% = 95.26%; that is, a 1% increase in the insulator layer thickness *t* will result in a 0.9526% increase in the variation range of the input capacitance *C* and will almost not change the variation range of the output wind pressure *q*.

#### 4.5.7. Influence of Changing Initially Parallel Gap *g* on *C*–*q* Relationships

**Figure 13:**
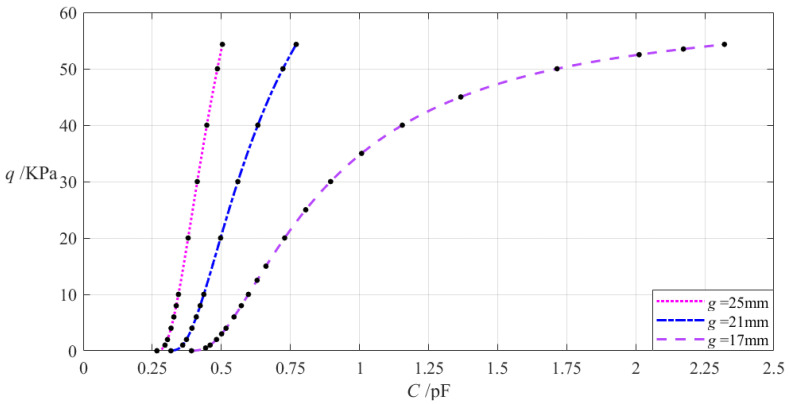
The effect of changing the initially parallel gap *g* on the *C*–*q* relationships when *a* = 70 mm, *b* = 50 mm, *h* = 0.2 mm, *t* = 0.1 mm, *E* = 7.84 MPa, ν = 0.47, and *g* takes 17 mm, 21 mm, and 25 mm, respectively.

The relevant design parameters in this section are *a* = 70 mm, *b* = 50 mm, *h* = 0.2 mm, *E* = 7.84 MPa, *v* = 0.47, *ε*_0_ = 8.854 × 10^−3^ pF/mm, *ε_r_*_2_ = 1.00053, *ε_r_*_1_ = 2.5, and the initially parallel gap *g* between the initially flat annular conductive membrane and the insulator layer takes 17 mm, 21 mm, and 25 mm, respectively. When the wind pressure *q* takes different values, the results of numerical calculation of the undetermined constants *b*_0_, *c*_0_, and *d*_0_, maximum membrane deflection *w_m_*, maximum membrane stress *σ_m_*, and capacitance *C* are listed in Table A1 for *g* = 17 mm, in Table A14 for *g* = 21 mm, and in Table A15 for *g* = 25 mm. Figure 13 shows the effect of changing the initially parallel gap *g* on the *C–q* analytical relationships.

It can be seen from Figure 13 that as the initially parallel gap *g* increases, the variation range of the input capacitance *C* decreases while the variation range of the output wind pressure *q* is almost unchanged. The relative increment of the initially parallel gap *g* is (25 mm − 17 mm)/17 mm = 47.06%, and the relative decrement of the input capacitance *C* is equal to (0.504 pF − 2.321 pF)/2.321 pF = −78.29%, which is calculated from Table A1 and Table A13. Therefore, the degree of influence of increasing the initially parallel gap *g* on the variation range of the input capacitance *C* is equal to −78.29%/47.06% = −166.36%; that is, a 1% increase in the initially parallel gap *g* will result in a 1.6636% decrease in the variation range of the input capacitance *C* and will almost not change the variation range of the output wind pressure *q*.

### 4.6. The Experimental Verification of the New Analytical Solution

**Figure 14:**
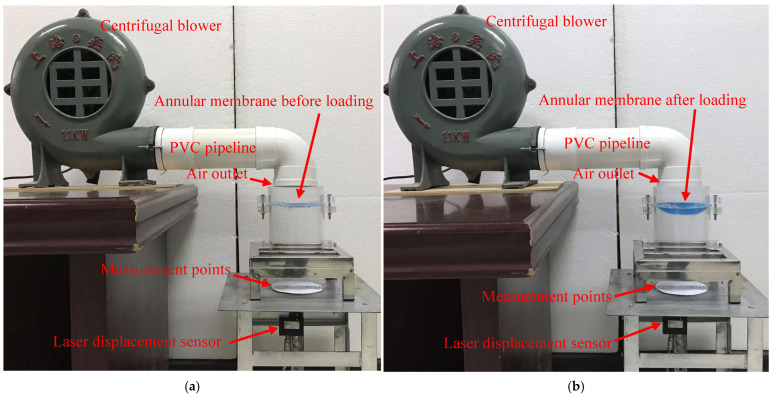

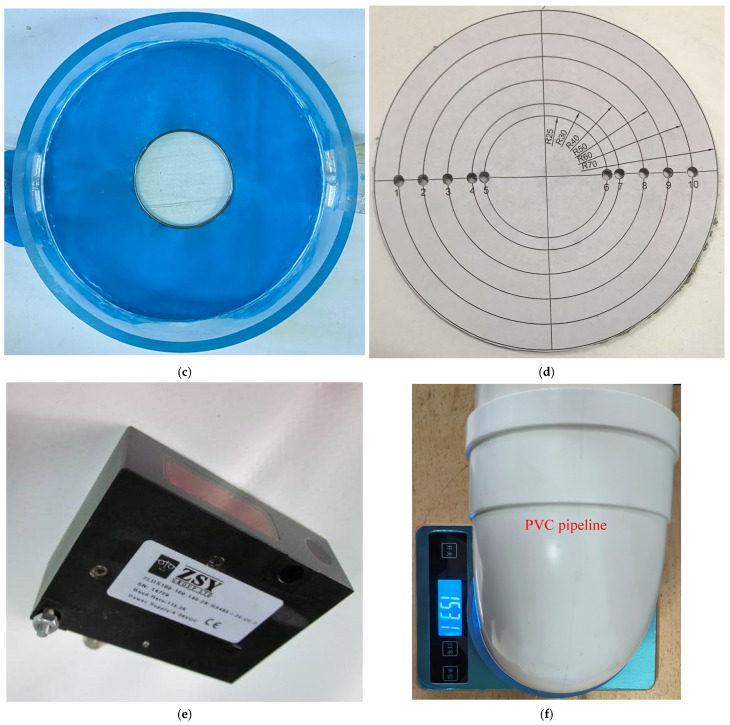
The confirmatory experiment: (**a**) the experimental system before the annular membrane is loaded; (**b**) the experimental system after the annular membrane is loaded; (**c**) a top view of the initially flat annular membrane; (**d**) a top view of the positions of the deflection measurement points; (**e**) a detailed view of the laser displacement sensor; (**f**) the measurement of the total action force of the wind from the air outlet using an electronic scale.

It can be seen from Section 4.4 that the reliability and accuracy of the analytical solution derived in Section 3 are crucial to the numerical design and calibration of the hollow capacitive wind pressure sensor proposed here. To ensure the development quality of the hollow capacitive wind pressure sensor proposed, we carried out experimental verification of the analytical solution derived in Section 3, as shown in Figure 14. An annular membrane (A piece of polymer thin-film) with the outer radius *a* = 70 mm, inner radius *b* = 25 mm, and thickness *h* = 0.3 mm, whose outer edge is firmly clamped by the two round ends of two transparent acrylic hollow cylinders with an inner radius of 70 mm and inner edge is firmly attached to a rigid steel ring with a radius of 25 mm, is initially flat, and after loading by the fast-moving air generated by a centrifugal blower with AC220V and 1.1 KW, it exhibits axisymmetric deformation with large deflection, as shown in Figure 14a (the case before loading) and Figure 14b (the case after loading). Figure 14c is a top view of the initially flat annular membrane. The deflections at points along the diameter of the annular membrane were measured by a laser displacement sensor. Figure 14d is a top view of the positions of the deflection measurement points. Figure 14e is a detailed view of the laser displacement sensor (ZSY Group Ltd., London, UK). The measured results of the deflections *w*(*r*) at *r* = 25 mm, 30 mm, 40 mm, 50 mm, and 60 mm are *w*(25) = 19.397 mm, *w*(30) = 19.143 mm, *w*(40) = 17.283 mm, *w*(50) = 13.314 mm, and *w*(60) = 7.524 mm. The total action force of the fast-moving air from the PVC pipeline was also measured by an electronic scale. The measured result is about 1531 × 10^−3^ kg (15.004 N), as shown in Figure 14f, and after conversion to the wind pressure expressed in transverse uniformly distributed loads, it is *q* = 975.159 Pa.

The Young’s modulus of elasticity *E* and Poisson’s ratio *v* of the polymer thin-film used to fabricate the annular membrane used in the confirmatory experiment in Figure 14 were, before the confirmatory experiment in Figure 14 was carried out, measured by the shaft-loaded blister test technique [57], the measured results are *E* = 0.939645 MPa and *v* = 0.431613. Therefore, with *a* = 70 mm, *b* = 25 mm, *h* = 0.3 mm, *E* = 0.939645 MPa, *v* = 0.431613, and *q* = 975.159 Pa (9.75159 × 10^−4^ MPa), the deflections *w*(*r*) at *r* = 25 mm, 30 mm, 40 mm, 50 mm, and 60 mm can also be calculated by the analytical solution derived in Section 3, the calculated results are *w*(25) = 19.026 mm, *w*(30) = 18.775 mm, *w*(40) = 16.786 mm, *w*(50) = 12.931 mm, and *w*(60) = 7.327 mm.

**Figure 15:**
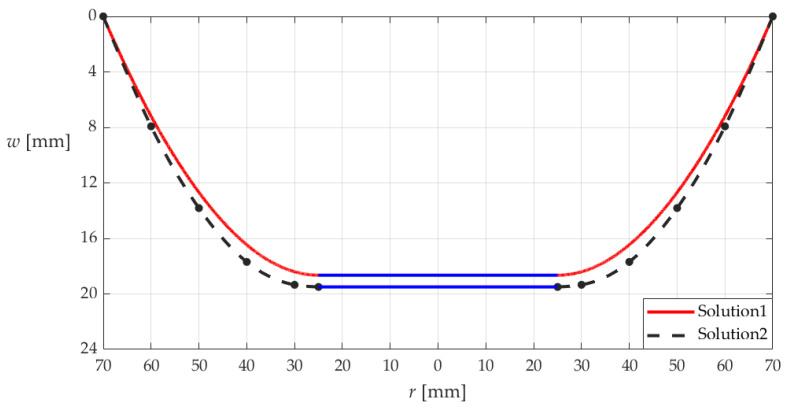
The deflection curves along the diameter of the annular membrane, where “Solution 1” refers to the deflection curve drawn by using the new analytical solution in Section 3, and “Solution 2” refers to the deflection curve drawn by using the experimental measurement data.

The comparison between the measured and calculated results of the deflections *w*(*r*) are shown in Figure 15, where “Solution 1” refers to the deflection curve drawn by using the new analytical solution derived in Section 3, and “Solution 2” refers to the deflection curve drawn by using the experimental measurement data. It can be seen from Figure 15 that the new analytical solution derived in Section 3 is basically reliable; the relative error does not exceed 3%.

## 5. Concluding Remarks

In this paper, a hollow capacitive wind pressure sensor is for the first time proposed, which can be used to detect the pressure exerted on the surface of an object by fast-moving air. It uses a non-parallel plate capacitor as a sensing element, where the movable electrode plate of the capacitor uses a transversely uniformly loaded annular conductive membrane with a fixed outer edge and a rigid inner edge (acting as the wind pressure-sensitive element of the sensor). The new analytical solution of the large deflection elastic behavior of the transversely uniformly loaded annular conductive membrane is derived and compared with the existing analytical solutions in the literature. An example of how to use the new analytical solution to numerically design and calibrate a hollow capacitive wind pressure sensor is given. The effect of changing some important design parameters on the capacitance–pressure (*C*–*q*) analytical relationship of the sensor is investigated comprehensively.

According to the comprehensive investigation in Section 4.5, the effect of changing the design parameters on the *C*–*q* analytical relationships can be summarized as follows.

① A 1% increase in the outer radius *a* will result in a 4.0217% increase in the variation range of the input capacitance *C* and a 5.2078% decrease in the variation range of the output wind pressure *q*.

② A 1% decrease in the inner radius *b* will result in a 1.5015% increase in the variation range of *C* and a 3.682% decrease in the variation range of *q*.

③ A 1% increase in the membrane thickness *h* will result in a 1% increase in the variation range of *q* and will almost not change the variation range of *C*.

④ A 1% decrease in the Young’s modulus of elasticity *E* will result in a 1% decrease in the variation range of *q* and will almost not change the variation range of *C*.

⑤ A 1% decrease in the Poisson’s ratio *v* will result in a 0.0405% increase in the variation range of *C* and a 0.2683% decrease in the variation range of *q*.

⑥ A 1% increase in the insulator layer thickness *t* will result in a 0.9526% increase in the variation range of *C* and will almost not change the variation range of *q*.

⑦ A 1% increase in the initially parallel gap *g* will result in a 1.6636% decrease in the variation range of *C* and will almost not change the variation range of *q*.

Due to the unique hollow configuration of the sensor proposed here, the moving fluids can pass through the hollow area of the sensor. Therefore, the potential applications of the hollow capacitive wind pressure sensor proposed here mainly lie in the following two aspects. It can be used alone to detect the pressure exerted by fast-moving air in the atmosphere or by fast-moving air or gas, etc., in pipelines. In addition, the hollow sensor proposed here can also be used in pairs to form a throttling differential pressure flowmeter to measure the flow rate of fast-moving air or gas, etc., in pipelines (the detail of this flow rate measurement is beyond the scope of this article).

The analytical solution of the large deflection elastic behavior of the transversely uniformly loaded annular membrane, which is newly derived in Section 3, is of great scientific significance to thin-film mechanics. However, the research on the hollow capacitive wind pressure sensor proposed here is still in the theoretical research stage and needs to be further extended to the fabrication and experimental research of well-defined sensors.

## Figures and Tables

**Table 1:** The new and previous membrane governing equations.

	Out-of-Plane Equilibrium Equation	In-Plane Equilibrium Equation	Geometric Equation
New:	$q(r^{2}-b^{2})\sqrt{1+{\left(-\frac{dw}{dr}\right)}^{2}}-2r\sigma_{r}\;h(-\frac{dw}{dr})=0$	$\frac{d}{dr}(\frac{r\sigma_{r}}{\sqrt{1+{\left(-\frac{dw}{dr}\right)}^{2}}})-\sigma_{t}\sqrt{1+{\left(-\frac{dw}{dr}\right)}^{2}}=0$	$\left\{\begin{array}{l} e_{r}=\sqrt{{\left(1+\frac{du}{dr}\right)}^{2}+{\left(-\frac{dw}{dr}\right)}^{2}}\;-1\\ e_{t}=\frac{u}{r} \end{array}\right.$
In [55]:	$q(r^{2}-b^{2})\sqrt{1+{\left(-\frac{dw}{dr}\right)}^{2}}-2r\sigma_{r}\;h(-\frac{dw}{dr})=0$	$\frac{d}{dr}(rh\sigma_{r})-h\sigma_{t}=0$	$\left\{\begin{array}{l} e_{r}=\sqrt{{\left(1+\frac{du}{dr}\right)}^{2}+{\left(-\frac{dw}{dr}\right)}^{2}}\;-1\\ e_{t}=\frac{u}{r} \end{array}\right.$
In [54]:	$q(r^{2}-b^{2})\sqrt{1+{\left(-\frac{dw}{dr}\right)}^{2}}-2r\sigma_{r}\;h(-\frac{dw}{dr})=0$	$\frac{d}{dr}(rh\sigma_{r})-h\sigma_{t}=0$	$\left\{\begin{array}{l} e_{r}=\frac{du}{dr}+\frac{1}{2}(-\frac{dw}{dr}{)}^{2}\\ e_{t}=\frac{u}{r} \end{array}\right.$
In [53]:	$q(r^{2}-b^{2})-2r\sigma_{r}\;h(-\frac{dw}{dr})=0$	$\frac{d}{dr}(rh\sigma_{r})-h\sigma_{t}=0$	$\left\{\begin{array}{l} e_{r}=\frac{du}{dr}+\frac{1}{2}(-\frac{dw}{dr}{)}^{2}\\ e_{t}=\frac{u}{r} \end{array}\right.$

**Table 2:** The analytical expressions of “Function 1” and “Function 2” in Figure 6 and the variation ranges of the output pressure *q* and input capacitance C.

Functions	*C*/pF	*q*/KPa	Analytical Expressions
Function 1	0.392~0.729	0~20	*q* = 64.155*C* − 28.082
Function 2	0.392~2.321	0~54.3296	*q* = −84.636 *C*^10^ + 1.092 × 10^3^ *C*^9^ − 6.110 × 10^3^ *C*^8^ + 1.942 × 10^4^ *C*^7^ − 3.847 × 10^4^ *C*^6^ *+* 4.896 × 10^4^ *C*^5^ − 3.953 × 10^4^ *C*^4^ + 1.893 × 10^4^ *C*^3^ − 4.399 × 10^3^ *C*^2^ *+* 1.605 × 10^2^ *C +* 68.309

## Data Availability

The original contributions presented in this study are included in the article. Further inquiries can be directed to the corresponding author.

## Appendix A

**Table A1:** The numerical calculation results of the undetermined constants *b*_0_, *c*_0_, and *d*_0_, maximum membrane deflection *w_m_*, maximum membrane stress *σ_m_*, and capacitance *C* when the uniformly distributed normal load *q* takes different values, and *a* = 70 mm, *b* = 50 mm, *h* = 0.2 mm, *t* = 0.1 mm, *E* = 7.84 MPa, ν = 0.47, and *g* = 17 mm.

*q*/KPa	*b*_0_	*c*_0_	*d*_0_	*w_m_*/mm	*σ_m_*/MPa	*C*/pF
0	0	0	0	0	0	0.392
0.5	0.019743	0.011214	0.032027	2.992	0.167	0.443
1	0.031424	0.017831	0.040463	3.781	0.263	0.459
2	0.050091	0.028380	0.051207	4.787	0.415	0.483
3	0.065868	0.037270	0.058836	5.503	0.541	0.501
4	0.080043	0.045237	0.064973	6.080	0.652	0.517
6	0.105468	0.059481	0.074821	7.007	0.846	0.546
8	0.128394	0.072275	0.082795	7.759	1.017	0.572
10	0.149658	0.084099	0.089634	8.405	1.170	0.598
12.5	0.174562	0.097895	0.097121	9.115	1.346	0.629
15	0.198069	0.110866	0.103779	9.747	1.506	0.661
20	0.242049	0.135002	0.115417	10.856	1.794	0.729
25	0.283098	0.157374	0.125553	11.826	2.049	0.805
30	0.322015	0.178442	0.134679	12.703	2.278	0.896
35	0.359284	0.198489	0.143077	13.514	2.486	1.008
40	0.395229	0.217699	0.150927	14.274	2.678	1.155
45	0.430078	0.236206	0.158350	14.997	2.854	1.367
50	0.464001	0.254106	0.165433	15.689	3.017	1.715
52.5	0.479838	0.262150	0.168944	16.036	3.108	2.012
53.5	0.486457	0.265606	0.170283	16.168	3.138	2.172
54.3296	0.492803	0.269215	0.171299	16.265	3.150	2.321

**Table A2:** The numerical calculation results of the undetermined constants *b*_0_, *c*_0_, and *d*_0_, maximum membrane deflection *w_m_*, maximum membrane stress *σ_m_*, and capacitance *C* when the uniformly distributed normal load *q* takes different values, and *a* = 75 mm, *b* = 50 mm, *h* = 0.2 mm, *t* = 0.1 mm, *E* = 7.84 MPa, ν = 0.47, and *g* = 17 mm.

*q*/KPa	*b*_0_	*c*_0_	*d*_0_	*w_m_*/mm	*σ_m_*/MPa	*C*/pF
0	0	0	0	0	0	0.510
1	0.036119	0.021189	0.050456	5.055	0.307	0.636
2	0.057596	0.033713	0.063885	6.405	0.483	0.684
3	0.075757	0.044263	0.073435	7.367	0.629	0.724
4	0.092082	0.053713	0.081129	8.144	0.757	0.762
6	0.121382	0.070596	0.093496	9.395	0.980	0.834
8	0.147819	0.085745	0.103534	10.414	1.175	0.907
10	0.172353	0.099732	0.112162	11.293	1.350	0.986
12	0.195495	0.112861	0.119832	12.076	1.511	1.072
14	0.217558	0.125321	0.126802	12.790	1.659	1.172
16	0.238752	0.137236	0.133234	13.450	1.798	1.289
20	0.279081	0.159764	0.144902	14.652	2.051	1.617
25	0.326552	0.186034	0.157890	15.998	2.332	2.539
26.0604	0.336283	0.191385	0.160472	16.265	2.387	2.983

**Table A3:** The numerical calculation results of the undetermined constants *b*_0_, *c*_0_, and *d*_0_, maximum membrane deflection *w_m_*, maximum membrane stress *σ_m_*, and capacitance *C* when the uniformly distributed normal load *q* takes different values, and *a* = 80 mm, *b* = 50 mm, *h* = 0.2 mm, *t* = 0.1 mm, *E* = 7.84 MPa, ν = 0.47, and *g* = 17 mm.

*q*/KPa	*b*_0_	*c*_0_	*d*_0_	*w_m_*/mm	*σ_m_*/MPa	*C*/pF
0	0	0	0	0	0	0.637
1	0.040419	0.024415	0.059873	6.403	0.348	0.851
2	0.064466	0.038832	0.075845	8.119	0.546	0.946
3	0.084810	0.050965	0.087217	9.344	0.710	1.032
4	0.103102	0.061825	0.096391	10.335	0.854	1.119
6	0.135945	0.081206	0.111161	11.935	1.104	1.309
8	0.165594	0.098572	0.123176	13.242	1.320	1.547
10	0.193119	0.114582	0.133527	14.373	1.514	1.882
12	0.219089	0.129590	0.142749	15.384	1.691	2.438
13	0.231606	0.136789	0.147037	15.855	1.774	2.919
13.8975	0.242593	0.143090	0.150727	16.265	1.845	3.655

**Table A4:** The numerical calculation results of the undetermined constants *b*_0_, *c*_0_, and *d*_0_, maximum membrane deflection *w_m_*, maximum membrane stress *σ_m_*, and capacitance *C* when the uniformly distributed normal load *q* takes different values, and *a* = 70 mm, *b* = 45 mm, *h* = 0.2 mm, *t* = 0.1 mm, *E* = 7.84 MPa, ν = 0.47, and *g* = 17 mm.

*q*/KPa	*b*_0_	*c*_0_	*d*_0_	*w_m_*/mm	*σ_m_*/MPa	*C*/pF
0	0	0	0	0	0	0.470
1	0.035917	0.021431	0.053802	5.032	0.308	0.584
2	0.057262	0.034087	0.068115	6.376	0.484	0.627
4	0.091523	0.054277	0.086490	8.107	0.759	0.698
6	0.120616	0.071303	0.099662	9.352	0.982	0.763
8	0.146856	0.086565	0.110350	10.367	1.177	0.828
10	0.171198	0.100643	0.119536	11.241	1.352	0.899
12	0.194152	0.113848	0.127699	12.020	1.513	0.976
14	0.216028	0.126370	0.135117	12.730	1.661	1.064
16	0.237035	0.138335	0.141962	13.388	1.799	1.168
18	0.256855	0.149524	0.148447	14.008	1.930	1.294
20	0.276994	0.160932	0.154377	14.585	2.052	1.455
23	0.304995	0.176540	0.162910	15.407	2.228	1.807
25	0.324004	0.187241	0.168192	15.925	2.332	2.229
26	0.333091	0.192291	0.170784	16.177	2.383	2.558
26.3540	0.336286	0.194064	0.171690	16.265	2.402	2.711

**Table A5:** The numerical calculation results of the undetermined constants *b*_0_, *c*_0_, and *d*_0_, maximum membrane deflection *w_m_*, maximum membrane stress *σ_m_*, and capacitance *C* when the uniformly distributed normal load *q* takes different values, and *a* = 70 mm, *b* = 40 mm, *h* = 0.2 mm, *t* = 0.1 mm, *E* = 7.84 MPa, ν = 0.47, and *g* = 17 mm.

*q*/KPa	*b*_0_	*c*_0_	*d*_0_	*w_m_*/mm	*σ_m_*/MPa	*C*/pF
0	0	0	0	0	0	0.539
1	0.039854	0.025027	0.067629	6.334	0.350	0.715
2	0.063536	0.039767	0.085647	8.030	0.549	0.792
4	0.101542	0.063217	0.108809	10.221	0.858	0.931
6	0.133807	0.082925	0.125445	11.803	1.108	1.081
8	0.162903	0.100540	0.138969	13.095	1.325	1.265
10	0.189889	0.116740	0.150613	14.212	1.518	1.518
11	0.202511	0.124175	0.155891	14.710	1.607	1.689
12	0.215329	0.131892	0.160983	15.212	1.694	1.917
13	0.227277	0.138843	0.165745	15.661	1.776	2.234
14	0.239569	0.146219	0.170424	16.125	1.856	2.763
14.3232	0.243392	0.148468	0.171879	16.265	1.881	3.018

**Table A6:** The numerical calculation results of the undetermined constants *b*_0_, *c*_0_, and *d*_0_, maximum membrane deflection *w_m_*, maximum membrane stress *σ_m_*, and capacitance *C* when the uniformly distributed normal load *q* takes different values, and *a* = 70 mm, *b* = 50 mm, *h* = 0.6 mm, *t* = 0.1 mm, *E* = 7.84 MPa, ν = 0.47, and *g* = 17 mm.

*q*/KPa	*b*_0_	*c*_0_	*d*_0_	*w_m_*/mm	*σ_m_*/MPa	*C*/pF
0	0	0	0	0	0	0.392
1	0.015051	0.008552	0.027947	2.610	0.127	0.436
2	0.023940	0.013593	0.035285	3.296	0.202	0.449
4	0.038124	0.021622	0.044606	4.169	0.318	0.468
6	0.050091	0.028380	0.051206	4.787	0.415	0.483
8	0.060826	0.034431	0.056503	5.284	0.501	0.495
10	0.070737	0.040008	0.061008	5.707	0.579	0.506
30	0.149658	0.084099	0.089634	8.405	1.170	0.598
50	0.213125	0.119148	0.107870	10.136	1.606	0.683
70	0.269683	0.150079	0.122305	11.515	1.967	0.779
90	0.322015	0.178442	0.134679	12.703	2.278	0.896
110	0.371400	0.204978	0.145748	13.772	2.552	1.052
130	0.418572	0.230109	0.155917	14.760	2.797	1.286
150	0.464001	0.254106	0.165433	15.689	3.017	1.715
160	0.486166	0.265739	0.169998	16.137	3.119	2.136
162.9888	0.492803	0.269216	0.171299	16.265	3.150	2.321

**Table A7:** The numerical calculation results of the undetermined constants *b*_0_, *c*_0_, and *d*_0_, maximum membrane deflection *w_m_*, maximum membrane stress *σ_m_*, and capacitance *C* when the uniformly distributed normal load *q* takes different values, and *a* = 70 mm, *b* = 50 mm, *h* = 1 mm, *t* = 0.1 mm, *E* = 7.84 MPa, ν = 0.47, and *g* = 17 mm.

*q*/KPa	*b*_0_	*c*_0_	*d*_0_	*w_m_*/mm	*σ_m_*/MPa	*C*/pF
0	0	0	0	0.000	0.000	0.392
1	0.010697	0.006080	0.023546	2.199	0.091	0.428
2	0.017004	0.009661	0.029712	2.775	0.144	0.439
4	0.027053	0.015357	0.037524	3.506	0.227	0.454
6	0.035517	0.020148	0.043041	4.022	0.297	0.465
8	0.043100	0.024433	0.047457	4.436	0.359	0.474
10	0.050091	0.028380	0.051206	4.787	0.415	0.483
20	0.080043	0.045237	0.064973	6.080	0.652	0.517
30	0.105468	0.059481	0.074821	7.007	0.846	0.546
50	0.149658	0.084098	0.089634	8.405	1.170	0.598
70	0.188811	0.105763	0.101198	9.502	1.443	0.649
100	0.242049	0.135002	0.115417	10.856	1.794	0.729
130	0.291034	0.161681	0.127448	12.008	2.096	0.822
170	0.351945	0.194551	0.141446	13.356	2.446	0.983
210	0.409290	0.225180	0.153942	14.567	2.750	1.230
260	0.477340	0.261113	0.168186	15.959	3.079	1.936
271.6480	0.492803	0.269216	0.171299	16.265	3.150	2.321

**Table A8:** The numerical calculation results of the undetermined constants *b*_0_, *c*_0_, and *d*_0_, maximum membrane deflection *w_m_*, maximum membrane stress *σ_m_*, and capacitance *C* when the uniformly distributed normal load *q* takes different values, and *a* = 70 mm, *b* = 50 mm, *h* = 0.2 mm, *t* = 0.1 mm, *E* = 5 MPa, ν = 0.47, and *g* = 17 mm.

*q*/KPa	*b*_0_	*c*_0_	*d*_0_	*w_m_*/mm	*σ_m_*/MPa	*C*/pF
0	0	0	0	0	0	0.392
1	0.042517	0.024104	0.047132	4.405	0.226	0.473
2	0.067875	0.038398	0.059740	5.588	0.355	0.503
4	0.108708	0.061292	0.075992	7.117	0.555	0.550
6	0.143506	0.080682	0.087702	8.223	0.718	0.590
8	0.174987	0.098129	0.097244	9.126	0.860	0.630
10	0.204266	0.114277	0.105477	9.909	0.987	0.670
15	0.270626	0.150591	0.122879	11.570	1.261	0.784
20	0.332300	0.183987	0.137026	12.929	1.490	0.923
25	0.388743	0.214124	0.149912	14.178	1.694	1.125
30	0.444023	0.243578	0.161276	15.283	1.864	1.485
33	0.474816	0.259525	0.167924	15.936	1.967	1.918
34.6490	0.492803	0.269216	0.171299	16.265	2.009	2.321

**Table A9:** The numerical calculation results of the undetermined constants *b*_0_, *c*_0_, and *d*_0_, maximum membrane deflection *w_m_*, maximum membrane stress *σ_m_*, and capacitance *C* when the uniformly distributed normal load *q* takes different values, and *a* = 70 mm, *b* = 50 mm, *h* = 0.2 mm, *t* = 0.1 mm, *E* = 2.5 MPa, ν = 0.47, and *g* = 17 mm.

*q*/KPa	*b*_0_	*c*_0_	*d*_0_	*w_m_*/mm	*σ_m_*/MPa	*C*/pF
0	0	0	0	0	0	0.392
0.5	0.042517	0.024104	0.047132	4.405	0.113	0.473
1	0.067875	0.038398	0.059740	5.588	0.355	0.503
2	0.108708	0.061292	0.075992	7.117	0.555	0.550
4	0.174987	0.098129	0.097244	9.126	0.860	0.630
6	0.231939	0.129469	0.112822	10.608	0.551	0.712
8	0.283800	0.157755	0.125721	11.842	0.655	0.807
10	0.332300	0.183987	0.137026	12.929	0.745	0.923
12.5	0.388743	0.214124	0.149912	14.178	0.847	1.125
15	0.444023	0.243578	0.161276	15.283	0.932	1.485
16.5	0.474816	0.259525	0.167924	15.936	0.984	1.916
17.3245	0.492803	0.269216	0.171299	16.265	1.004	2.321

**Table A10:** The numerical calculation results of the undetermined constants *b*_0_, *c*_0_, and *d*_0_, maximum membrane deflection *w_m_*, maximum membrane stress *σ_m_*, and capacitance *C* when the uniformly distributed normal load *q* takes different values, and *a* = 70 mm, *b* = 50 mm, *h* = 0.2 mm, *t* = 0.1 mm, *E* = 7.84 MPa, ν = 0.3, and *g* = 17 mm.

*q*/KPa	*b*_0_	*c*_0_	*d*_0_	*w_m_*/mm	*σ_m_*/MPa	*C*/pF
0	0	0	0	0	0	0.392
1	0.029971	0.012548	0.043090	4.004	0.258	0.465
2	0.047823	0.019970	0.054553	5.071	0.407	0.490
4	0.076539	0.031832	0.069262	6.442	0.639	0.529
6	0.100982	0.041856	0.079800	7.427	0.830	0.561
8	0.123076	0.050860	0.088343	8.227	0.997	0.592
10	0.143611	0.059180	0.095677	8.914	1.149	0.622
15	0.189801	0.077769	0.111256	10.377	1.475	0.700
20	0.233293	0.095008	0.123391	11.523	1.763	0.783
25	0.272012	0.110212	0.135037	12.625	2.009	0.884
30	0.311452	0.125575	0.144169	13.492	2.241	1.012
35	0.346041	0.138781	0.154304	14.459	2.442	1.184
40	0.383377	0.153170	0.161764	15.171	2.639	1.451
45	0.415193	0.164903	0.171148	16.073	2.807	1.950
47.1070	0.431885	0.171497	0.173141	16.262	2.884	2.352

**Table A11:** The numerical calculation results of the undetermined constants *b*_0_, *c*_0_, and *d*_0_, maximum membrane deflection *w_m_*, maximum membrane stress *σ_m_*, and capacitance *C* when the uniformly distributed normal load *q* takes different values, and *a* = 70 mm, *b* = 50 mm, *h* = 0.2 mm, *t* = 0.1 mm, *E* = 7.84 MPa, ν = 0.15, and *g* = 17 mm.

*q*/KPa	*b*_0_	*c*_0_	*d*_0_	*w_m_*/mm	*σ_m_*/MPa	*C*/pF
0	0	0	0	0	0	0.392
1	0.029373	0.008294	0.044571	4.120	0.260	0.468
2	0.046905	0.013192	0.056436	5.218	0.410	0.494
4	0.075162	0.021005	0.071669	6.629	0.644	0.535
6	0.099268	0.027597	0.082585	7.642	0.837	0.570
8	0.121099	0.033510	0.091434	8.464	1.006	0.603
10	0.141424	0.038969	0.099031	9.170	1.159	0.636
15	0.186820	0.051064	0.115477	10.700	1.487	0.721
20	0.230552	0.062417	0.127718	11.844	1.785	0.815
25	0.268227	0.072109	0.140458	13.035	2.028	0.932
30	0.308638	0.082360	0.149210	13.856	2.278	1.084
35	0.341737	0.090496	0.160820	14.946	2.470	1.299
40	0.380749	0.100303	0.167416	15.567	2.691	1.674
43	0.397090	0.103956	0.175280	16.109	2.773	2.098
44.4034	0.411176	0.107736	0.174791	16.262	2.854	2.385

**Table A12:** The numerical calculation results of the undetermined constants *b*_0_, *c*_0_, and *d*_0_, maximum membrane deflection *w_m_*, maximum membrane stress *σ_m_*, and capacitance *C* when the uniformly distributed normal load *q* takes different values, and *a* = 70 mm, *b* = 50 mm, *h* = 0.2 mm, *t* = 0.15 mm, *E* = 7.84 MPa, ν = 0.47, and *g* = 17 mm.

*q*/KPa	*b*_0_	*c*_0_	*d*_0_	*w_m_*/mm	*σ_m_*/MPa	*C*/pF
0	0	0	0	0	0	0.587
0.5	0.019743	0.011214	0.032027	2.992	0.167	0.662
1	0.031424	0.017831	0.040463	3.781	0.263	0.688
2	0.050091	0.028380	0.051207	4.787	0.415	0.723
3	0.065868	0.037270	0.058836	5.503	0.541	0.751
4	0.080043	0.045237	0.064973	6.080	0.652	0.774
6	0.105468	0.059481	0.074821	7.007	0.846	0.817
8	0.128394	0.072275	0.082795	7.759	1.017	0.857
10	0.149658	0.084099	0.089634	8.405	1.170	0.895
12.5	0.174562	0.097895	0.097121	9.115	1.346	0.943
15	0.198069	0.110866	0.103779	9.747	1.506	0.992
20	0.242049	0.135002	0.115417	10.856	1.794	1.091
25	0.283098	0.157374	0.125553	11.826	2.049	1.207
30	0.322015	0.178442	0.134679	12.703	2.278	1.340
35	0.359284	0.198489	0.143077	13.514	2.486	1.509
40	0.395229	0.217699	0.150927	14.274	2.678	1.727
45	0.430078	0.236206	0.158350	14.997	2.854	2.034
50	0.464001	0.254106	0.165433	15.689	3.017	2.559
52.5	0.479838	0.262150	0.168944	16.036	3.108	3.010
53.5	0.486457	0.265606	0.170283	16.168	3.138	3.245
54.3296	0.492803	0.269215	0.171299	16.265	3.150	3.457

**Table A13:** The numerical calculation results of the undetermined constants *b*_0_, *c*_0_, and *d*_0_, maximum membrane deflection *w_m_*, maximum membrane stress *σ_m_*, and capacitance *C* when the uniformly distributed normal load *q* takes different values, and *a* = 70 mm, *b* = 50 mm, *h* = 0.2 mm, *t* = 0.3 mm, *E* = 7.84 MPa, ν = 0.47, and *g* = 17 mm.

*q*/KPa	*b*_0_	*c*_0_	*d*_0_	*w_m_*/mm	*σ_m_*/MPa	*C*/pF
0	0	0	0	0	0	1.170
0.5	0.019743	0.011214	0.032027	2.992	0.167	1.316
1	0.031424	0.017831	0.040463	3.781	0.263	1.371
2	0.050091	0.028380	0.051207	4.787	0.415	1.439
3	0.065868	0.037270	0.058836	5.503	0.541	1.495
4	0.080043	0.045237	0.064973	6.080	0.652	1.542
6	0.105468	0.059481	0.074821	7.007	0.846	1.627
8	0.128394	0.072275	0.082795	7.759	1.017	1.705
10	0.149658	0.084099	0.089634	8.405	1.170	1.781
12.5	0.174562	0.097895	0.097121	9.115	1.346	1.877
15	0.198069	0.110866	0.103779	9.747	1.506	1.972
20	0.242049	0.135002	0.115417	10.856	1.794	2.168
25	0.283098	0.157374	0.125553	11.826	2.049	2.392
30	0.322015	0.178442	0.134679	12.703	2.278	2.658
35	0.359284	0.198489	0.143077	13.514	2.486	2.986
40	0.395229	0.217699	0.150927	14.274	2.678	3.418
45	0.430078	0.236206	0.158350	14.997	2.854	4.023
50	0.464001	0.254106	0.165433	15.689	3.017	5.041
52.5	0.479838	0.262150	0.168944	16.036	3.108	5.906
53.5	0.486457	0.265606	0.170283	16.168	3.138	6.365
54.3296	0.492803	0.269215	0.171299	16.265	3.150	6.774

**Table A14:** The numerical calculation results of the undetermined constants *b*_0_, *c*_0_, and *d*_0_, maximum membrane deflection *w_m_*, maximum membrane stress *σ_m_*, and capacitance *C* when the uniformly distributed normal load *q* takes different values, and *a* = 70 mm, *b* = 50 mm, *h* = 0.2 mm, *t* = 0.1 mm, *E* = 7.84 MPa, ν = 0.47, and *g* = 21 mm.

*q*/KPa	*b*_0_	*c*_0_	*d*_0_	*w_m_*/mm	*σ_m_*/MPa	*C*/pF
0	0	0	0	0	0	0.317
1	0.031424	0.017831	0.040463	3.781	0.263	0.360
2	0.050091	0.028380	0.051207	4.787	0.415	0.374
4	0.080043	0.045237	0.064973	6.080	0.652	0.394
6	0.105468	0.059481	0.074821	7.007	0.846	0.409
8	0.128394	0.072275	0.082795	7.759	1.017	0.423
10	0.149658	0.084099	0.089634	8.405	1.170	0.437
20	0.242049	0.135002	0.115417	10.856	1.794	0.497
30	0.322015	0.178442	0.134679	12.703	2.278	0.560
40	0.395229	0.217699	0.150927	14.274	2.678	0.632
50	0.464001	0.254106	0.165433	15.689	3.017	0.723
54.3296	0.492803	0.269215	0.171299	16.265	3.150	0.771

**Table A15:** The numerical calculation results of the undetermined constants *b*_0_, *c*_0_, and *d*_0_, maximum membrane deflection *w_m_*, maximum membrane stress *σ_m_*, and capacitance *C* when the uniformly distributed normal load *q* takes different values, and *a* = 70 mm, *b* = 50 mm, *h* = 0.2 mm, *t* = 0.1 mm, *E* = 7.84 MPa, ν = 0.47, and *g* = 25 mm.

*q*/KPa	*b*_0_	*c*_0_	*d*_0_	*w_m_*/mm	*σ_m_*/MPa	*C*/pF
0	0	0	0	0	0	0.267
1	0.031424	0.017831	0.040463	3.781	0.263	0.296
2	0.050091	0.028380	0.051207	4.787	0.415	0.305
4	0.080043	0.045237	0.064973	6.080	0.652	0.318
6	0.105468	0.059481	0.074821	7.007	0.846	0.328
8	0.128394	0.072275	0.082795	7.759	1.017	0.337
10	0.149658	0.084099	0.089634	8.405	1.170	0.345
20	0.242049	0.135002	0.115417	10.856	1.794	0.380
30	0.322015	0.178442	0.134679	12.703	2.278	0.413
40	0.395229	0.217699	0.150927	14.274	2.678	0.447
50	0.464001	0.254106	0.165433	15.689	3.017	0.486
54.3296	0.492803	0.269215	0.171299	16.265	3.150	0.504

## Appendix B

$$\begin{array}{l} \begin{array}{l} b_{1}=-\frac{Q^{2}\alpha^{2}-Q^{2}\beta^{2}+2b_{0}^{2}-2b_{0}c_{0}}{2\beta b_{0}}, \end{array}\\ \begin{array}{l} b_{2}=-\frac{1}{(Q\alpha^{2}d_{1}-Q\beta^{2}d_{1}-2\beta b_{0}f_{0}-2d_{1}^{2}h_{0})\beta }(-Q\alpha^{2}d_{1}f_{0}h_{2}-Q\alpha^{2}d_{1}f_{1}h_{1}\\ +Q\beta^{2}d_{1}f_{0}h_{2}+Q\beta^{2}d_{1}f_{1}h_{1}+Q\alpha^{2}b_{1}d_{1}-Q\beta^{2}b_{1}d_{1}-2Q\beta d_{1}f_{1}h_{0}+4\beta b_{0}d_{2}^{2}h_{0}\\ +2\beta b_{0}f_{0}^{2}h_{2}+2\beta b_{0}f_{0}f_{1}h_{1}-\beta b_{0}f_{1}^{2}h_{0}-4\beta b_{1}d_{1}d_{2}h_{0}-Qd_{1}f_{0}h_{0}-2\beta b_{0}b_{1}f_{0}\\ -4b_{0}d_{1}d_{2}h_{0}-2b_{1}d_{1}^{2}h_{0}), \end{array}\\ \begin{array}{l} b_{3}=-\frac{1}{(Q\alpha^{2}d_{1}-Q\beta^{2}d_{1}-2\beta b_{0}f_{0}-2d_{1}^{2}h_{0})\beta }(-Q\alpha^{2}d_{1}f_{0}h_{3}-Q\alpha^{2}d_{1}f_{1}h_{2}\\ -Q\alpha^{2}d_{1}f_{2}h_{1}+Q\beta^{2}d_{1}f_{0}h_{3}+Q\beta^{2}d_{1}f_{1}h_{2}+Q\beta^{2}d_{1}f_{2}h_{1}+Q\alpha^{2}b_{2}d_{1}-Q\beta^{2}b_{2}d_{1}\\ -2Q\beta d_{1}f_{2}h_{0}+12\beta b_{0}d_{2}d_{3}h_{0}+2\beta b_{0}f_{0}^{2}h_{3}+2\beta b_{0}f_{0}f_{1}h_{2}+2\beta b_{0}f_{0}f_{2}h_{1}\\ -2\beta b_{0}f_{1}f_{2}h_{0}-6\beta b_{1}d_{1}d_{3}h_{0}-4\beta b_{2}d_{1}d_{2}h_{0}-Qd_{1}f_{1}h_{0}-2\beta b_{0}b_{2}f_{0}-6b_{0}d_{1}d_{3}h_{0}\\ -4b_{1}d_{1}d_{2}h_{0}-2b_{2}d_{1}^{2}h_{0}), \end{array}\\ \begin{array}{l} b_{4}=-\frac{1}{(Q\alpha^{2}d_{1}-Q\beta^{2}d_{1}-2\beta b_{0}f_{0}-2d_{1}^{2}h_{0})\beta }(-Q\alpha^{2}d_{1}f_{0}h_{4}-Q\alpha^{2}d_{1}f_{1}h_{3}\\ -Q\alpha^{2}d_{1}f_{2}h_{2}-Q\alpha^{2}d_{1}f_{3}h_{1}+Q\beta^{2}d_{1}f_{0}h_{4}+Q\beta^{2}d_{1}f_{1}h_{3}+Q\beta^{2}d_{1}f_{2}h_{2}+Q\beta^{2}d_{1}f_{3}h_{1}\\ +Q\alpha^{2}b_{3}d_{1}-Q\beta^{2}b_{3}d_{1}-2Q\beta d_{1}f_{3}h_{0}+16\beta b_{0}d_{2}d_{4}h_{0}+9\beta b_{0}d_{3}^{2}h_{0}+2\beta b_{0}f_{0}^{2}h_{4}\\ +2\beta b_{0}f_{0}f_{1}h_{3}+2\beta b_{0}f_{0}f_{2}h_{2}+2\beta b_{0}f_{0}f_{3}h_{1}-2\beta b_{0}f_{1}f_{3}h_{0}-\beta b_{0}f_{2}^{2}h_{0}-8\beta b_{1}d_{1}d_{4}h_{0}\\ -6\beta b_{2}d_{1}d_{3}h_{0}-4\beta b_{3}d_{1}d_{2}h_{0}-Qd_{1}f_{2}h_{0}-2\beta b_{0}b_{3}f_{0}-8b_{0}d_{1}d_{4}h_{0}-6b_{1}d_{1}d_{3}h_{0}\\ -4b_{2}d_{1}d_{2}h_{0}-2b_{3}d_{1}^{2}h_{0}), \end{array}\\ \begin{array}{l} b_{5}=-\frac{1}{(Q\alpha^{2}d_{1}-Q\beta^{2}d_{1}-2\beta b_{0}f_{0}-2d_{1}^{2}h_{0})\beta }(-Q\alpha^{2}d_{1}f_{0}h_{5}-Q\alpha^{2}d_{1}f_{1}h_{4}\\ -Q\alpha^{2}d_{1}f_{2}h_{3}-Q\alpha^{2}d_{1}f_{3}h_{2}-Q\alpha^{2}d_{1}f_{4}h_{1}+Q\beta^{2}d_{1}f_{0}h_{5}+Q\beta^{2}d_{1}f_{1}h_{4}+Q\beta^{2}d_{1}f_{2}h_{3}\\ +Q\beta^{2}d_{1}f_{3}h_{2}+Q\beta^{2}d_{1}f_{4}h_{1}+Q\alpha^{2}b_{4}d_{1}-Q\beta^{2}b_{4}d_{1}-2Q\beta d_{1}f_{4}h_{0}+20\beta b_{0}d_{2}d_{5}h_{0}\\ +24\beta b_{0}d_{3}d_{4}h_{0}+2\beta b_{0}f_{0}^{2}h_{5}+2\beta b_{0}f_{0}f_{1}h_{4}+2\beta b_{0}f_{0}f_{2}h_{3}+2\beta b_{0}f_{0}f_{3}h_{2}\\ +2\beta b_{0}f_{0}f_{4}h_{1}-2\beta b_{0}f_{1}f_{4}h_{0}-2\beta b_{0}f_{2}f_{3}h_{0}-10\beta b_{1}d_{1}d_{5}h_{0}-8\beta b_{2}d_{1}d_{4}h_{0}-6\beta b_{3}d_{1}d_{3}h_{0}\\ -4\beta b_{4}d_{1}d_{2}h_{0}-Qd_{1}f_{3}h_{0}-2\beta b_{0}b_{4}f_{0}-10b_{0}d_{1}d_{5}h_{0}-8b_{1}d_{1}d_{4}h_{0}-6b_{2}d_{1}d_{3}h_{0}\\ -4b_{3}d_{1}d_{2}h_{0}-2b_{4}d_{1}^{2}h_{0}), \end{array}\\ \begin{array}{l} b_{6}=-\frac{1}{(Q\alpha^{2}d_{1}-Q\beta^{2}d_{1}-2\beta b_{0}f_{0}-2d_{1}^{2}h_{0})\beta }(-Q\alpha^{2}d_{1}f_{0}h_{6}-Q\alpha^{2}d_{1}f_{1}h_{5}-Q\alpha^{2}d_{1}f_{2}h_{4}\\ -Q\alpha^{2}d_{1}f_{3}h_{3}-Q\alpha^{2}d_{1}f_{4}h_{2}-Q\alpha^{2}d_{1}f_{5}h_{1}+Q\beta^{2}d_{1}f_{0}h_{6}+Q\beta^{2}d_{1}f_{1}h_{5}+Q\beta^{2}d_{1}f_{2}h_{4}\\ +Q\beta^{2}d_{1}f_{3}h_{3}+Q\beta^{2}d_{1}f_{4}h_{2}+Q\beta^{2}d_{1}f_{5}h_{1}+Q\alpha^{2}b_{5}d_{1}-Q\beta^{2}b_{5}d_{1}-2Q\beta d_{1}f_{5}h_{0}\\ +24\beta b_{0}d_{2}d_{6}h_{0}+30\beta b_{0}d_{3}d_{5}h_{0}+16\beta b_{0}d_{4}^{2}h_{0}+2\beta b_{0}f_{0}^{2}h_{6}+2\beta b_{0}f_{0}f_{1}h_{5}+2\beta b_{0}f_{0}f_{2}h_{4}\\ +2\beta b_{0}f_{0}f_{3}h_{3}+2\beta b_{0}f_{0}f_{4}h_{2}+2\beta b_{0}f_{0}f_{5}h_{1}-2\beta b_{0}f_{1}f_{5}h_{0}-2\beta b_{0}f_{2}f_{4}h_{0}-\beta b_{0}f_{3}^{2}h_{0}\\ -12\beta b_{1}d_{1}d_{6}h_{0}-10\beta b_{2}d_{1}d_{5}h_{0}-8\beta b_{3}d_{1}d_{4}h_{0}-6\beta b_{4}d_{1}d_{3}h_{0}-4\beta b_{5}d_{1}d_{2}h_{0}-Qd_{1}f_{4}h_{0}\\ -2\beta b_{0}b_{5}f_{0}-12b_{0}d_{1}d_{6}h_{0}-10b_{1}d_{1}d_{5}h_{0}-8b_{2}d_{1}d_{4}h_{0}-6b_{3}d_{1}d_{3}h_{0}-4b_{4}d_{1}d_{2}h_{0}-2b_{5}d_{1}^{2}h_{0}), \end{array}\\ \begin{array}{l} b_{7}=-\frac{1}{(Q\alpha^{2}d_{1}-Q\beta^{2}d_{1}-2\beta b_{0}f_{0}-2d_{1}^{2}h_{0})\beta }(Q\alpha^{2}b_{6}d_{1}-Q\beta^{2}b_{6}d_{1}+2\beta b_{0}f_{0}^{2}h_{7}\\ -2\beta b_{0}b_{6}f_{0}-d_{1}h_{0}Qf_{5}-14d_{1}h_{0}b_{0}d_{7}-12d_{1}h_{0}b_{1}d_{6}-10d_{1}h_{0}b_{2}d_{5}-8d_{1}h_{0}b_{3}d_{4}\\ -6d_{1}h_{0}b_{4}d_{3}-4d_{1}h_{0}b_{5}d_{2}-2b_{6}d_{1}^{2}h_{0}+40\beta b_{0}d_{4}d_{5}h_{0}-Q\alpha^{2}d_{1}f_{1}h_{6}+Q\beta^{2}d_{1}f_{1}h_{6}\\ -Q\alpha^{2}d_{1}f_{2}h_{5}+Q\beta^{2}d_{1}f_{2}h_{5}-Q\alpha^{2}d_{1}f_{3}h_{4}+Q\beta^{2}d_{1}f_{3}h_{4}-Q\alpha^{2}d_{1}f_{4}h_{3}+Q\beta^{2}d_{1}f_{4}h_{3}\\ -Q\alpha^{2}d_{1}f_{5}h_{2}+Q\beta^{2}d_{1}f_{5}h_{2}-Q\alpha^{2}d_{1}f_{6}h_{1}+Q\beta^{2}d_{1}f_{6}h_{1}-2\beta b_{0}f_{1}f_{6}h_{0}-2\beta b_{0}f_{2}f_{5}h_{0}\\ -2\beta b_{0}f_{3}f_{4}h_{0}-2d_{1}h_{0}Q\beta f_{6}-14d_{1}h_{0}\beta b_{1}d_{7}-12d_{1}h_{0}\beta b_{2}d_{6}-10d_{1}h_{0}\beta b_{3}d_{5}\\ -8d_{1}h_{0}\beta b_{4}d_{4}-6d_{1}h_{0}\beta b_{5}d_{3}-4d_{1}h_{0}\beta b_{6}d_{2}+28\beta b_{0}d_{2}d_{7}h_{0}+36\beta b_{0}d_{3}d_{6}h_{0}\\ -Q\alpha^{2}d_{1}f_{0}h_{7}+Q\beta^{2}d_{1}f_{0}h_{7}+2\beta b_{0}f_{0}f_{1}h_{6}+2\beta b_{0}f_{0}f_{2}h_{5}+2\beta b_{0}f_{0}f_{3}h_{4}\\ +2\beta b_{0}f_{0}f_{4}h_{3}+2\beta b_{0}f_{0}f_{5}h_{2}+2\beta b_{0}f_{0}f_{6}h_{1}), \end{array}\\ \begin{array}{l} b_{8}=-\frac{1}{(Q\alpha^{2}d_{1}-Q\beta^{2}d_{1}-2\beta b_{0}f_{0}-2d_{1}^{2}h_{0})\beta }(2\beta b_{0}f_{0}f_{4}h_{4}+2\beta b_{0}f_{0}f_{5}h_{3}\\ +2\beta b_{0}f_{0}f_{6}h_{2}+2\beta b_{0}f_{0}f_{7}h_{1}-Q\alpha^{2}d_{1}f_{0}h_{8}+Q\beta^{2}d_{1}f_{0}h_{8}+2\beta b_{0}f_{0}f_{1}h_{7}-2d_{1}h_{0}Q\beta f_{7}\\ -16d_{1}h_{0}\beta b_{1}d_{8}-14d_{1}h_{0}\beta b_{2}d_{7}-12d_{1}h_{0}\beta b_{3}d_{6}-10d_{1}h_{0}\beta b_{4}d_{5}-8d_{1}h_{0}\beta b_{5}d_{4}\\ -6d_{1}h_{0}\beta b_{6}d_{3}-4d_{1}h_{0}\beta b_{7}d_{2}+32\beta b_{0}d_{2}d_{8}h_{0}+42\beta b_{0}d_{3}d_{7}h_{0}+48\beta b_{0}d_{4}d_{6}h_{0}\\ -Q\alpha^{2}d_{1}f_{1}h_{7}+Q\beta^{2}d_{1}f_{1}h_{7}-Q\alpha^{2}d_{1}f_{2}h_{6}+Q\beta^{2}d_{1}f_{2}h_{6}-Q\alpha^{2}d_{1}f_{3}h_{5}+Q\beta^{2}d_{1}f_{3}h_{5}\\ -Q\alpha^{2}d_{1}f_{4}h_{4}+Q\beta^{2}d_{1}f_{4}h_{4}-Q\alpha^{2}d_{1}f_{5}h_{3}+Q\beta^{2}d_{1}f_{5}h_{3}-Q\alpha^{2}d_{1}f_{6}h_{2}+Q\beta^{2}d_{1}f_{6}h_{2}\\ -Q\alpha^{2}d_{1}f_{7}h_{1}+Q\beta^{2}d_{1}f_{7}h_{1}-2\beta b_{0}f_{1}f_{7}h_{0}-2\beta b_{0}f_{2}f_{6}h_{0}-2\beta b_{0}f_{3}f_{5}h_{0}+2\beta b_{0}f_{0}f_{2}h_{6}\\ +2\beta b_{0}f_{0}f_{3}h_{5}-2b_{7}d_{1}^{2}h_{0}-\beta b_{0}f_{4}^{2}h_{0}+Q\alpha^{2}b_{7}d_{1}-Q\beta^{2}b_{7}d_{1}-d_{1}h_{0}Qf_{6}-16d_{1}h_{0}b_{0}d_{8}\\ -14d_{1}h_{0}b_{1}d_{7}-12d_{1}h_{0}b_{2}d_{6}-10d_{1}h_{0}b_{3}d_{5}-8d_{1}h_{0}b_{4}d_{4}-6d_{1}h_{0}b_{5}d_{3}-4d_{1}h_{0}b_{6}d_{2}\\ +25\beta b_{0}d_{5}^{2}h_{0}+2\beta b_{0}f_{0}^{2}h_{8}-2\beta b_{0}b_{7}f_{0}), \end{array}\\ \begin{array}{l} b_{9}=-\frac{1}{(Q\alpha^{2}d_{1}-Q\beta^{2}d_{1}-2\beta b_{0}f_{0}-2d_{1}^{2}h_{0})\beta }(-12d_{1}h_{0}b_{3}d_{6}-10d_{1}h_{0}b_{4}d_{5}-8d_{1}h_{0}b_{5}d_{4}\\ -6d_{1}h_{0}b_{6}d_{3}-4d_{1}h_{0}b_{7}d_{2}+2\beta b_{0}f_{0}^{2}h_{9}-2\beta b_{0}b_{8}f_{0}-Q\alpha^{2}d_{1}f_{3}h_{6}+Q\beta^{2}d_{1}f_{3}h_{6}\\ +Q\beta^{2}d_{1}f_{2}h_{7}+60\beta b_{0}d_{5}d_{6}h_{0}-Q\alpha^{2}d_{1}f_{1}h_{8}+Q\beta^{2}d_{1}f_{1}h_{8}-Q\alpha^{2}d_{1}f_{2}h_{7}-4d_{1}h_{0}\beta b_{8}d_{2}\\ +36\beta b_{0}d_{2}d_{9}h_{0}+48\beta b_{0}d_{3}d_{8}h_{0}+56\beta b_{0}d_{4}d_{7}h_{0}-Q\alpha^{2}d_{1}f_{4}h_{5}+Q\beta^{2}d_{1}f_{4}h_{5}-Q\alpha^{2}d_{1}f_{5}h_{4}\\ +Q\beta^{2}d_{1}f_{5}h_{4}-Q\alpha^{2}d_{1}f_{6}h_{3}+Q\beta^{2}d_{1}f_{6}h_{3}-Q\alpha^{2}d_{1}f_{7}h_{2}+Q\beta^{2}d_{1}f_{7}h_{2}-Q\alpha^{2}d_{1}f_{8}h_{1}\\ +Q\beta^{2}d_{1}f_{8}h_{1}-2\beta b_{0}f_{1}f_{8}h_{0}-2\beta b_{0}f_{2}f_{7}h_{0}-2\beta b_{0}f_{3}f_{6}h_{0}-2\beta b_{0}f_{4}f_{5}h_{0}-2d_{1}h_{0}Q\beta f_{8}\\ -18d_{1}h_{0}\beta b_{1}d_{9}-16d_{1}h_{0}\beta b_{2}d_{8}-14d_{1}h_{0}\beta b_{3}d_{7}-12d_{1}h_{0}\beta b_{4}d_{6}-10d_{1}h_{0}\beta b_{5}d_{5}\\ -8d_{1}h_{0}\beta b_{6}d_{4}-6d_{1}h_{0}\beta b_{7}d_{3}+2\beta b_{0}f_{0}f_{8}h_{1}-Q\alpha^{2}d_{1}f_{0}h_{9}+Q\beta^{2}d_{1}f_{0}h_{9}+2\beta b_{0}f_{0}f_{1}h_{8}\\ +2\beta b_{0}f_{0}f_{2}h_{7}+2\beta b_{0}f_{0}f_{3}h_{6}+2\beta b_{0}f_{0}f_{4}h_{5}+2\beta b_{0}f_{0}f_{5}h_{4}+2\beta b_{0}f_{0}f_{6}h_{3}+2\beta b_{0}f_{0}f_{7}h_{2}\\ -2b_{8}d_{1}^{2}h_{0}+Q\alpha^{2}b_{8}d_{1}-Q\beta^{2}b_{8}d_{1}-d_{1}h_{0}Qf_{7}-18d_{1}h_{0}b_{0}d_{9}-16d_{1}h_{0}b_{1}d_{8}-14d_{1}h_{0}b_{2}d_{7}), \end{array}\\ \begin{array}{l} b_{10}=-\frac{1}{(Q\alpha^{2}d_{1}-Q\beta^{2}d_{1}-2\beta b_{0}f_{0}-2d_{1}^{2}h_{0})\beta }(-2b_{9}d_{1}^{2}h_{0}+2\beta b_{0}f_{0}f_{3}h_{7}\\ +2\beta b_{0}f_{0}f_{4}h_{6}+2\beta b_{0}f_{0}f_{5}h_{5}+2\beta b_{0}f_{0}f_{6}h_{4}+2\beta b_{0}f_{0}f_{7}h_{3}+2\beta b_{0}f_{0}f_{8}h_{2}\\ +2\beta b_{0}f_{0}f_{9}h_{1}-Q\alpha^{2}d_{1}f_{0}h_{10}-12d_{1}h_{0}\beta b_{5}d_{6}-10d_{1}h_{0}\beta b_{6}d_{5}-8d_{1}h_{0}\beta b_{7}d_{4}\\ -6d_{1}h_{0}\beta b_{8}d_{3}-4d_{1}h_{0}\beta b_{9}d_{2}-Q\alpha^{2}d_{1}f_{1}h_{9}+Q\beta^{2}d_{1}f_{1}h_{9}-Q\alpha^{2}d_{1}f_{2}h_{8}+Q\beta^{2}d_{1}f_{2}h_{8}\\ -Q\alpha^{2}d_{1}f_{3}h_{7}+Q\beta^{2}d_{1}f_{3}h_{7}-Q\alpha^{2}d_{1}f_{4}h_{6}+Q\beta^{2}d_{1}f_{4}h_{6}-Q\alpha^{2}d_{1}f_{5}h_{5}+Q\beta^{2}d_{1}f_{5}h_{5}\\ -Q\alpha^{2}d_{1}f_{6}h_{4}+Q\beta^{2}d_{1}f_{6}h_{4}-Q\alpha^{2}d_{1}f_{7}h_{3}+Q\beta^{2}d_{1}f_{7}h_{3}-Q\alpha^{2}d_{1}f_{8}h_{2}+Q\beta^{2}d_{1}f_{8}h_{2}\\ -Q\alpha^{2}d_{1}f_{9}h_{1}+Q\beta^{2}d_{1}f_{9}h_{1}+40\beta b_{0}d_{2}d_{10}h_{0}+54\beta b_{0}d_{3}d_{9}h_{0}+64\beta b_{0}d_{4}d_{8}h_{0}\\ +70\beta b_{0}d_{5}d_{7}h_{0}-2\beta b_{0}f_{1}f_{9}h_{0}-2\beta b_{0}f_{2}f_{8}h_{0}-2\beta b_{0}f_{3}f_{7}h_{0}-2\beta b_{0}f_{4}f_{6}h_{0}\\ -2d_{1}h_{0}Q\beta f_{9}-20d_{1}h_{0}\beta b_{1}d_{10}-18d_{1}h_{0}\beta b_{2}d_{9}-16d_{1}h_{0}\beta b_{3}d_{8}-14d_{1}h_{0}\beta b_{4}d_{7}\\ +Q\beta^{2}d_{1}f_{0}h_{10}+2\beta b_{0}f_{0}f_{1}h_{9}+2\beta b_{0}f_{0}f_{2}h_{8}+Q\alpha^{2}b_{9}d_{1}-Q\beta^{2}b_{9}d_{1}+36\beta b_{0}d_{6}^{2}h_{0}\\ -\beta b_{0}f_{5}^{2}h_{0}-4d_{1}h_{0}b_{8}d_{2}-d_{1}h_{0}Qf_{8}-20d_{1}h_{0}b_{0}d_{10}-18d_{1}h_{0}b_{1}d_{9}-16d_{1}h_{0}b_{2}d_{8}\\ -14d_{1}h_{0}b_{3}d_{7}-12d_{1}h_{0}b_{4}d_{6}-10d_{1}h_{0}b_{5}d_{5}-8d_{1}h_{0}b_{6}d_{4}-6d_{1}h_{0}b_{7}d_{3}+2\beta b_{0}f_{0}^{2}h_{10}\\ -2\beta b_{0}b_{9}f_{0}), \end{array}\\ \begin{array}{l} b_{11}=-\frac{1}{(Q\alpha^{2}d_{1}-Q\beta^{2}d_{1}-2\beta b_{0}f_{0}-2d_{1}^{2}h_{0})\beta }(-10d_{1}h_{0}\beta b_{7}d_{5}-8d_{1}h_{0}\beta b_{8}d_{4}\\ -6d_{1}h_{0}\beta b_{9}d_{3}-4d_{1}h_{0}\beta b_{10}d_{2}-Q\alpha^{2}d_{1}f_{1}h_{10}+Q\beta^{2}d_{1}f_{1}h_{10}-Q\alpha^{2}d_{1}f_{2}h_{9}\\ +Q\beta^{2}d_{1}f_{2}h_{9}-Q\alpha^{2}d_{1}f_{3}h_{8}+Q\beta^{2}d_{1}f_{3}h_{8}-Q\alpha^{2}d_{1}f_{4}h_{7}+Q\beta^{2}d_{1}f_{4}h_{7}-Q\alpha^{2}d_{1}f_{5}h_{6}\\ +Q\beta^{2}d_{1}f_{5}h_{6}-Q\alpha^{2}d_{1}f_{6}h_{5}+Q\beta^{2}d_{1}f_{6}h_{5}-Q\alpha^{2}d_{1}f_{7}h_{4}+Q\beta^{2}d_{1}f_{7}h_{4}-Q\alpha^{2}d_{1}f_{8}h_{3}\\ +Q\beta^{2}d_{1}f_{8}h_{3}-Q\alpha^{2}d_{1}f_{9}h_{2}+Q\beta^{2}d_{1}f_{9}h_{2}-Q\alpha^{2}d_{1}f_{10}h_{1}+Q\beta^{2}d_{1}f_{10}h_{1}+44\beta b_{0}d_{2}d_{11}h_{0}\\ +60\beta b_{0}d_{3}d_{10}h_{0}+72\beta b_{0}d_{4}d_{9}h_{0}+80\beta b_{0}d_{5}d_{8}h_{0}+84\beta b_{0}d_{6}d_{7}h_{0}-2\beta b_{0}f_{1}f_{10}h_{0}\\ -2\beta b_{0}f_{2}f_{9}h_{0}-2\beta b_{0}f_{3}f_{8}h_{0}-2\beta b_{0}f_{4}f_{7}h_{0}-2\beta b_{0}f_{5}f_{6}h_{0}-Q\alpha^{2}d_{1}f_{0}h_{11}+Q\beta^{2}d_{1}f_{0}h_{11}\\ +2\beta b_{0}f_{0}f_{1}h_{10}+2\beta b_{0}f_{0}f_{2}h_{9}+2\beta b_{0}f_{0}f_{3}h_{8}-2d_{1}h_{0}Q\beta f_{10}-22d_{1}h_{0}\beta b_{1}d_{11}\\ -20d_{1}h_{0}\beta b_{2}d_{10}-18d_{1}h_{0}\beta b_{3}d_{9}-2b_{10}d_{1}^{2}h_{0}-d_{1}h_{0}Qf_{9}-22d_{1}h_{0}b_{0}d_{11}-20d_{1}h_{0}b_{1}d_{10}\\ -18d_{1}h_{0}b_{2}d_{9}-16d_{1}h_{0}b_{3}d_{8}-14d_{1}h_{0}b_{4}d_{7}-12d_{1}h_{0}b_{5}d_{6}-10d_{1}h_{0}b_{6}d_{5}-8d_{1}h_{0}b_{7}d_{4}\\ -6d_{1}h_{0}b_{8}d_{3}-4d_{1}h_{0}b_{9}d_{2}+2\beta b_{0}f_{0}^{2}h_{11}-2\beta b_{0}b_{10}f_{0}+Q\alpha^{2}b_{10}d_{1}-Q\beta^{2}b_{10}d_{1}\\ -16d_{1}h_{0}\beta b_{4}d_{8}-14d_{1}h_{0}\beta b_{5}d_{7}-12d_{1}h_{0}\beta b_{6}d_{6}+2\beta b_{0}f_{0}f_{4}h_{7}+2\beta b_{0}f_{0}f_{5}h_{6}\\ +2\beta b_{0}f_{0}f_{6}h_{5}+2\beta b_{0}f_{0}f_{7}h_{4}+2\beta b_{0}f_{0}f_{8}h_{3}+2\beta b_{0}f_{0}f_{9}h_{2}+2\beta b_{0}f_{0}f_{10}h_{1}), \end{array}\\ \begin{array}{l} b_{12}=-\frac{1}{(Q\alpha^{2}d_{1}-Q\beta^{2}d_{1}-2\beta b_{0}f_{0}-2d_{1}^{2}h_{0})\beta }(2\beta b_{0}f_{0}f_{10}h_{2}-Q\alpha^{2}d_{1}f_{1}h_{11}\\ +Q\beta^{2}d_{1}f_{1}h_{11}-Q\alpha^{2}d_{1}f_{2}h_{10}+Q\beta^{2}d_{1}f_{2}h_{10}-Q\alpha^{2}d_{1}f_{3}h_{9}+Q\beta^{2}d_{1}f_{3}h_{9}-Q\alpha^{2}d_{1}f_{4}h_{8}\\ +Q\beta^{2}d_{1}f_{4}h_{8}-Q\alpha^{2}d_{1}f_{5}h_{7}+Q\beta^{2}d_{1}f_{5}h_{7}-Q\alpha^{2}d_{1}f_{6}h_{6}+Q\beta^{2}d_{1}f_{6}h_{6}-Q\alpha^{2}d_{1}f_{7}h_{5}\\ +Q\beta^{2}d_{1}f_{7}h_{5}-Q\alpha^{2}d_{1}f_{8}h_{4}+Q\beta^{2}d_{1}f_{8}h_{4}-Q\alpha^{2}d_{1}f_{9}h_{3}+Q\beta^{2}d_{1}f_{9}h_{3}-Q\alpha^{2}d_{1}f_{10}h_{2}\\ +Q\beta^{2}d_{1}f_{10}h_{2}-Q\alpha^{2}d_{1}f_{11}h_{1}+Q\beta^{2}d_{1}f_{11}h_{1}+48\beta b_{0}d_{2}d_{12}h_{0}+66\beta b_{0}d_{3}d_{11}h_{0}\\ +80\beta b_{0}d_{4}d_{10}h_{0}+90\beta b_{0}d_{5}d_{9}h_{0}+96\beta b_{0}d_{6}d_{8}h_{0}-2\beta b_{0}f_{1}f_{11}h_{0}-2\beta b_{0}f_{2}f_{10}h_{0}\\ -2\beta b_{0}f_{3}f_{9}h_{0}-2\beta b_{0}f_{4}f_{8}h_{0}-2\beta b_{0}f_{5}f_{7}h_{0}-2d_{1}h_{0}Q\beta f_{11}-24d_{1}h_{0}\beta b_{1}d_{12}\\ -22d_{1}h_{0}\beta b_{2}d_{11}-20d_{1}h_{0}\beta b_{3}d_{10}-18d_{1}h_{0}\beta b_{4}d_{9}-16d_{1}h_{0}\beta b_{5}d_{8}-14d_{1}h_{0}\beta b_{6}d_{7}\\ -12d_{1}h_{0}\beta b_{7}d_{6}-10d_{1}h_{0}\beta b_{8}d_{5}-8d_{1}h_{0}\beta b_{9}d_{4}-6d_{1}h_{0}\beta b_{10}d_{3}-4d_{1}h_{0}\beta b_{11}d_{2}\\ +2\beta b_{0}f_{0}f_{11}h_{1}-Q\alpha^{2}d_{1}f_{0}h_{12}+Q\beta^{2}d_{1}f_{0}h_{12}+2\beta b_{0}f_{0}f_{1}h_{11}+2\beta b_{0}f_{0}f_{2}h_{10}\\ +2\beta b_{0}f_{0}f_{3}h_{9}+2\beta b_{0}f_{0}f_{4}h_{8}+2\beta b_{0}f_{0}f_{5}h_{7}+2\beta b_{0}f_{0}f_{6}h_{6}+2\beta b_{0}f_{0}f_{7}h_{5}\\ +2\beta b_{0}f_{0}f_{8}h_{4}+2\beta b_{0}f_{0}f_{9}h_{3}-2b_{11}d_{1}^{2}h_{0}+Q\alpha^{2}b_{11}d_{1}-Q\beta^{2}b_{11}d_{1}+49\beta b_{0}d_{7}^{2}h_{0}\\ -\beta b_{0}f_{6}^{2}h_{0}-22d_{1}h_{0}b_{1}d_{11}-20d_{1}h_{0}b_{2}d_{10}-d_{1}h_{0}Qf_{10}-24d_{1}h_{0}b_{0}d_{12}-4d_{1}h_{0}b_{10}d_{2}\\ -6d_{1}h_{0}b_{9}d_{3}-14d_{1}h_{0}b_{5}d_{7}-12d_{1}h_{0}b_{6}d_{6}-10d_{1}h_{0}b_{7}d_{5}-8d_{1}h_{0}b_{8}d_{4}-18d_{1}h_{0}b_{3}d_{9}\\ -16d_{1}h_{0}b_{4}d_{8}+2\beta b_{0}f_{0}^{2}h_{12}-2\beta b_{0}b_{11}f_{0}), \end{array}\\ c_{1}=\frac{\nu \beta b_{1}+\nu b_{0}-c_{0}-1+\sqrt{\nu^{2}c_{0}^{2}-2\nu b_{0}c_{0}-2\nu c_{0}+b_{0}^{2}-d_{1}^{2}+2b_{0}+1}}{\beta },\\ \begin{array}{l} c_{2}=\frac{1}{2\beta (\nu \beta b_{1}-\beta c_{1}+\nu b_{0}-c_{0}-1)}(2\beta^{2}\nu^{2}b_{1}b_{2}-2\beta^{2}\nu b_{2}c_{1}+2\beta \nu^{2}b_{0}b_{2}+2\beta \nu^{2}b_{1}^{2}\\ -4\beta \nu b_{1}c_{1}-2\beta \nu b_{2}c_{0}+2\nu^{2}b_{0}b_{1}-\nu^{2}c_{0}c_{1}-2\nu \beta b_{2}+2\beta c_{1}^{2}-\nu b_{0}c_{1}-\nu b_{1}c_{0}-2\nu b_{1}\\ +\nu c_{1}-b_{0}b_{1}+2c_{0}c_{1}+2d_{1}d_{2}-b_{1}+2c_{1}), \end{array}\\ \begin{array}{l} c_{3}=\frac{1}{6\beta (\nu \beta b_{1}-\beta c_{1}+\nu b_{0}-c_{0}-1)}6\beta^{2}\nu^{2}b_{1}b_{3}+4\beta^{2}\nu^{2}b_{2}^{2}-8\beta^{2}\nu b_{2}c_{2}-6\beta^{2}\nu b_{3}c_{1}\\ +6\beta \nu^{2}b_{0}b_{3}+14\beta \nu^{2}b_{1}b_{2}+4\beta^{2}c_{2}^{2}-14\beta \nu b_{1}c_{2}-14\beta \nu b_{2}c_{1}-6\beta \nu b_{3}c_{0}+6\nu^{2}b_{0}b_{2}\\ +4\nu^{2}b_{1}^{2}-2\nu^{2}c_{0}c_{2}-\nu^{2}c_{1}^{2}-6\nu \beta b_{3}+14\beta c_{1}c_{2}-4\nu b_{0}c_{2}-6\nu b_{1}c_{1}-4\nu b_{2}c_{0}-6\nu b_{2}+2\nu c_{2}\\ -2b_{0}b_{2}-b_{1}^{2}+6c_{0}c_{2}+4c_{1}^{2}+6d_{1}d_{3}+4d_{2}^{2}-2b_{2}+6c_{2}), \end{array}\\ \begin{array}{l} c_{4}=\frac{1}{4\beta (\nu \beta b_{1}-\beta c_{1}+\nu b_{0}-c_{0}-1)}(4\beta^{2}\nu^{2}b_{1}b_{4}+6\beta^{2}\nu^{2}b_{2}b_{3}-6\beta^{2}\nu b_{2}c_{3}-6\beta^{2}\nu b_{3}c_{2}\\ -4\beta^{2}\nu b_{4}c_{1}+4\beta \nu^{2}b_{0}b_{4}+10\beta \nu^{2}b_{1}b_{3}+6\beta \nu^{2}b_{2}^{2}+6\beta^{2}c_{2}c_{3}-10\beta \nu b_{1}c_{3}-12\beta \nu b_{2}c_{2}\\ -10\beta \nu b_{3}c_{1}-4\beta \nu b_{4}c_{0}+4\nu^{2}b_{0}b_{3}+6\nu^{2}b_{1}b_{2}-\nu^{2}c_{0}c_{3}-\nu^{2}c_{1}c_{2}-4\nu \beta b_{4}+10\beta c_{1}c_{3}\\ +6\beta c_{2}^{2}-3\nu b_{0}c_{3}-5\nu b_{1}c_{2}-5\nu b_{2}c_{1}-3\nu b_{3}c_{0}-4\nu b_{3}+\nu c_{3}-b_{0}b_{3}-b_{1}b_{2}+4c_{0}c_{3}+6c_{1}c_{2}\\ +4d_{1}d_{4}+6d_{2}d_{3}-b_{3}+4c_{3}), \end{array}\\ \begin{array}{l} c_{5}=\frac{1}{10\beta (\nu \beta b_{1}-\beta c_{1}+\nu b_{0}-c_{0}-1)}(10\beta^{2}\nu^{2}b_{1}b_{5}+16\beta^{2}\nu^{2}b_{2}b_{4}+9\beta^{2}\nu^{2}b_{3}^{2}\\ -16\beta^{2}\nu b_{2}c_{4}-18\beta^{2}\nu b_{3}c_{3}-16\beta^{2}\nu b_{4}c_{2}-10\beta^{2}\nu b_{5}c_{1}+10\beta \nu^{2}b_{0}b_{5}+26\beta \nu^{2}b_{1}b_{4}\\ +34\beta \nu^{2}b_{2}b_{3}+16\beta^{2}c_{2}c_{4}+9\beta^{2}c_{3}^{2}-26\beta \nu b_{1}c_{4}-34\beta \nu b_{2}c_{3}-34\beta \nu b_{3}c_{2}-26\beta \nu b_{4}c_{1}\\ -10\beta \nu b_{5}c_{0}+10\nu^{2}b_{0}b_{4}+16\nu^{2}b_{1}b_{3}+9\nu^{2}b_{2}^{2}-2\nu^{2}c_{0}c_{4}-2\nu^{2}c_{1}c_{3}-\nu^{2}c_{2}^{2}-10\nu \beta b_{5}\\ +26\beta c_{1}c_{4}+34\beta c_{2}c_{3}-8\nu b_{0}c_{4}-14\nu b_{1}c_{3}-16\nu b_{2}c_{2}-14\nu b_{3}c_{1}-8\nu b_{4}c_{0}-10\nu b_{4}\\ +2\nu c_{4}-2b_{0}b_{4}-2b_{1}b_{3}-b_{2}^{2}+10c_{0}c_{4}+16c_{1}c_{3}+9c_{2}^{2}+10d_{1}d_{5}+16d_{2}d_{4}+9d_{3}^{2}\\ -2b_{4}+10c_{4}), \end{array}\\ \begin{array}{l} c_{6}=\frac{1}{6\beta (\nu \beta b_{1}-\beta c_{1}+\nu b_{0}-c_{0}-1)}(6\beta^{2}\nu^{2}b_{1}b_{6}+10\beta^{2}\nu^{2}b_{2}b_{5}+12\beta^{2}\nu^{2}b_{3}b_{4}\\ -10\beta^{2}\nu b_{2}c_{5}-12\beta^{2}\nu b_{3}c_{4}-12\beta^{2}\nu b_{4}c_{3}-10\beta^{2}\nu b_{5}c_{2}-6\beta^{2}\nu b_{6}c_{1}+6\beta \nu^{2}b_{0}b_{6}\\ +16\beta \nu^{2}b_{1}b_{5}+22\beta \nu^{2}b_{2}b_{4}+12\beta \nu^{2}b_{3}^{2}+10\beta^{2}c_{2}c_{5}+12\beta^{2}c_{3}c_{4}-16\beta \nu b_{1}c_{5}\\ -22\beta \nu b_{2}c_{4}-24\beta \nu b_{3}c_{3}-22\beta \nu b_{4}c_{2}-16\beta \nu b_{5}c_{1}-6\beta \nu b_{6}c_{0}+6\nu^{2}b_{0}b_{5}+10\nu^{2}b_{1}b_{4}\\ +12\nu^{2}b_{2}b_{3}-\nu^{2}c_{0}c_{5}-\nu^{2}c_{1}c_{4}-\nu^{2}c_{2}c_{3}-6\nu \beta b_{6}+16\beta c_{1}c_{5}+22\beta c_{2}c_{4}+12\beta c_{3}^{2}\\ -5\nu b_{0}c_{5}-9\nu b_{1}c_{4}-11\nu b_{2}c_{3}-11\nu b_{3}c_{2}-9\nu b_{4}c_{1}-5\nu b_{5}c_{0}-6\nu b_{5}+\nu c_{5}-b_{0}b_{5}-b_{1}b_{4}\\ -b_{2}b_{3}+6c_{0}c_{5}+10c_{1}c_{4}+12c_{2}c_{3}+6d_{1}d_{6}+10d_{2}d_{5}+12d_{3}d_{4}-b_{5}+6c_{5}), \end{array}\\ \begin{array}{l} c_{7}=\frac{1}{14\beta (\nu \beta b_{1}-\beta c_{1}+\nu b_{0}-c_{0}-1)}(14\beta^{2}\nu^{2}b_{1}b_{7}+24\beta^{2}\nu^{2}b_{2}b_{6}+30\beta^{2}\nu^{2}b_{3}b_{5}\\ +16\beta^{2}\nu^{2}b_{4}^{2}-24\beta^{2}\nu b_{2}c_{6}-30\beta^{2}\nu b_{3}c_{5}-32\beta^{2}\nu b_{4}c_{4}-30\beta^{2}\nu b_{5}c_{3}-24\beta^{2}\nu b_{6}c_{2}\\ -14\beta^{2}\nu b_{7}c_{1}+14\beta \nu^{2}b_{0}b_{7}+38\beta \nu^{2}b_{1}b_{6}+54\beta \nu^{2}b_{2}b_{5}+62\beta \nu^{2}b_{3}b_{4}+24\beta^{2}c_{2}c_{6}\\ +30\beta^{2}c_{3}c_{5}+16\beta^{2}c_{4}^{2}-38\beta \nu b_{1}c_{6}-54\beta \nu b_{2}c_{5}-62\beta \nu b_{3}c_{4}-62\beta \nu b_{4}c_{3}-54\beta \nu b_{5}c_{2}\\ -38\beta \nu b_{6}c_{1}-14\beta \nu b_{7}c_{0}+14\nu^{2}b_{0}b_{6}+24\nu^{2}b_{1}b_{5}+30\nu^{2}b_{2}b_{4}+16\nu^{2}b_{3}^{2}-2\nu^{2}c_{0}c_{6}\\ -2\nu^{2}c_{1}c_{5}-2\nu^{2}c_{2}c_{4}-\nu^{2}c_{3}^{2}-14\nu \beta b_{7}+38\beta c_{1}c_{6}+54\beta c_{2}c_{5}+62\beta c_{3}c_{4}-12\nu b_{0}c_{6}\\ -22\nu b_{1}c_{5}-28\nu b_{2}c_{4}-30\nu b_{3}c_{3}-28\nu b_{4}c_{2}-22\nu b_{5}c_{1}-12\nu b_{6}c_{0}-14\nu b_{6}+2\nu c_{6}-2b_{0}b_{6}\\ -2b_{1}b_{5}-2b_{2}b_{4}-b_{3}^{2}+14c_{0}c_{6}+24c_{1}c_{5}+30c_{2}c_{4}+16c_{3}^{2}+14d_{1}d_{7}+24d_{2}d_{6}+30d_{3}d_{5}\\ +16d_{4}^{2}-2b_{6}+14c_{6}), \end{array}\\ \begin{array}{l} c_{8}=\frac{1}{8\beta (\nu \beta b_{1}-\beta c_{1}+\nu b_{0}-c_{0}-1)}(8\beta^{2}\nu^{2}b_{1}b_{8}+14\beta^{2}\nu^{2}b_{2}b_{7}+18\beta^{2}\nu^{2}b_{3}b_{6}\\ +20\beta^{2}\nu^{2}b_{4}b_{5}-14\beta^{2}\nu b_{2}c_{7}-18\beta^{2}\nu b_{3}c_{6}-20\beta^{2}\nu b_{4}c_{5}-20\beta^{2}\nu b_{5}c_{4}-18\beta^{2}\nu b_{6}c_{3}\\ -14\beta^{2}\nu b_{7}c_{2}-8\beta^{2}\nu b_{8}c_{1}+8\beta \nu^{2}b_{0}b_{8}+22\beta \nu^{2}b_{1}b_{7}+32\beta \nu^{2}b_{2}b_{6}+38\beta \nu^{2}b_{3}b_{5}\\ +20\beta \nu^{2}b_{4}^{2}+14\beta^{2}c_{2}c_{7}+18\beta^{2}c_{3}c_{6}+20\beta^{2}c_{4}c_{5}-22\beta \nu b_{1}c_{7}-32\beta \nu b_{2}c_{6}-38\beta \nu b_{3}c_{5}\\ -40\beta \nu b_{4}c_{4}-38\beta \nu b_{5}c_{3}-32\beta \nu b_{6}c_{2}-22\beta \nu b_{7}c_{1}-8\beta \nu b_{8}c_{0}+8\nu^{2}b_{0}b_{7}+14\nu^{2}b_{1}b_{6}\\ +18\nu^{2}b_{2}b_{5}+20\nu^{2}b_{3}b_{4}-\nu^{2}c_{0}c_{7}-\nu^{2}c_{1}c_{6}-\nu^{2}c_{2}c_{5}-\nu^{2}c_{3}c_{4}-8\nu \beta b_{8}+22\beta c_{1}c_{7}\\ +32\beta c_{2}c_{6}+38\beta c_{3}c_{5}+20\beta c_{4}^{2}-7\nu b_{0}c_{7}-13\nu b_{1}c_{6}-17\nu b_{2}c_{5}-19\nu b_{3}c_{4}-19\nu b_{4}c_{3}\\ -17\nu b_{5}c_{2}-13\nu b_{6}c_{1}-7\nu b_{7}c_{0}-8\nu b_{7}+\nu c_{7}-b_{0}b_{7}-b_{1}b_{6}-b_{2}b_{5}-b_{3}b_{4}+8c_{0}c_{7}\\ +14c_{1}c_{6}+18c_{2}c_{5}+20c_{3}c_{4}+8d_{1}d_{8}+14d_{2}d_{7}+18d_{3}d_{6}+20d_{4}d_{5}-b_{7}+8c_{7}), \end{array}\\ \begin{array}{l} c_{9}=\frac{1}{18\beta (\nu \beta b_{1}-\beta c_{1}+\nu b_{0}-c_{0}-1)}(18\beta^{2}\nu^{2}b_{1}b_{9}+32\beta^{2}\nu^{2}b_{2}b_{8}+42\beta^{2}\nu^{2}b_{3}b_{7}\\ +48\beta^{2}\nu^{2}b_{4}b_{6}+25\beta^{2}\nu^{2}b_{5}^{2}-32\beta^{2}\nu b_{2}c_{8}-42\beta^{2}\nu b_{3}c_{7}-48\beta^{2}\nu b_{4}c_{6}-50\beta^{2}\nu b_{5}c_{5}\\ -48\beta^{2}\nu b_{6}c_{4}-42\beta^{2}\nu b_{7}c_{3}-32\beta^{2}\nu b_{8}c_{2}-18\beta^{2}\nu b_{9}c_{1}+18\beta \nu^{2}b_{0}b_{9}+50\beta \nu^{2}b_{1}b_{8}\\ +74\beta \nu^{2}b_{2}b_{7}+90\beta \nu^{2}b_{3}b_{6}+98\beta \nu^{2}b_{4}b_{5}+32\beta^{2}c_{2}c_{8}+42\beta^{2}c_{3}c_{7}+48\beta^{2}c_{4}c_{6}\\ +25\beta^{2}c_{5}^{2}-50\beta \nu b_{1}c_{8}-74\beta \nu b_{2}c_{7}-90\beta \nu b_{3}c_{6}-98\beta \nu b_{4}c_{5}-98\beta \nu b_{5}c_{4}-90\beta \nu b_{6}c_{3}\\ -74\beta \nu b_{7}c_{2}-50\beta \nu b_{8}c_{1}-18\beta \nu b_{9}c_{0}+18\nu^{2}b_{0}b_{8}+32\nu^{2}b_{1}b_{7}+42\nu^{2}b_{2}b_{6}+48\nu^{2}b_{3}b_{5}\\ +25\nu^{2}b_{4}^{2}-2\nu^{2}c_{0}c_{8}-2\nu^{2}c_{1}c_{7}-2\nu^{2}c_{2}c_{6}-2\nu^{2}c_{3}c_{5}-\nu^{2}c_{4}^{2}-18\nu \beta b_{9}+50\beta c_{1}c_{8}\\ +74\beta c_{2}c_{7}+90\beta c_{3}c_{6}+98\beta c_{4}c_{5}-16\nu b_{0}c_{8}-30\nu b_{1}c_{7}-40\nu b_{2}c_{6}-46\nu b_{3}c_{5}-48\nu b_{4}c_{4}\\ -46\nu b_{5}c_{3}-40\nu b_{6}c_{2}-30\nu b_{7}c_{1}-16\nu b_{8}c_{0}-18\nu b_{8}+2\nu c_{8}-2b_{0}b_{8}-2b_{1}b_{7}-2b_{2}b_{6}\\ -2b_{3}b_{5}-b_{4}^{2}+18c_{0}c_{8}+32c_{1}c_{7}+42c_{2}c_{6}+48c_{3}c_{5}+25c_{4}^{2}+18d_{1}d_{9}+32d_{2}d_{8}+42d_{3}d_{7}\\ +48d_{4}d_{6}+25d_{5}^{2}-2b_{8}+18c_{8}), \end{array}\\ \begin{array}{l} c_{10}=\frac{1}{10\beta (\nu \beta b_{1}-\beta c_{1}+\nu b_{0}-c_{0}-1)}(\nu c_{9}-b_{0}b_{9}-b_{1}b_{8}-b_{2}b_{7}-b_{3}b_{6}-b_{4}b_{5}-10\nu b_{9}\\ +30\beta c_{5}^{2}+10c_{0}c_{9}+18c_{1}c_{8}+24c_{2}c_{7}+28c_{3}c_{6}+30c_{4}c_{5}+30d_{5}d_{6}+10d_{1}d_{10}+18d_{2}d_{9}\\ +28d_{4}d_{7}+24d_{3}d_{8}+10c_{9}-b_{9}-\nu^{2}c_{0}c_{9}-\nu^{2}c_{1}c_{8}-\nu^{2}c_{2}c_{7}-\nu^{2}c_{3}c_{6}-\nu^{2}c_{4}c_{5}-9\nu b_{0}c_{9}\\ -17\nu b_{1}c_{8}-23\nu b_{2}c_{7}-27\nu b_{3}c_{6}-29\nu b_{4}c_{5}-29\nu b_{5}c_{4}-27\nu b_{6}c_{3}-23\nu b_{7}c_{2}-17\nu b_{8}c_{1}\\ -9\nu b_{9}c_{0}-10\nu \beta b_{10}+30\beta \nu^{2}b_{5}^{2}+18\beta^{2}c_{2}c_{9}+24\beta^{2}c_{3}c_{8}+28\beta^{2}c_{4}c_{7}+30\beta^{2}c_{5}c_{6}\\ +10\nu^{2}b_{0}b_{9}+18\nu^{2}b_{1}b_{8}+24\nu^{2}b_{2}b_{7}+28\nu^{2}b_{3}b_{6}+30\nu^{2}b_{4}b_{5}+28\beta c_{1}c_{9}+42\beta c_{2}c_{8}\\ +52\beta c_{3}c_{7}+58\beta c_{4}c_{6}+10\beta^{2}\nu^{2}b_{1}b_{10}+18\beta^{2}\nu^{2}b_{2}b_{9}+24\beta^{2}\nu^{2}b_{3}b_{8}+28\beta^{2}\nu^{2}b_{4}b_{7}\\ +30\beta^{2}\nu^{2}b_{5}b_{6}-18\beta^{2}\nu b_{2}c_{9}-24\beta^{2}\nu b_{3}c_{8}-28\beta^{2}\nu b_{4}c_{7}-30\beta^{2}\nu b_{5}c_{6}-30\beta^{2}\nu b_{6}c_{5}\\ -28\beta^{2}\nu b_{7}c_{4}-24\beta^{2}\nu b_{8}c_{3}-18\beta^{2}\nu b_{9}c_{2}-10\beta^{2}\nu b_{10}c_{1}+10\beta \nu^{2}b_{0}b_{10}+28\beta \nu^{2}b_{1}b_{9}\\ +42\beta \nu^{2}b_{2}b_{8}+52\beta \nu^{2}b_{3}b_{7}+58\beta \nu^{2}b_{4}b_{6}-28\beta \nu b_{1}c_{9}-42\beta \nu b_{2}c_{8}-52\beta \nu b_{3}c_{7}\\ -58\beta \nu b_{4}c_{6}-60\beta \nu b_{5}c_{5}-58\beta \nu b_{6}c_{4}-52\beta \nu b_{7}c_{3}-42\beta \nu b_{8}c_{2}-28\beta \nu b_{9}c_{1}\\ -10\beta \nu b_{10}c_{0}), \end{array}\\ \begin{array}{l} c_{11}=\frac{1}{22\beta (\nu \beta b_{1}-\beta c_{1}+\nu b_{0}-c_{0}-1)}(2\nu c_{10}-\nu^{2}c_{5}^{2}-2b_{0}b_{10}-2b_{1}b_{9}-2b_{2}b_{8}-2b_{3}b_{7}\\ -2b_{4}b_{6}-22\nu b_{10}+36\beta^{2}c_{6}^{2}+36\nu^{2}b_{5}^{2}+22c_{0}c_{10}+40c_{1}c_{9}+54c_{2}c_{8}+64c_{3}c_{7}+70c_{4}c_{6}\\ +70d_{5}d_{7}+22d_{1}d_{11}+40d_{2}d_{10}+64d_{4}d_{8}+54d_{3}d_{9}+36d_{6}^{2}+22c_{10}-2b_{10}-2\nu^{2}c_{0}c_{10}\\ -2\nu^{2}c_{1}c_{9}-2\nu^{2}c_{2}c_{8}-2\nu^{2}c_{3}c_{7}-2\nu^{2}c_{4}c_{6}-20\nu b_{0}c_{10}-38\nu b_{1}c_{9}-52\nu b_{2}c_{8}-62\nu b_{3}c_{7}\\ -68\nu b_{4}c_{6}-70\nu b_{5}c_{5}-68\nu b_{6}c_{4}-62\nu b_{7}c_{3}-52\nu b_{8}c_{2}-38\nu b_{9}c_{1}-20\nu b_{10}c_{0}-22\nu \beta b_{11}\\ +36\beta^{2}\nu^{2}b_{6}^{2}+62\beta c_{1}c_{10}+94\beta c_{2}c_{9}+118\beta c_{3}c_{8}+134\beta c_{4}c_{7}+142\beta c_{5}c_{6}+40\beta^{2}c_{2}c_{10}\\ +54\beta^{2}c_{3}c_{9}+64\beta^{2}c_{4}c_{8}+70\beta^{2}c_{5}c_{7}+22\nu^{2}b_{0}b_{10}+40\nu^{2}b_{1}b_{9}+54\nu^{2}b_{2}b_{8}+64\nu^{2}b_{3}b_{7}\\ +70\nu^{2}b_{4}b_{6}+22\beta^{2}\nu^{2}b_{1}b_{11}+40\beta^{2}\nu^{2}b_{2}b_{10}+54\beta^{2}\nu^{2}b_{3}b_{9}+64\beta^{2}\nu^{2}b_{4}b_{8}+70\beta^{2}\nu^{2}b_{5}b_{7}\\ -40\beta^{2}\nu b_{2}c_{10}-54\beta^{2}\nu b_{3}c_{9}-64\beta^{2}\nu b_{4}c_{8}-70\beta^{2}\nu b_{5}c_{7}-72\beta^{2}\nu b_{6}c_{6}-70\beta^{2}\nu b_{7}c_{5}\\ -64\beta^{2}\nu b_{8}c_{4}-54\beta^{2}\nu b_{9}c_{3}-40\beta^{2}\nu b_{10}c_{2}-22\beta^{2}\nu b_{11}c_{1}+22\beta \nu^{2}b_{0}b_{11}+62\beta \nu^{2}b_{1}b_{10}\\ +94\beta \nu^{2}b_{2}b_{9}+118\beta \nu^{2}b_{3}b_{8}+134\beta \nu^{2}b_{4}b_{7}+142\beta \nu^{2}b_{5}b_{6}-62\beta \nu b_{1}c_{10}-94\beta \nu b_{2}c_{9}\\ -118\beta \nu b_{3}c_{8}-134\beta \nu b_{4}c_{7}-142\beta \nu b_{5}c_{6}-142\beta \nu b_{6}c_{5}-134\beta \nu b_{7}c_{4}-118\beta \nu b_{8}c_{3}\\ -94\beta \nu b_{9}c_{2}-62\beta \nu b_{10}c_{1}-22\beta \nu b_{11}c_{0}+36c_{5}^{2}-b_{5}^{2}), \end{array}\\ \begin{array}{l} c_{12}=\frac{1}{12\beta (\nu \beta b_{1}-\beta c_{1}+\nu b_{0}-c_{0}-1)}(\nu c_{11}-12\nu b_{11}-b_{0}b_{11}-b_{1}b_{10}-b_{2}b_{9}-b_{3}b_{8}-b_{4}b_{7}\\ -b_{5}b_{6}+42\beta c_{6}^{2}+12c_{0}c_{11}+22c_{1}c_{10}+30c_{2}c_{9}+36c_{3}c_{8}+40c_{4}c_{7}+42c_{5}c_{6}+40d_{5}d_{8}\\ +42d_{6}d_{7}+12d_{1}d_{12}+22d_{2}d_{11}+36d_{4}d_{9}+30d_{3}d_{10}+12c_{11}-b_{11}-\nu^{2}c_{0}c_{11}-\nu^{2}c_{1}c_{10}\\ -\nu^{2}c_{2}c_{9}-\nu^{2}c_{3}c_{8}-\nu^{2}c_{4}c_{7}-41\nu b_{5}c_{6}-41\nu b_{6}c_{5}-39\nu b_{7}c_{4}-35\nu b_{8}c_{3}-29\nu b_{9}c_{2}-21\nu b_{10}c_{1}\\ -11\nu b_{11}c_{0}-\nu^{2}c_{5}c_{6}-11\nu b_{0}c_{11}-21\nu b_{1}c_{10}-29\nu b_{2}c_{9}-35\nu b_{3}c_{8}-39\nu b_{4}c_{7}-12\nu \beta b_{12}\\ +42\beta \nu^{2}b_{6}^{2}+22\beta^{2}c_{2}c_{11}+30\beta^{2}c_{3}c_{10}+36\beta^{2}c_{4}c_{9}+40\beta^{2}c_{5}c_{8}+42\beta^{2}c_{6}c_{7}+12\nu^{2}b_{0}b_{11}\\ +22\nu^{2}b_{1}b_{10}+30\nu^{2}b_{2}b_{9}+36\nu^{2}b_{3}b_{8}+40\nu^{2}b_{4}b_{7}+42\nu^{2}b_{5}b_{6}+34\beta c_{1}c_{11}+52\beta c_{2}c_{10}\\ +66\beta c_{3}c_{9}+76\beta c_{4}c_{8}+82\beta c_{5}c_{7}+12\beta^{2}\nu^{2}b_{1}b_{12}+22\beta^{2}\nu^{2}b_{2}b_{11}+30\beta^{2}\nu^{2}b_{3}b_{10}\\ +36\beta^{2}\nu^{2}b_{4}b_{9}+40\beta^{2}\nu^{2}b_{5}b_{8}+42\beta^{2}\nu^{2}b_{6}b_{7}-22\beta^{2}\nu b_{2}c_{11}-30\beta^{2}\nu b_{3}c_{10}-36\beta^{2}\nu b_{4}c_{9}\\ -40\beta^{2}\nu b_{5}c_{8}-42\beta^{2}\nu b_{6}c_{7}-42\beta^{2}\nu b_{7}c_{6}-40\beta^{2}\nu b_{8}c_{5}-36\beta^{2}\nu b_{9}c_{4}-30\beta^{2}\nu b_{10}c_{3}\\ -22\beta^{2}\nu b_{11}c_{2}-12\beta^{2}\nu b_{12}c_{1}+12\beta \nu^{2}b_{0}b_{12}+34\beta \nu^{2}b_{1}b_{11}+52\beta \nu^{2}b_{2}b_{10}+66\beta \nu^{2}b_{3}b_{9}\\ +76\beta \nu^{2}b_{4}b_{8}+82\beta \nu^{2}b_{5}b_{7}-34\beta \nu b_{1}c_{11}-52\beta \nu b_{2}c_{10}-66\beta \nu b_{3}c_{9}-76\beta \nu b_{4}c_{8}\\ -82\beta \nu b_{5}c_{7}-84\beta \nu b_{6}c_{6}-82\beta \nu b_{7}c_{5}-76\beta \nu b_{8}c_{4}-66\beta \nu b_{9}c_{3}-52\beta \nu b_{10}c_{2}\\ -34\beta \nu b_{11}c_{1}-12\beta \nu b_{12}c_{0}), \end{array}\\ d_{1}=\frac{(\alpha +\beta )(\alpha -\beta )Q}{\sqrt{-Q^{2}\alpha^{4}+2Q^{2}\alpha^{2}\beta^{2}-Q^{2}\beta^{4}+4\beta^{2}c_{0}^{2}}},\\ \begin{array}{l} d_{2}=\frac{\beta Q}{Q^{2}\alpha^{4}-2Q^{2}\alpha^{2}\beta^{2}+Q^{2}\beta^{4}-4\beta^{2}b_{0}^{2}}(-Q^{2}\alpha^{4}+2Q^{2}\alpha^{2}\beta^{2}-Q^{2}\beta^{4}\\ +2\alpha^{2}b_{0}c_{0}+4\beta^{2}b_{0}^{2}-2\beta^{2}b_{0}c_{0})\sqrt{-\frac{1}{Q^{2}\alpha^{4}-2Q^{2}\alpha^{2}\beta^{2}+Q^{2}\beta^{4}-4\beta^{2}b_{0}^{2}}}, \end{array}\\ \begin{array}{l} d_{3}=-\frac{1}{6(Q\alpha^{2}d_{1}-Q\beta^{2}d_{1}-2\beta b_{0}f_{0}-2d_{1}^{2}h_{0})}(4Q\alpha^{2}d_{2}^{2}-Q\alpha^{2}f_{1}^{2}-4Q\beta^{2}d_{2}^{2}\\ +Q\beta^{2}f_{1}^{2}-4Q\beta f_{0}f_{1}-8\beta b_{1}d_{2}f_{0}-8d_{1}d_{2}^{2}h_{0}-4d_{1}f_{0}^{2}h_{2}-4d_{1}f_{0}f_{1}h_{1}+2d_{1}f_{1}^{2}h_{0}\\ -2Qf_{0}^{2}-8b_{0}d_{2}f_{0}), \end{array}\\ \begin{array}{l} d_{4}=-\frac{1}{4(Q\alpha^{2}d_{1}-Q\beta^{2}d_{1}-2\beta b_{0}f_{0}-2d_{1}^{2}h_{0})}(6Q\alpha^{2}d_{2}d_{3}-Q\alpha^{2}f_{1}f_{2}-6Q\beta^{2}d_{2}d_{3}\\ +Q\beta^{2}f_{1}f_{2}-2Q\beta f_{0}f_{2}-6\beta b_{1}d_{3}f_{0}-4\beta b_{2}d_{2}f_{0}-12d_{1}d_{2}d_{3}h_{0}-2d_{1}f_{0}^{2}h_{3}-2d_{1}f_{0}f_{1}h_{2}\\ -2d_{1}f_{0}f_{2}h_{1}+2d_{1}f_{1}f_{2}h_{0}-Qf_{0}f_{1}-6b_{0}d_{3}f_{0}-4b_{1}d_{2}f_{0}), \end{array}\\ \begin{array}{l} d_{5}=-\frac{1}{10(Q\alpha^{2}d_{1}-Q\beta^{2}d_{1}-2\beta b_{0}f_{0}-2d_{1}^{2}h_{0})}(16Q\alpha^{2}d_{2}d_{4}+9Q\alpha^{2}d_{3}^{2}-2Q\alpha^{2}f_{1}f_{3}\\ -Q\alpha^{2}f_{2}^{2}-16Q\beta^{2}d_{2}d_{4}-9Q\beta^{2}d_{3}^{2}+2Q\beta^{2}f_{1}f_{3}+Q\beta^{2}f_{2}^{2}-4Q\beta f_{0}f_{3}-16\beta b_{1}d_{4}f_{0}\\ -12\beta b_{2}d_{3}f_{0}-8\beta b_{3}d_{2}f_{0}-32d_{1}d_{2}d_{4}h_{0}-18d_{1}d_{3}^{2}h_{0}-4d_{1}f_{0}^{2}h_{4}-4d_{1}f_{0}f_{1}h_{3}\\ -4d_{1}f_{0}f_{2}h_{2}-4d_{1}f_{0}f_{3}h_{1}+4d_{1}f_{1}f_{3}h_{0}+2d_{1}f_{2}^{2}h_{0}-2Qf_{0}f_{2}-16b_{0}d_{4}f_{0}-12b_{1}d_{3}f_{0}\\ -8b_{2}d_{2}f_{0}), \end{array}\\ \begin{array}{l} d_{6}=-\frac{1}{6(Q\alpha^{2}d_{1}-Q\beta^{2}d_{1}-2\beta b_{0}f_{0}-2d_{1}^{2}h_{0})}(10Q\alpha^{2}d_{2}d_{5}+12Q\alpha^{2}d_{3}d_{4}-Q\alpha^{2}f_{1}f_{4}\\ -Q\alpha^{2}f_{2}f_{3}-10Q\beta^{2}d_{2}d_{5}-12Q\beta^{2}d_{3}d_{4}+Q\beta^{2}f_{1}f_{4}+Q\beta^{2}f_{2}f_{3}-2Q\beta f_{0}f_{4}\\ -10\beta b_{1}d_{5}f_{0}-8\beta b_{2}d_{4}f_{0}-6\beta b_{3}d_{3}f_{0}-4\beta b_{4}d_{2}f_{0}-20d_{1}d_{2}d_{5}h_{0}-24d_{1}d_{3}d_{4}h_{0}\\ -2d_{1}f_{0}^{2}h_{5}-2d_{1}f_{0}f_{1}h_{4}-2d_{1}f_{0}f_{2}h_{3}-2d_{1}f_{0}f_{3}h_{2}-2d_{1}f_{0}f_{4}h_{1}+2d_{1}f_{1}f_{4}h_{0}\\ +2d_{1}f_{2}f_{3}h_{0}-Qf_{0}f_{3}-10b_{0}d_{5}f_{0}-8b_{1}d_{4}f_{0}-6b_{2}d_{3}f_{0}-4b_{3}d_{2}f_{0}), \end{array}\\ \begin{array}{l} d_{7}=-\frac{1}{14(Q\alpha^{2}d_{1}-Q\beta^{2}d_{1}-2\beta b_{0}f_{0}-2d_{1}^{2}h_{0})}(24Q\alpha^{2}d_{2}d_{6}+30Q\alpha^{2}d_{3}d_{5}+16Q\alpha^{2}d_{4}^{2}\\ -2Q\alpha^{2}f_{1}f_{5}-2Q\alpha^{2}f_{2}f_{4}-Q\alpha^{2}f_{3}^{2}-24Q\beta^{2}d_{2}d_{6}-30Q\beta^{2}d_{3}d_{5}-16Q\beta^{2}d_{4}^{2}+2Q\beta^{2}f_{1}f_{5}\\ +2Q\beta^{2}f_{2}f_{4}+Q\beta^{2}f_{3}^{2}-4Q\beta f_{0}f_{5}-24\beta b_{1}d_{6}f_{0}-20\beta b_{2}d_{5}f_{0}-16\beta b_{3}d_{4}f_{0}-12\beta b_{4}d_{3}f_{0}\\ -8\beta b_{5}d_{2}f_{0}-48d_{1}d_{2}d_{6}h_{0}-60d_{1}d_{3}d_{5}h_{0}-32d_{1}d_{4}^{2}h_{0}-4d_{1}f_{0}^{2}h_{6}-4d_{1}f_{0}f_{1}h_{5}-4d_{1}f_{0}f_{2}h_{4}\\ -4d_{1}f_{0}f_{3}h_{3}-4d_{1}f_{0}f_{4}h_{2}-4d_{1}f_{0}f_{5}h_{1}+4d_{1}f_{1}f_{5}h_{0}+4d_{1}f_{2}f_{4}h_{0}+2d_{1}f_{3}^{2}h_{0}-2Qf_{0}f_{4}\\ -24b_{0}d_{6}f_{0}-20b_{1}d_{5}f_{0}-16b_{2}d_{4}f_{0}-12b_{3}d_{3}f_{0}-8b_{4}d_{2}f_{0}), \end{array}\\ \begin{array}{l} d_{8}=-\frac{1}{8(Q\alpha^{2}d_{1}-Q\beta^{2}d_{1}-2\beta b_{0}f_{0}-2d_{1}^{2}h_{0})}(-2d_{1}f_{0}f_{6}h_{1}+2d_{1}f_{1}f_{6}h_{0}+2d_{1}f_{2}f_{5}h_{0}\\ +2d_{1}f_{3}f_{4}h_{0}+14Q\alpha^{2}d_{2}d_{7}+18Q\alpha^{2}d_{3}d_{6}+20Q\alpha^{2}d_{4}d_{5}-Q\alpha^{2}f_{1}f_{6}-Q\alpha^{2}f_{2}f_{5}\\ -Q\alpha^{2}f_{3}f_{4}-14Q\beta^{2}d_{2}d_{7}-18Q\beta^{2}d_{3}d_{6}-20Q\beta^{2}d_{4}d_{5}+Q\beta^{2}f_{1}f_{6}+Q\beta^{2}f_{2}f_{5}\\ +Q\beta^{2}f_{3}f_{4}-2Q\beta f_{0}f_{6}-14\beta b_{1}d_{7}f_{0}-12\beta b_{2}d_{6}f_{0}-10\beta b_{3}d_{5}f_{0}-8\beta b_{4}d_{4}f_{0}\\ -6\beta b_{5}d_{3}f_{0}-4\beta b_{6}d_{2}f_{0}-28d_{1}d_{2}d_{7}h_{0}-36d_{1}d_{3}d_{6}h_{0}-40d_{1}d_{4}d_{5}h_{0}-2d_{1}f_{0}f_{1}h_{6}\\ -2d_{1}f_{0}f_{2}h_{5}-2d_{1}f_{0}f_{3}h_{4}-2d_{1}f_{0}f_{4}h_{3}-2d_{1}f_{0}f_{5}h_{2}-Qf_{0}f_{5}-14b_{0}d_{7}f_{0}-12b_{1}d_{6}f_{0}\\ -10b_{2}d_{5}f_{0}-8b_{3}d_{4}f_{0}-6b_{4}d_{3}f_{0}-4b_{5}d_{2}f_{0}-2d_{1}f_{0}^{2}h_{7}), \end{array}\\ \begin{array}{l} d_{9}=-\frac{1}{18(Q\alpha^{2}d_{1}-Q\beta^{2}d_{1}-2\beta b_{0}f_{0}-2d_{1}^{2}h_{0})}(25Q\alpha^{2}d_{5}^{2}-Q\alpha^{2}f_{4}^{2}-25Q\beta^{2}d_{5}^{2}\\ +Q\beta^{2}f_{4}^{2}-2Qf_{0}f_{6}-32b_{0}d_{8}f_{0}-28b_{1}d_{7}f_{0}-24b_{2}d_{6}f_{0}-20b_{3}d_{5}f_{0}-16b_{4}d_{4}f_{0}\\ -12b_{5}d_{3}f_{0}-8b_{6}d_{2}f_{0}-50d_{1}d_{5}^{2}h_{0}-4d_{1}f_{0}^{2}h_{8}+2d_{1}f_{4}^{2}h_{0}+48Q\alpha^{2}d_{4}d_{6}-2Q\alpha^{2}f_{1}f_{7}\\ -2Q\alpha^{2}f_{2}f_{6}-2Q\alpha^{2}f_{3}f_{5}-32Q\beta^{2}d_{2}d_{8}-42Q\beta^{2}d_{3}d_{7}-48Q\beta^{2}d_{4}d_{6}+2Q\beta^{2}f_{1}f_{7}\\ +2Q\beta^{2}f_{2}f_{6}+2Q\beta^{2}f_{3}f_{5}-4Q\beta f_{0}f_{7}-32\beta b_{1}d_{8}f_{0}-28\beta b_{2}d_{7}f_{0}-24\beta b_{3}d_{6}f_{0}\\ -20\beta b_{4}d_{5}f_{0}-16\beta b_{5}d_{4}f_{0}-12\beta b_{6}d_{3}f_{0}-8\beta b_{7}d_{2}f_{0}-64d_{1}d_{2}d_{8}h_{0}-84d_{1}d_{3}d_{7}h_{0}\\ -96d_{1}d_{4}d_{6}h_{0}-4d_{1}f_{0}f_{1}h_{7}-4d_{1}f_{0}f_{2}h_{6}-4d_{1}f_{0}f_{3}h_{5}-4d_{1}f_{0}f_{4}h_{4}-4d_{1}f_{0}f_{5}h_{3}\\ -4d_{1}f_{0}f_{6}h_{2}-4d_{1}f_{0}f_{7}h_{1}+4d_{1}f_{1}f_{7}h_{0}+4d_{1}f_{2}f_{6}h_{0}+4d_{1}f_{3}f_{5}h_{0}+32Q\alpha^{2}d_{2}d_{8}\\ +42Q\alpha^{2}d_{3}d_{7}), \end{array}\\ \begin{array}{l} d_{10}=-\frac{1}{10(Q\alpha^{2}d_{1}-Q\beta^{2}d_{1}-2\beta b_{0}f_{0}-2d_{1}^{2}h_{0})}(-18Q\beta^{2}d_{2}d_{9}-24Q\beta^{2}d_{3}d_{8}\\ -28Q\beta^{2}d_{4}d_{7}-30Q\beta^{2}d_{5}d_{6}+Q\beta^{2}f_{1}f_{8}+Q\beta^{2}f_{2}f_{7}+Q\beta^{2}f_{3}f_{6}+Q\beta^{2}f_{4}f_{5}\\ -2Q\beta f_{0}f_{8}-18\beta b_{1}d_{9}f_{0}-16\beta b_{2}d_{8}f_{0}-14\beta b_{3}d_{7}f_{0}-12\beta b_{4}d_{6}f_{0}-10\beta b_{5}d_{5}f_{0}\\ -8\beta b_{6}d_{4}f_{0}-6\beta b_{7}d_{3}f_{0}-4\beta b_{8}d_{2}f_{0}-36d_{1}d_{2}d_{9}h_{0}-48d_{1}d_{3}d_{8}h_{0}-56d_{1}d_{4}d_{7}h_{0}\\ -60d_{1}d_{5}d_{6}h_{0}-2d_{1}f_{0}f_{1}h_{8}-2d_{1}f_{0}f_{2}h_{7}-2d_{1}f_{0}f_{3}h_{6}-2d_{1}f_{0}f_{4}h_{5}-2d_{1}f_{0}f_{5}h_{4}\\ -2d_{1}f_{0}f_{6}h_{3}-2d_{1}f_{0}f_{7}h_{2}-2d_{1}f_{0}f_{8}h_{1}+2d_{1}f_{1}f_{8}h_{0}+2d_{1}f_{2}f_{7}h_{0}+2d_{1}f_{3}f_{6}h_{0}\\ +2d_{1}f_{4}f_{5}h_{0}+18Q\alpha^{2}d_{2}d_{9}+24Q\alpha^{2}d_{3}d_{8}+28Q\alpha^{2}d_{4}d_{7}+30Q\alpha^{2}d_{5}d_{6}-Q\alpha^{2}f_{1}f_{8}\\ -Q\alpha^{2}f_{2}f_{7}-Q\alpha^{2}f_{3}f_{6}-Q\alpha^{2}f_{4}f_{5}-2d_{1}f_{0}^{2}h_{9}-Qf_{0}f_{7}-18b_{0}d_{9}f_{0}-16b_{1}d_{8}f_{0}\\ -14b_{2}d_{7}f_{0}-12b_{3}d_{6}f_{0}-10b_{4}d_{5}f_{0}-8b_{5}d_{4}f_{0}-6b_{6}d_{3}f_{0}-4b_{7}d_{2}f_{0}), \end{array}\\ \begin{array}{l} d_{11}=-\frac{1}{22(Q\alpha^{2}d_{1}-Q\beta^{2}d_{1}-2\beta b_{0}f_{0}-2d_{1}^{2}h_{0})}(-4d_{1}f_{0}f_{1}h_{9}-4d_{1}f_{0}f_{2}h_{8}-4d_{1}f_{0}f_{3}h_{7}\\ -4d_{1}f_{0}f_{4}h_{6}-4d_{1}f_{0}f_{5}h_{5}-4d_{1}f_{0}f_{6}h_{4}-4d_{1}f_{0}f_{7}h_{3}-4d_{1}f_{0}f_{8}h_{2}-4d_{1}f_{0}f_{9}h_{1}\\ -80d_{1}d_{2}d_{10}h_{0}-108d_{1}d_{3}d_{9}h_{0}-128d_{1}d_{4}d_{8}h_{0}-140d_{1}d_{5}d_{7}h_{0}+4d_{1}f_{1}f_{9}h_{0}+4d_{1}f_{2}f_{8}h_{0}\\ +4d_{1}f_{3}f_{7}h_{0}+4d_{1}f_{4}f_{6}h_{0}+40Q\alpha^{2}d_{2}d_{10}+54Q\alpha^{2}d_{3}d_{9}+64Q\alpha^{2}d_{4}d_{8}+70Q\alpha^{2}d_{5}d_{7}\\ -2Q\alpha^{2}f_{1}f_{9}-2Q\alpha^{2}f_{2}f_{8}-2Q\alpha^{2}f_{3}f_{7}-2Q\alpha^{2}f_{4}f_{6}-40Q\beta^{2}d_{2}d_{10}-54Q\beta^{2}d_{3}d_{9}\\ -64Q\beta^{2}d_{4}d_{8}-70Q\beta^{2}d_{5}d_{7}+2Q\beta^{2}f_{1}f_{9}+2Q\beta^{2}f_{2}f_{8}+2Q\beta^{2}f_{3}f_{7}+2Q\beta^{2}f_{4}f_{6}\\ -4Q\beta f_{0}f_{9}-40\beta b_{1}d_{10}f_{0}-36\beta b_{2}d_{9}f_{0}-32\beta b_{3}d_{8}f_{0}-28\beta b_{4}d_{7}f_{0}-24\beta b_{5}d_{6}f_{0}\\ -20\beta b_{6}d_{5}f_{0}-16\beta b_{7}d_{4}f_{0}-28b_{3}d_{7}f_{0}-12\beta b_{8}d_{3}f_{0}-8\beta b_{9}d_{2}f_{0}-24b_{4}d_{6}f_{0}\\ -20b_{5}d_{5}f_{0}-16b_{6}d_{4}f_{0}-12b_{7}d_{3}f_{0}-8b_{8}d_{2}f_{0}-4d_{1}f_{0}^{2}h_{10}-72d_{1}d_{6}^{2}h_{0}+2d_{1}f_{5}^{2}h_{0}\\ +36Q\alpha^{2}d_{6}^{2}-Q\alpha^{2}f_{5}^{2}-36Q\beta^{2}d_{6}^{2}+Q\beta^{2}f_{5}^{2}-2Qf_{0}f_{8}-40b_{0}d_{10}f_{0}-36b_{1}d_{9}f_{0}\\ -32b_{2}d_{8}f_{0}), \end{array}\\ \begin{array}{l} d_{12}=-\frac{1}{12(Q\alpha^{2}d_{1}-Q\beta^{2}d_{1}-2\beta b_{0}f_{0}-2d_{1}^{2}h_{0})}(-2d_{1}f_{0}^{2}h_{11}-Qf_{0}f_{9}-22b_{0}d_{11}f_{0}\\ -20b_{1}d_{10}f_{0}-18b_{2}d_{9}f_{0}-16b_{3}d_{8}f_{0}-14b_{4}d_{7}f_{0}-12b_{5}d_{6}f_{0}-10b_{6}d_{5}f_{0}-8b_{7}d_{4}f_{0}\\ -6b_{8}d_{3}f_{0}-4b_{9}d_{2}f_{0}+Q\beta^{2}f_{3}f_{8}+Q\beta^{2}f_{4}f_{7}+Q\beta^{2}f_{5}f_{6}-2Q\beta f_{0}f_{10}-22\beta b_{1}d_{11}f_{0}\\ -20\beta b_{2}d_{10}f_{0}-18\beta b_{3}d_{9}f_{0}-16\beta b_{4}d_{8}f_{0}-14\beta b_{5}d_{7}f_{0}-12\beta b_{6}d_{6}f_{0}-10\beta b_{7}d_{5}f_{0}\\ -8\beta b_{8}d_{4}f_{0}-6\beta b_{9}d_{3}f_{0}-4\beta b_{10}d_{2}f_{0}-2d_{1}f_{0}f_{1}h_{10}-2d_{1}f_{0}f_{2}h_{9}-2d_{1}f_{0}f_{3}h_{8}\\ -2d_{1}f_{0}f_{4}h_{7}-2d_{1}f_{0}f_{5}h_{6}-2d_{1}f_{0}f_{6}h_{5}-2d_{1}f_{0}f_{7}h_{4}-2d_{1}f_{0}f_{8}h_{3}-2d_{1}f_{0}f_{9}h_{2}\\ -2d_{1}f_{0}f_{10}h_{1}-44d_{1}d_{2}d_{11}h_{0}-60d_{1}d_{3}d_{10}h_{0}-72d_{1}d_{4}d_{9}h_{0}-80d_{1}d_{5}d_{8}h_{0}-84d_{1}d_{6}d_{7}h_{0}\\ +2d_{1}f_{1}f_{10}h_{0}+2d_{1}f_{2}f_{9}h_{0}+2d_{1}f_{3}f_{8}h_{0}+2d_{1}f_{4}f_{7}h_{0}+2d_{1}f_{5}f_{6}h_{0}+22Q\alpha^{2}d_{2}d_{11}\\ +30Q\alpha^{2}d_{3}d_{10}+36Q\alpha^{2}d_{4}d_{9}+40Q\alpha^{2}d_{5}d_{8}+42Q\alpha^{2}d_{6}d_{7}-Q\alpha^{2}f_{1}f_{10}-Q\alpha^{2}f_{2}f_{9}\\ -Q\alpha^{2}f_{3}f_{8}-Q\alpha^{2}f_{4}f_{7}-Q\alpha^{2}f_{5}f_{6}-22Q\beta^{2}d_{2}d_{11}-30Q\beta^{2}d_{3}d_{10}-36Q\beta^{2}d_{4}d_{9}\\ -40Q\beta^{2}d_{5}d_{8}-42Q\beta^{2}d_{6}d_{7}+Q\beta^{2}f_{1}f_{10}+Q\beta^{2}f_{2}f_{9}), \end{array}\\ f_{0}=2b_{0}\beta \sqrt{-\frac{1}{Q^{2}\alpha^{4}-2Q^{2}\alpha^{2}\beta^{2}+Q^{2}\beta^{4}-4\beta^{2}b_{0}^{2}}},\\ \begin{array}{l} f_{1}=\frac{Q^{2}(\alpha^{2}-\beta^{2})}{(Q^{2}\alpha^{4}-2Q^{2}\alpha^{2}\beta^{2}+Q^{2}\beta^{4}-4\beta^{2}b_{0}^{2})b_{0}}(-Q^{2}\alpha^{4}+2Q^{2}\alpha^{2}\beta^{2}-Q^{2}\beta^{4}+2\alpha^{2}b_{0}c_{0}\\ +4\beta^{2}b_{0}^{2}-2\beta^{2}b_{0}c_{0})\sqrt{-\frac{1}{Q^{2}\alpha^{4}-2Q^{2}\alpha^{2}\beta^{2}+Q^{2}\beta^{4}-4\beta^{2}b_{0}^{2}}}, \end{array}\\ \begin{array}{l} f_{2}=\frac{1}{Q\alpha^{2}d_{1}-Q\beta^{2}d_{1}-2\beta b_{0}f_{0}-2d_{1}^{2}h_{0}}(2Q\beta d_{1}f_{1}-4\beta b_{0}d_{2}^{2}+\beta b_{0}f_{1}^{2}+4\beta b_{1}d_{1}d_{2}\\ +2d_{1}^{2}f_{0}h_{2}+2d_{1}^{2}f_{1}h_{1}+Qd_{1}f_{0}+4b_{0}d_{1}d_{2}), \end{array}\\ \begin{array}{l} f_{3}=\frac{1}{Q\alpha^{2}d_{1}-Q\beta^{2}d_{1}-2\beta b_{0}f_{0}-2d_{1}^{2}h_{0}}(2Q\beta d_{1}f_{2}-12\beta b_{0}d_{2}d_{3}+2\beta b_{0}f_{1}f_{2}\\ +6\beta b_{1}d_{1}d_{3}+4\beta b_{2}d_{1}d_{2}+2d_{1}^{2}f_{0}h_{3}+2d_{1}^{2}f_{1}h_{2}+2d_{1}^{2}f_{2}h_{1}+Qd_{1}f_{1}+6b_{0}d_{1}d_{3}\\ +4b_{1}d_{1}d_{2}), \end{array}\\ \begin{array}{l} f_{4}=\frac{1}{Q\alpha^{2}d_{1}-Q\beta^{2}d_{1}-2\beta b_{0}f_{0}-2d_{1}^{2}h_{0}}(2Q\beta d_{1}f_{3}-16\beta b_{0}d_{2}d_{4}-9\beta b_{0}d_{3}^{2}\\ +2\beta b_{0}f_{1}f_{3}+\beta b_{0}f_{2}^{2}+8\beta b_{1}d_{1}d_{4}+6\beta b_{2}d_{1}d_{3}+4\beta b_{3}d_{1}d_{2}+2d_{1}^{2}f_{0}h_{4}+2d_{1}^{2}f_{1}h_{3}\\ +2d_{1}^{2}f_{2}h_{2}+2d_{1}^{2}f_{3}h_{1}+Qd_{1}f_{2}+8b_{0}d_{1}d_{4}+6b_{1}d_{1}d_{3}+4b_{2}d_{1}d_{2}), \end{array}\\ \begin{array}{l} f_{5}=\frac{1}{Q\alpha^{2}d_{1}-Q\beta^{2}d_{1}-2\beta b_{0}f_{0}-2d_{1}^{2}h_{0}}(2Q\beta d_{1}f_{4}-20\beta b_{0}d_{2}d_{5}-24\beta b_{0}d_{3}d_{4}\\ +2\beta b_{0}f_{1}f_{4}+2\beta b_{0}f_{2}f_{3}+10\beta b_{1}d_{1}d_{5}+8\beta b_{2}d_{1}d_{4}+6\beta b_{3}d_{1}d_{3}+4\beta b_{4}d_{1}d_{2}\\ +2d_{1}^{2}f_{0}h_{5}+2d_{1}^{2}f_{1}h_{4}+2d_{1}^{2}f_{2}h_{3}+2d_{1}^{2}f_{3}h_{2}+2d_{1}^{2}f_{4}h_{1}+Qd_{1}f_{3}+10b_{0}d_{1}d_{5}\\ +8b_{1}d_{1}d_{4}+6b_{2}d_{1}d_{3}+4b_{3}d_{1}d_{2}), \end{array}\\ \begin{array}{l} f_{6}=\frac{1}{Q\alpha^{2}d_{1}-Q\beta^{2}d_{1}-2\beta b_{0}f_{0}-2d_{1}^{2}h_{0}}(2Q\beta d_{1}f_{5}-24\beta b_{0}d_{2}d_{6}-30\beta b_{0}d_{3}d_{5}\\ -16\beta b_{0}d_{4}^{2}+2\beta b_{0}f_{1}f_{5}+2\beta b_{0}f_{2}f_{4}+\beta b_{0}f_{3}^{2}+12\beta b_{1}d_{1}d_{6}+10\beta b_{2}d_{1}d_{5}+8\beta b_{3}d_{1}d_{4}\\ +6\beta b_{4}d_{1}d_{3}+4\beta b_{5}d_{1}d_{2}+2d_{1}^{2}f_{0}h_{6}+2d_{1}^{2}f_{1}h_{5}+2d_{1}^{2}f_{2}h_{4}+2d_{1}^{2}f_{3}h_{3}+2d_{1}^{2}f_{4}h_{2}\\ +2d_{1}^{2}f_{5}h_{1}+Qd_{1}f_{4}+12b_{0}d_{1}d_{6}+10b_{1}d_{1}d_{5}+8b_{2}d_{1}d_{4}+6b_{3}d_{1}d_{3}+4b_{4}d_{1}d_{2}), \end{array}\\ \begin{array}{l} f_{7}=\frac{1}{Q\alpha^{2}d_{1}-Q\beta^{2}d_{1}-2\beta b_{0}f_{0}-2d_{1}^{2}h_{0}}(2Q\beta d_{1}f_{6}-28\beta b_{0}d_{2}d_{7}-36\beta b_{0}d_{3}d_{6}\\ -40\beta b_{0}d_{4}d_{5}+2\beta b_{0}f_{1}f_{6}+2\beta b_{0}f_{2}f_{5}+2\beta b_{0}f_{3}f_{4}+14\beta b_{1}d_{1}d_{7}+12\beta b_{2}d_{1}d_{6}\\ +10\beta b_{3}d_{1}d_{5}+8\beta b_{4}d_{1}d_{4}+6\beta b_{5}d_{1}d_{3}+4\beta b_{6}d_{1}d_{2}+2d_{1}^{2}f_{0}h_{7}+2d_{1}^{2}f_{1}h_{6}+2d_{1}^{2}f_{2}h_{5}\\ +2d_{1}^{2}f_{3}h_{4}+2d_{1}^{2}f_{4}h_{3}+2d_{1}^{2}f_{5}h_{2}+2d_{1}^{2}f_{6}h_{1}+Qd_{1}f_{5}+14b_{0}d_{1}d_{7}+12b_{1}d_{1}d_{6}\\ +10b_{2}d_{1}d_{5}+8b_{3}d_{1}d_{4}+6b_{4}d_{1}d_{3}+4b_{5}d_{1}d_{2}), \end{array}\\ \begin{array}{l} f_{8}=\frac{1}{Q\alpha^{2}d_{1}-Q\beta^{2}d_{1}-2\beta b_{0}f_{0}-2d_{1}^{2}h_{0}}(2d_{1}^{2}f_{0}h_{8}+2d_{1}^{2}f_{2}h_{6}+2d_{1}^{2}f_{3}h_{5}+2d_{1}^{2}f_{4}h_{4}\\ +2d_{1}^{2}f_{5}h_{3}+2d_{1}^{2}f_{6}h_{2}+2d_{1}^{2}f_{7}h_{1}-25\beta b_{0}d_{5}^{2}+\beta b_{0}f_{4}^{2}+d_{1}Qf_{6}+16d_{1}b_{0}d_{8}+14d_{1}b_{1}d_{7}\\ +12d_{1}b_{2}d_{6}+10d_{1}b_{3}d_{5}+8d_{1}b_{4}d_{4}+6d_{1}b_{5}d_{3}+4d_{1}b_{6}d_{2}+2d_{1}^{2}f_{1}h_{7}+2d_{1}Q\beta f_{7}\\ +16d_{1}\beta b_{1}d_{8}+14d_{1}\beta b_{2}d_{7}+12d_{1}\beta b_{3}d_{6}+10d_{1}\beta b_{4}d_{5}+8d_{1}\beta b_{5}d_{4}+6d_{1}\beta b_{6}d_{3}\\ +4d_{1}\beta b_{7}d_{2}-32\beta b_{0}d_{2}d_{8}-42\beta b_{0}d_{3}d_{7}-48\beta b_{0}d_{4}d_{6}+2\beta b_{0}f_{1}f_{7}+2\beta b_{0}f_{2}f_{6}\\ +2\beta b_{0}f_{3}f_{5}), \end{array}\\ \begin{array}{l} f_{9}=\frac{1}{Q\alpha^{2}d_{1}-Q\beta^{2}d_{1}-2\beta b_{0}f_{0}-2d_{1}^{2}h_{0}}(8d_{1}\beta b_{6}d_{4}+6d_{1}\beta b_{7}d_{3}+4d_{1}\beta b_{8}d_{2}\\ -36\beta b_{0}d_{2}d_{9}+2\beta b_{0}f_{3}f_{6}-48\beta b_{0}d_{3}d_{8}-56\beta b_{0}d_{4}d_{7}-60\beta b_{0}d_{5}d_{6}+2d_{1}Q\beta f_{8}\\ +18d_{1}\beta b_{1}d_{9}+16d_{1}\beta b_{2}d_{8}+14d_{1}\beta b_{3}d_{7}+12d_{1}\beta b_{4}d_{6}+10d_{1}\beta b_{5}d_{5}+2\beta b_{0}f_{4}f_{5}\\ +2\beta b_{0}f_{1}f_{8}+2\beta b_{0}f_{2}f_{7}+14d_{1}b_{2}d_{7}+12d_{1}b_{3}d_{6}+10d_{1}b_{4}d_{5}+8d_{1}b_{5}d_{4}+6d_{1}b_{6}d_{3}\\ +4d_{1}b_{7}d_{2}+2d_{1}^{2}f_{1}h_{8}+2d_{1}^{2}f_{2}h_{7}+2d_{1}^{2}f_{0}h_{9}+2d_{1}^{2}f_{3}h_{6}+2d_{1}^{2}f_{4}h_{5}+2d_{1}^{2}f_{5}h_{4}\\ +2d_{1}^{2}f_{6}h_{3}+2d_{1}^{2}f_{7}h_{2}+2d_{1}^{2}f_{8}h_{1}+d_{1}Qf_{7}+18d_{1}b_{0}d_{9}+16d_{1}b_{1}d_{8}), \end{array}\\ \begin{array}{l} f_{10}=\frac{1}{Q\alpha^{2}d_{1}-Q\beta^{2}d_{1}-2\beta b_{0}f_{0}-2d_{1}^{2}h_{0}}(2d_{1}^{2}f_{5}h_{5}+2d_{1}^{2}f_{6}h_{4}+2d_{1}^{2}f_{7}h_{3}+2d_{1}^{2}f_{8}h_{2}\\ +2d_{1}^{2}f_{9}h_{1}-36\beta b_{0}d_{6}^{2}+\beta b_{0}f_{5}^{2}+4d_{1}b_{8}d_{2}+d_{1}Qf_{8}+20d_{1}b_{0}d_{10}+18d_{1}b_{1}d_{9}\\ +16d_{1}b_{2}d_{8}+14d_{1}b_{3}d_{7}+12d_{1}b_{4}d_{6}+10d_{1}b_{5}d_{5}+8d_{1}b_{6}d_{4}+6d_{1}b_{7}d_{3}+2d_{1}^{2}f_{0}h_{10}\\ +2d_{1}^{2}f_{1}h_{9}+2d_{1}^{2}f_{2}h_{8}+2d_{1}^{2}f_{3}h_{7}+2d_{1}^{2}f_{4}h_{6}+2d_{1}Q\beta f_{9}+20d_{1}\beta b_{1}d_{10}+18d_{1}\beta b_{2}d_{9}\\ +16d_{1}\beta b_{3}d_{8}+14d_{1}\beta b_{4}d_{7}+12d_{1}\beta b_{5}d_{6}+10d_{1}\beta b_{6}d_{5}+8d_{1}\beta b_{7}d_{4}+6d_{1}\beta b_{8}d_{3}\\ +4d_{1}\beta b_{9}d_{2}-40\beta b_{0}d_{2}d_{10}-54\beta b_{0}d_{3}d_{9}-64\beta b_{0}d_{4}d_{8}-70\beta b_{0}d_{5}d_{7}+2\beta b_{0}f_{1}f_{9}\\ +2\beta b_{0}f_{2}f_{8}+2\beta b_{0}f_{3}f_{7}+2\beta b_{0}f_{4}f_{6}), \end{array}\\ \begin{array}{l} f_{11}=\frac{1}{Q\alpha^{2}d_{1}-Q\beta^{2}d_{1}-2\beta b_{0}f_{0}-2d_{1}^{2}h_{0}}(2d_{1}^{2}f_{0}h_{11}+d_{1}Qf_{9}+22d_{1}b_{0}d_{11}+20d_{1}b_{1}d_{10}\\ +18d_{1}b_{2}d_{9}+16d_{1}b_{3}d_{8}+14d_{1}b_{4}d_{7}+12d_{1}b_{5}d_{6}+10d_{1}b_{6}d_{5}+8d_{1}b_{7}d_{4}+6d_{1}b_{8}d_{3}\\ +4d_{1}b_{9}d_{2}+2d_{1}^{2}f_{1}h_{10}+2d_{1}^{2}f_{2}h_{9}+2d_{1}^{2}f_{3}h_{8}+2d_{1}^{2}f_{4}h_{7}+2d_{1}^{2}f_{5}h_{6}+2d_{1}^{2}f_{6}h_{5}+2d_{1}^{2}f_{7}h_{4}\\ +2d_{1}^{2}f_{8}h_{3}+2d_{1}^{2}f_{9}h_{2}+2d_{1}^{2}f_{10}h_{1}+2\beta b_{0}f_{4}f_{7}+2\beta b_{0}f_{5}f_{6}+2d_{1}Q\beta f_{10}+22d_{1}\beta b_{1}d_{11}\\ +20d_{1}\beta b_{2}d_{10}+18d_{1}\beta b_{3}d_{9}+16d_{1}\beta b_{4}d_{8}+14d_{1}\beta b_{5}d_{7}+12d_{1}\beta b_{6}d_{6}+10d_{1}\beta b_{7}d_{5}\\ +8d_{1}\beta b_{8}d_{4}+6d_{1}\beta b_{9}d_{3}+4d_{1}\beta b_{10}d_{2}-44\beta b_{0}d_{2}d_{11}-60\beta b_{0}d_{3}d_{10}-72\beta b_{0}d_{4}d_{9}\\ -80\beta b_{0}d_{5}d_{8}-84\beta b_{0}d_{6}d_{7}+2\beta b_{0}f_{1}f_{10}+2\beta b_{0}f_{2}f_{9}+2\beta b_{0}f_{3}f_{8}), \end{array}\\ \begin{array}{l} f_{12}=\frac{1}{Q\alpha^{2}d_{1}-Q\beta^{2}d_{1}-2\beta b_{0}f_{0}-2d_{1}^{2}h_{0}}(2d_{1}^{2}f_{8}h_{4}+2d_{1}^{2}f_{9}h_{3}+2d_{1}^{2}f_{10}h_{2}+2d_{1}^{2}f_{11}h_{1}\\ -49\beta b_{0}d_{7}^{2}+\beta b_{0}f_{6}^{2}-66\beta b_{0}d_{3}d_{11}-80\beta b_{0}d_{4}d_{10}-90\beta b_{0}d_{5}d_{9}-96\beta b_{0}d_{6}d_{8}+2\beta b_{0}f_{1}f_{11}\\ +2\beta b_{0}f_{2}f_{10}+2\beta b_{0}f_{3}f_{9}+2\beta b_{0}f_{4}f_{8}+2\beta b_{0}f_{5}f_{7}+2d_{1}Q\beta f_{11}+24d_{1}\beta b_{1}d_{12}\\ +22d_{1}\beta b_{2}d_{11}+20d_{1}\beta b_{3}d_{10}+18d_{1}\beta b_{4}d_{9}+16d_{1}\beta b_{5}d_{8}+14d_{1}\beta b_{6}d_{7}+12d_{1}\beta b_{7}d_{6}\\ +10d_{1}\beta b_{8}d_{5}+8d_{1}\beta b_{9}d_{4}+6d_{1}\beta b_{10}d_{3}+4d_{1}\beta b_{11}d_{2}+2d_{1}^{2}f_{0}h_{12}+18d_{1}b_{3}d_{9}+16d_{1}b_{4}d_{8}\\ +14d_{1}b_{5}d_{7}+12d_{1}b_{6}d_{6}+10d_{1}b_{7}d_{5}+8d_{1}b_{8}d_{4}+6d_{1}b_{9}d_{3}+4d_{1}b_{10}d_{2}+d_{1}Qf_{10}+24d_{1}b_{0}d_{12}\\ +22d_{1}b_{1}d_{11}+20d_{1}b_{2}d_{10}+2d_{1}^{2}f_{1}h_{11}+2d_{1}^{2}f_{2}h_{10}+2d_{1}^{2}f_{3}h_{9}+2d_{1}^{2}f_{4}h_{8}+2d_{1}^{2}f_{5}h_{7}\\ +2d_{1}^{2}f_{6}h_{6}+2d_{1}^{2}f_{7}h_{5}-48\beta b_{0}d_{2}d_{12}), \end{array}\\ h_{0}=\frac{1}{2\sqrt{-\frac{1}{\left(Q\alpha^{2}-Q\beta^{2}-2\beta b_{0})(Q\alpha^{2}-Q\beta^{2}+2\beta b_{0}\right)}},}\\ h_{1}=2\beta b_{0}c_{0}\sqrt{-\frac{1}{Q^{2}\alpha^{4}-2Q^{2}\alpha^{2}\beta^{2}+Q^{2}\beta^{4}-4\beta^{2}b_{0}^{2}}},\\ h_{2}=\frac{c_{0}f_{1}}{2}+\frac{c_{1}f_{0}}{2},\\ h_{3}=\frac{c_{0}f_{2}}{3}+\frac{c_{1}f_{1}}{3}+\frac{c_{2}f_{0}}{3},\\ h_{4}=\frac{c_{0}f_{3}}{4}+\frac{c_{1}f_{2}}{4}+\frac{c_{2}f_{1}}{4}+\frac{c_{3}f_{0}}{4},\\ h_{5}=\frac{c_{0}f_{4}}{5}+\frac{c_{1}f_{3}}{5}+\frac{c_{2}f_{2}}{5}+\frac{c_{3}f_{1}}{5}+\frac{c_{4}f_{0}}{5},\\ h_{6}=\frac{c_{0}f_{5}}{6}+\frac{c_{1}f_{4}}{6}+\frac{c_{2}f_{3}}{6}+\frac{c_{3}f_{2}}{6}+\frac{c_{4}f_{1}}{6}+\frac{c_{5}f_{0}}{6},\\ h_{7}=\frac{c_{0}f_{6}}{7}+\frac{c_{1}f_{5}}{7}+\frac{c_{2}f_{4}}{7}+\frac{c_{3}f_{3}}{7}+\frac{c_{4}f_{2}}{7}+\frac{c_{5}f_{1}}{7}+\frac{c_{6}f_{0}}{7},\\ h_{8}=\frac{c_{0}f_{7}}{8}+\frac{c_{1}f_{6}}{8}+\frac{c_{2}f_{5}}{8}+\frac{c_{3}f_{4}}{8}+\frac{c_{4}f_{3}}{8}+\frac{c_{5}f_{2}}{8}+\frac{c_{6}f_{1}}{8}+\frac{c_{7}f_{0}}{8},\\ h_{9}=\frac{c_{0}f_{8}}{9}+\frac{c_{1}f_{7}}{9}+\frac{c_{2}f_{6}}{9}+\frac{c_{3}f_{5}}{9}+\frac{c_{4}f_{4}}{9}+\frac{c_{5}f_{3}}{9}+\frac{c_{6}f_{2}}{9}+\frac{c_{7}f_{1}}{9}+\frac{c_{8}f_{0}}{9},\\ h_{10}=\frac{c_{0}f_{9}}{10}+\frac{c_{1}f_{8}}{10}+\frac{c_{2}f_{7}}{10}+\frac{c_{3}f_{6}}{10}+\frac{c_{4}f_{5}}{10}+\frac{c_{5}f_{4}}{10}+\frac{c_{6}f_{3}}{10}+\frac{c_{7}f_{2}}{10}+\frac{c_{8}f_{1}}{10}+\frac{c_{9}f_{0}}{10},\\ h_{11}=\frac{c_{0}f_{10}}{11}+\frac{c_{1}f_{9}}{11}+\frac{c_{2}f_{8}}{11}+\frac{c_{3}f_{7}}{11}+\frac{c_{4}f_{6}}{11}+\frac{c_{5}f_{5}}{11}+\frac{c_{6}f_{4}}{11}+\frac{c_{7}f_{3}}{11}+\frac{c_{8}f_{2}}{11}+\frac{c_{9}f_{1}}{11}+\frac{c_{10}f_{0}}{11},\\ \begin{array}{l} h_{12}=\frac{c_{0}f_{11}}{12}+\frac{c_{1}f_{10}}{12}+\frac{c_{2}f_{9}}{12}+\frac{c_{3}f_{8}}{12}+\frac{c_{4}f_{7}}{12}+\frac{c_{5}f_{6}}{12}+\frac{c_{6}f_{5}}{12}+\frac{c_{7}f_{4}}{12}+\frac{c_{8}f_{3}}{12}+\frac{c_{9}f_{2}}{12}\\ +\frac{c_{10}f_{1}}{12}+\frac{c_{11}f_{0}}{12}. \end{array} \end{array}$$
